# Supplementary material for: Development of New Pyrazolo [3,4-b]Pyridine Derivatives as Potent Anti-Leukemic Agents and Topoisomerase IIα Inhibitors with Broad-Spectrum Cytotoxicity
Source: Pharmaceuticals (Basel). 2025 Nov 20;18(11):1770. doi: 10.3390/ph18111770 (PMC12655695; doi:10.3390/ph18111770)
Supplement: Supplementary file 1 [file pharmaceuticals-18-01770-s001.zip › Supporting Information-R1.pdf]

Supporting Information

Development of new pyrazolo[3,4-*b*]pyridine derivatives as potent anti-leukemic agents and Topoisomerase IIα inhibitors with broad-spectrum cytotoxicity

Wagdy M. Eldehna [a,1,*], Haytham O. Tawfik [b,1], Denisa Veselá [c], Veronika Vojáčková [c], Ahmed T. Negmeldin [d,e,*], Zainab M. Elsayed [f], Taghreed A Majrashi [g], Petra Krňávková [c], Mostafa M. Elbadawi [a], Moataz A. Shaldam [a], Ghada H. Al-Ansary [h], Vladimír Kryštof [c,i], Hatem A. Abdel-Aziz [j,*]

[a] *Department of Pharmaceutical Chemistry, Faculty of Pharmacy, Kafrelsheikh University, P.O. Box 33516, Kafrelsheikh, Egypt*
[b] *Department of Pharmaceutical Chemistry, Faculty of Pharmacy, Tanta University, Tanta, 31527, Egypt*
[c] *Department of Experimental Biology, Faculty of Science, Palacký University Olomouc, Šlechtitelů 27, 77900 Olomouc, Czech Republic*
[d] *Department of Pharmaceutical Sciences, College of Pharmacy and Thumbay Research Institute for Precision Medicine, Gulf Medical University, Ajman, United Arab Emirates*
[e] *Department of Pharmaceutical Organic Chemistry, Faculty of Pharmacy, Cairo University, Cairo, Egypt*
[f] *Scientific Research and Innovation Support Unit, Faculty of Pharmacy, Kafrelsheikh University, Kafrelsheikh, Egypt*
[g] *Department of Pharmacognosy, College of Pharmacy, King Khalid University, Asir, Saudi Arabia*
[h] *Department of Pharmaceutical Chemistry, Faculty of Pharmacy, Ain Shams University, Abassia, Cairo 11566, Egypt*
[i] *Institute of Molecular and Translational Medicine, Faculty of Medicine and Dentistry, Palacký University Olomouc, Hněvotínská 5, 77900 Olomouc, Czech Republic*
[j] *Applied Organic Chemistry Department, National Research Center, Dokki 12622, Cairo, Egypt*

*Corresponding author: W. M. Eldehna wagdy2000@gmail.com, A. T. Negmeldin dr.ahmedthabet@gmu.ac.ae and H. A. Abdel-Aziz hatem_741@yahoo.com

[1] Both authors contributed equally to this work.

Supporting Information

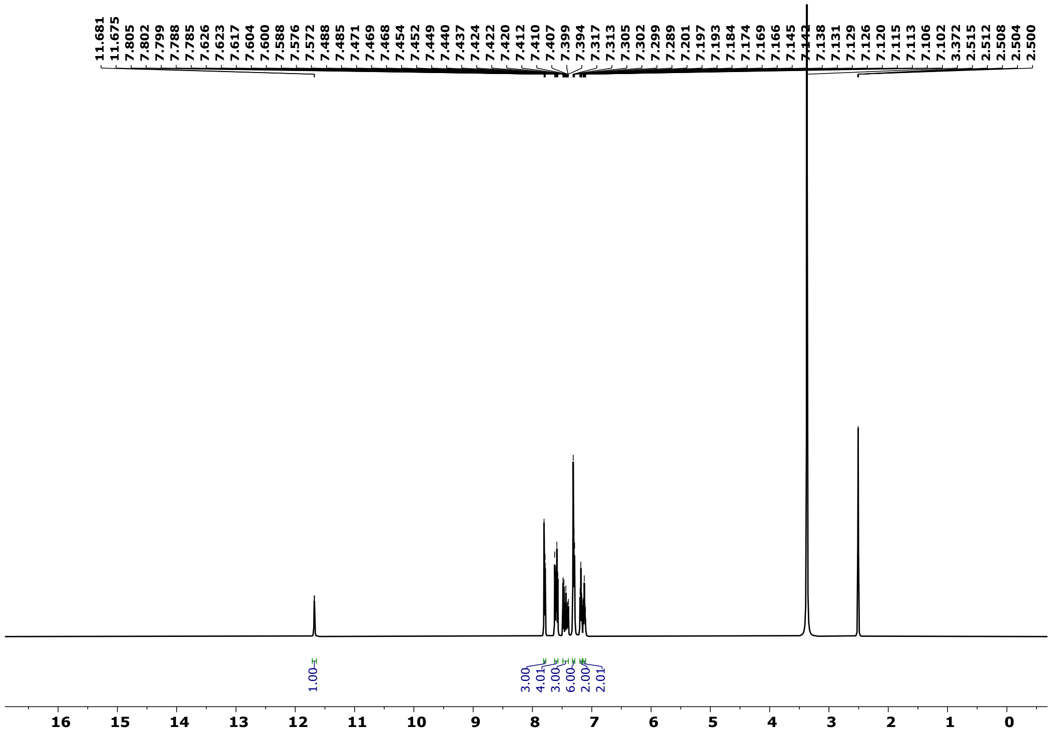

Figure S1. ^{1}H NMR (500 MHz, DMSO-d_6) spectrum of compound **8a**

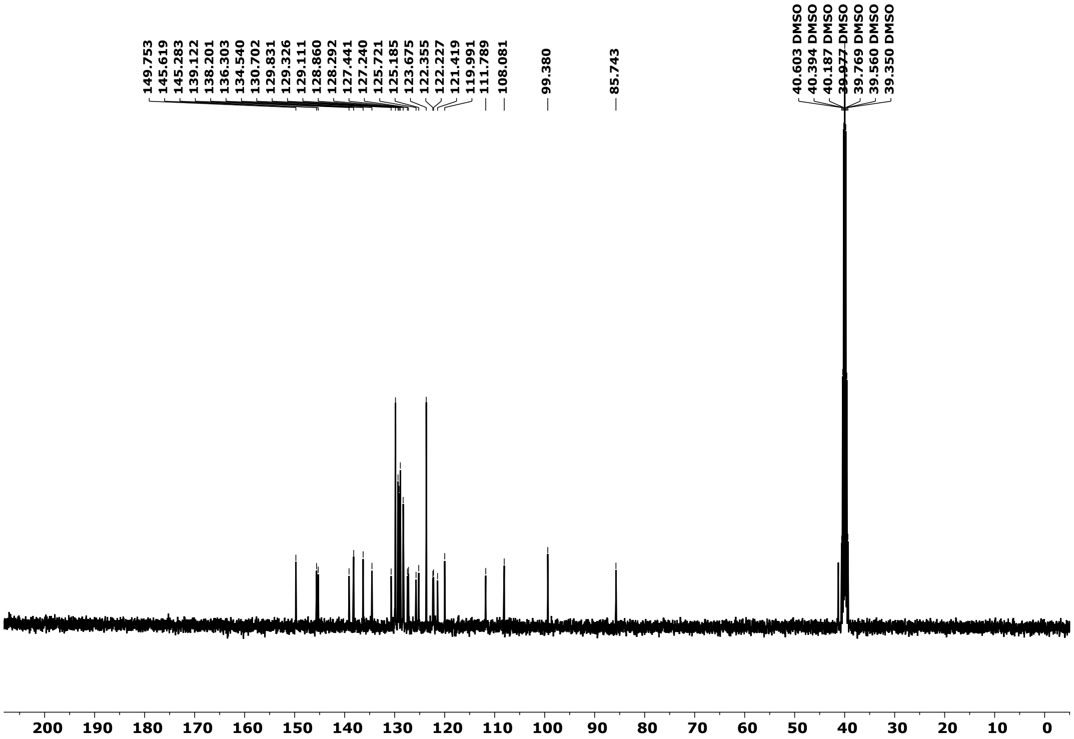

Figure S2. ^{13}C NMR (101 MHz, DMSO-d_6) spectrum of compound **8a**

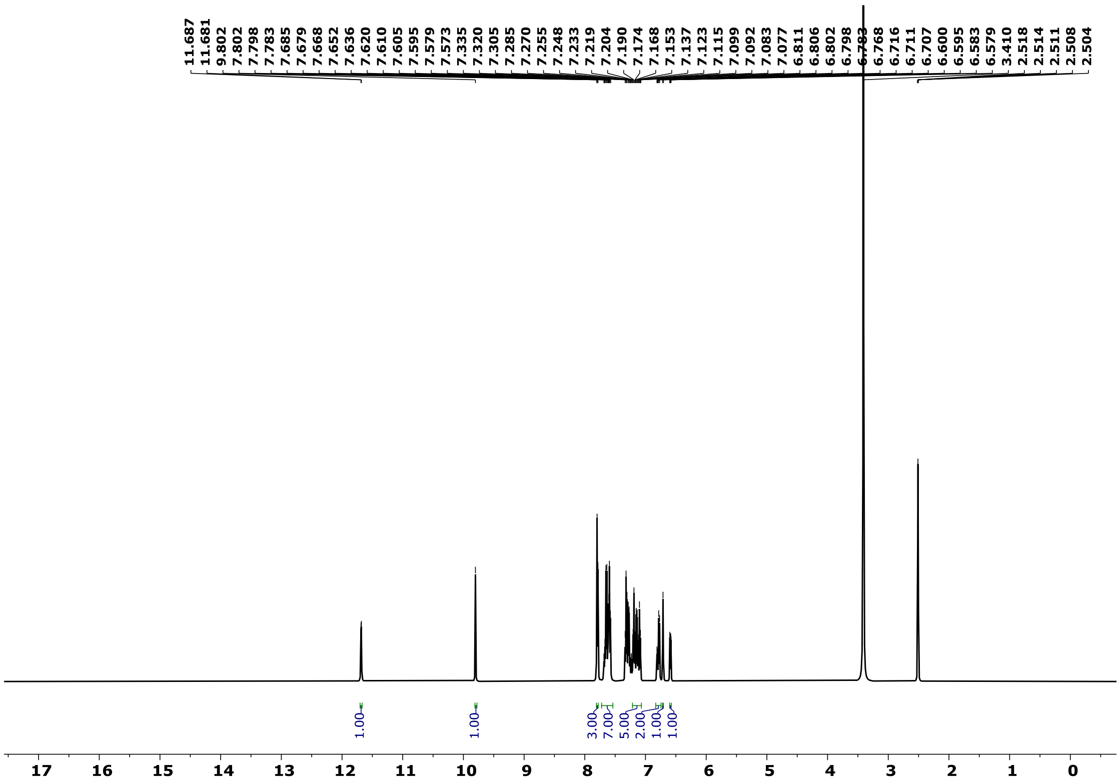

Figure S3. ^{1}H NMR (500 MHz, DMSO-d_6) spectrum of compound **8b**

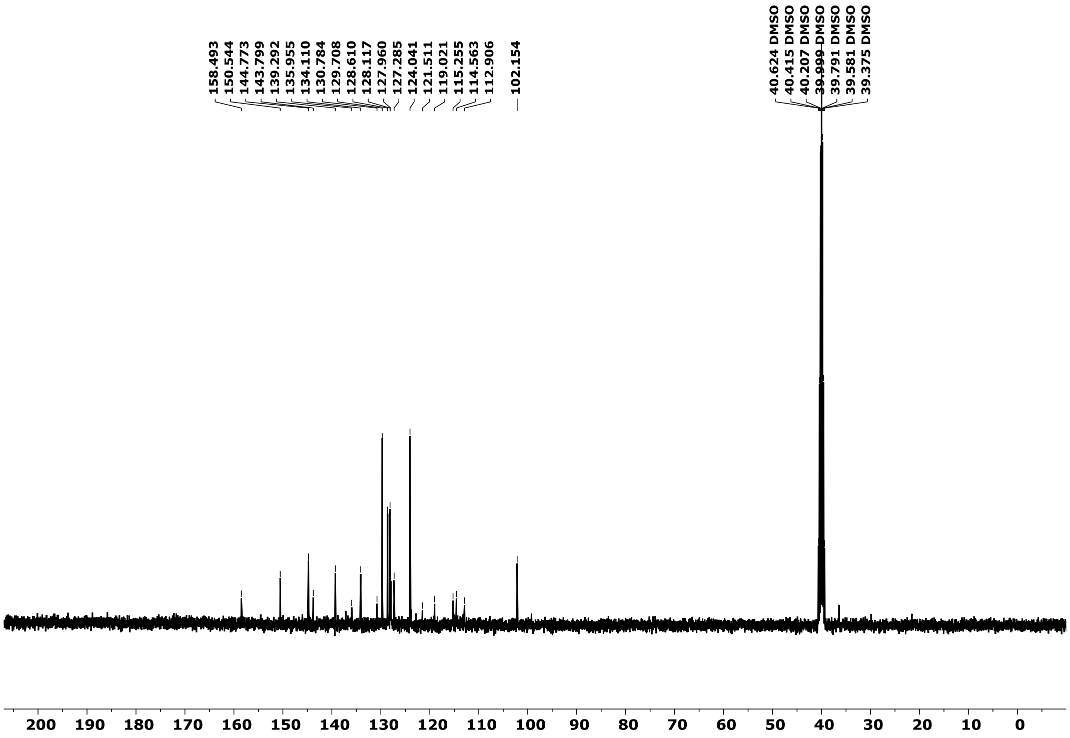

Figure S4. ^{13}C NMR (101 MHz, DMSO-d_6) spectrum of compound **8b**

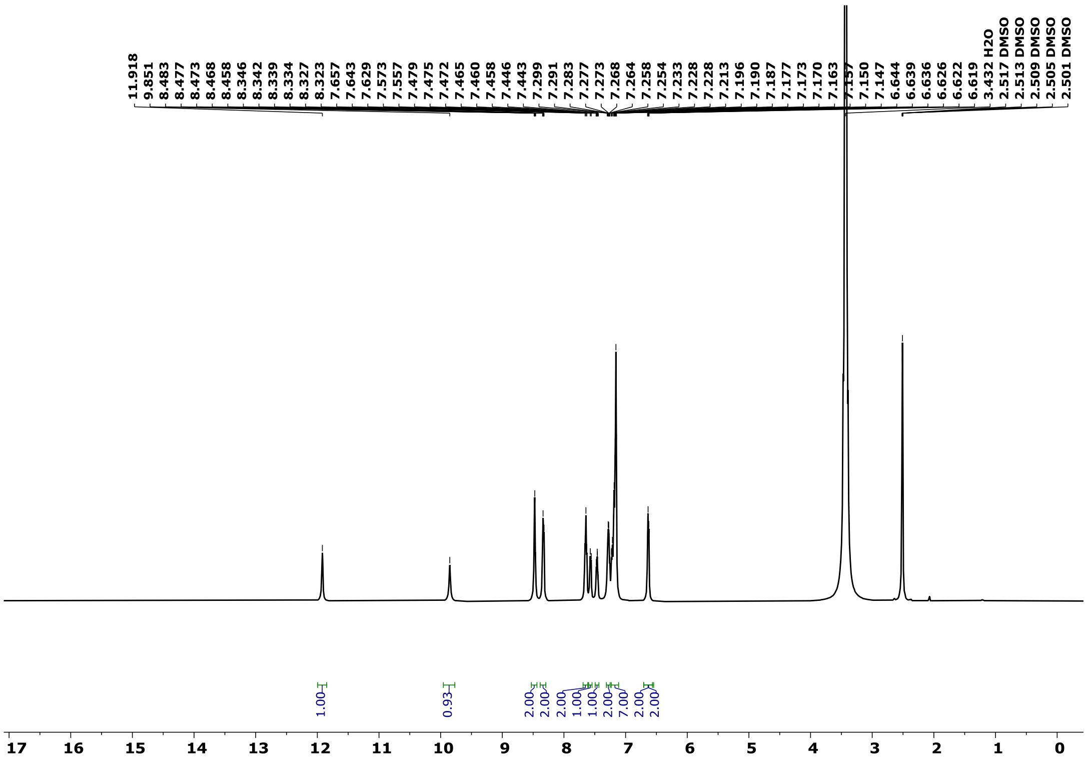

Figure S5. ^{1}H NMR (500 MHz, DMSO-d_6) spectrum of compound **8c**

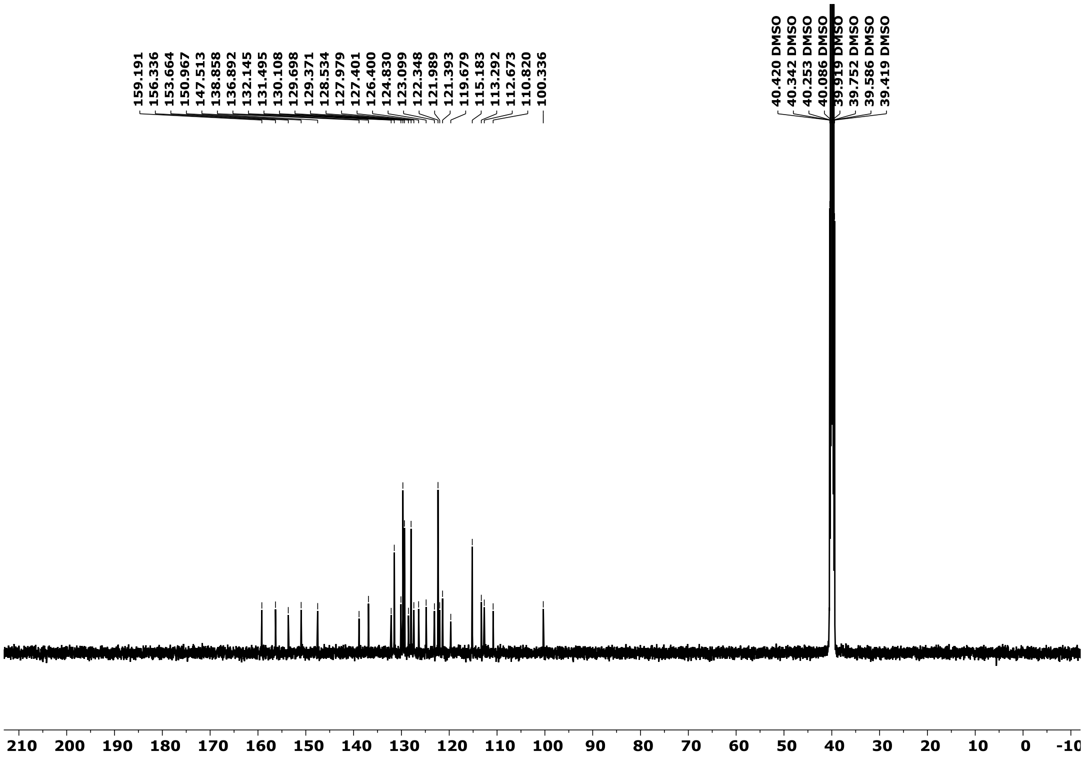

Figure S6. ^{13}C NMR (126 MHz, DMSO-d_6) spectrum of compound **8c**

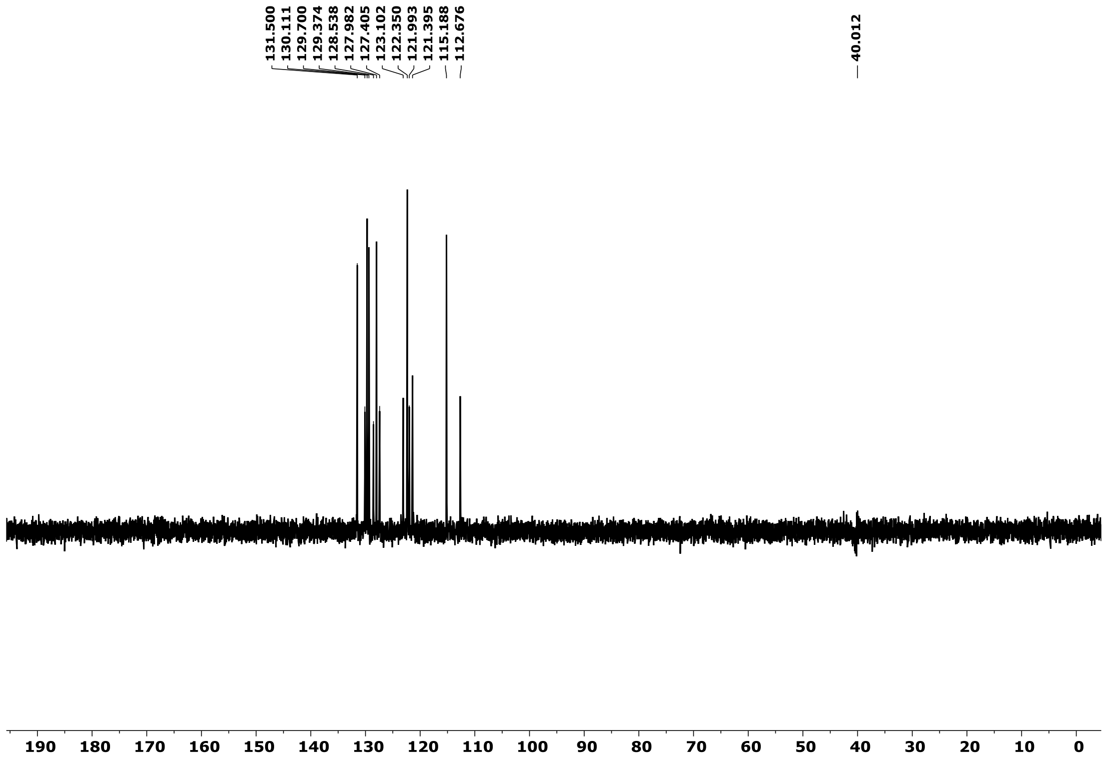

Figure S7. DEPT-135 (126 MHz, DMSO-d_6) spectrum of compound **8c**

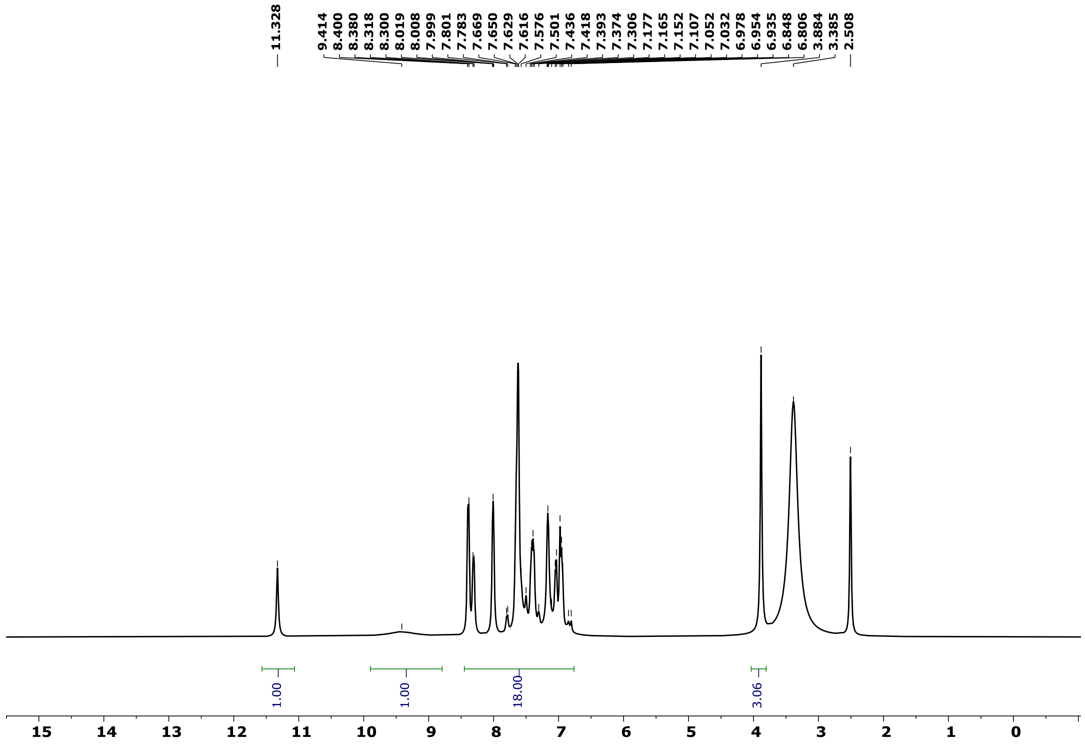

Figure S8. ^{1}H NMR (400 MHz, DMSO-d_6) spectrum of compound **8d**

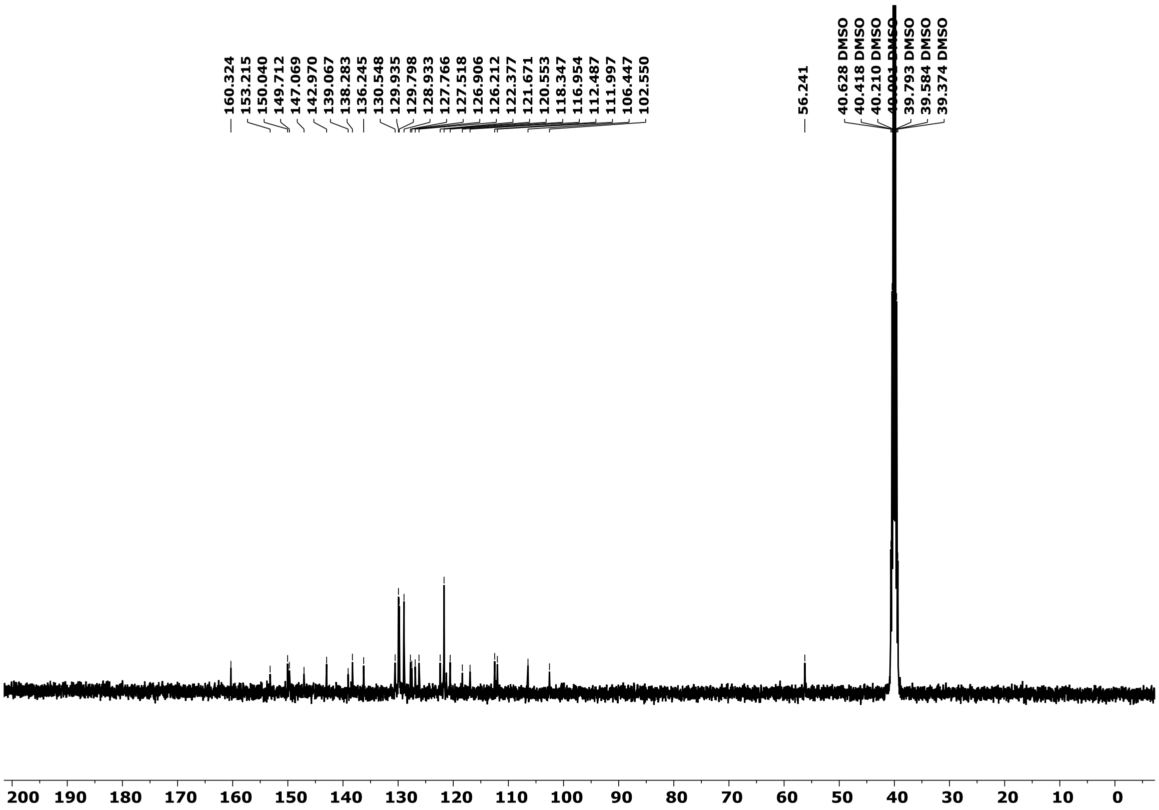

Figure S9. ^{13}C NMR (101MHz, DMSO-d_6) spectrum of compound **8d**

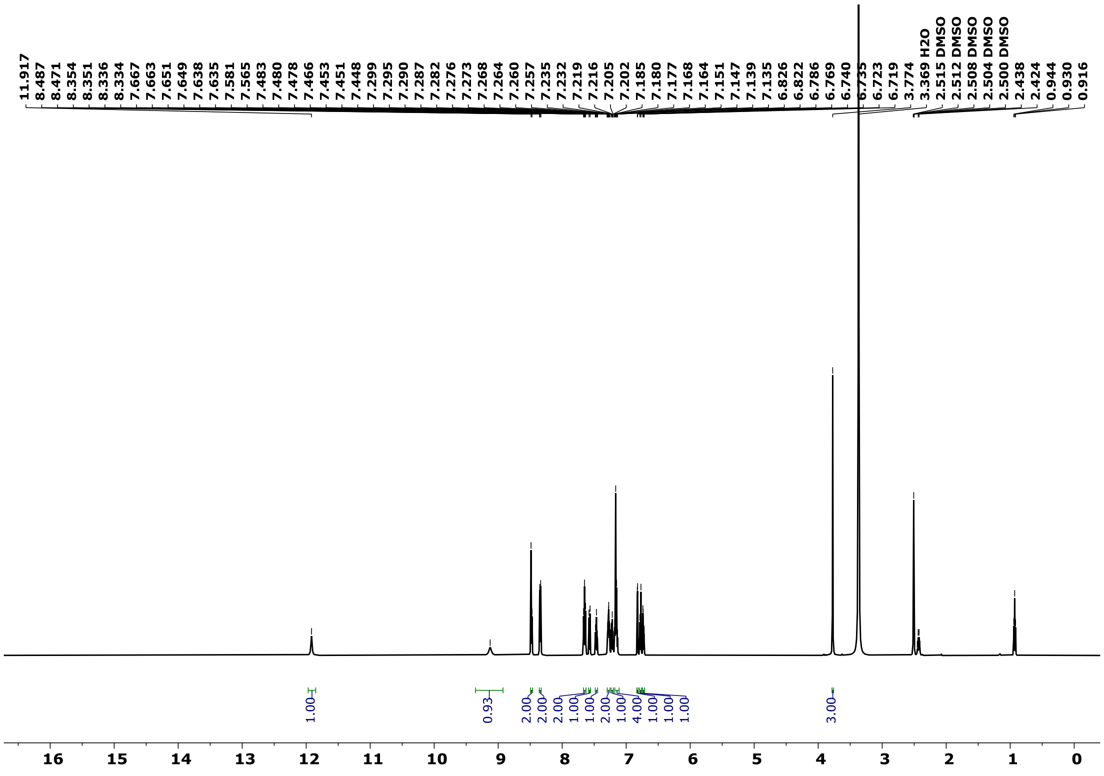

Figure S10. ^{1}H NMR (500 MHz, DMSO-d_6) spectrum of compound **8e**

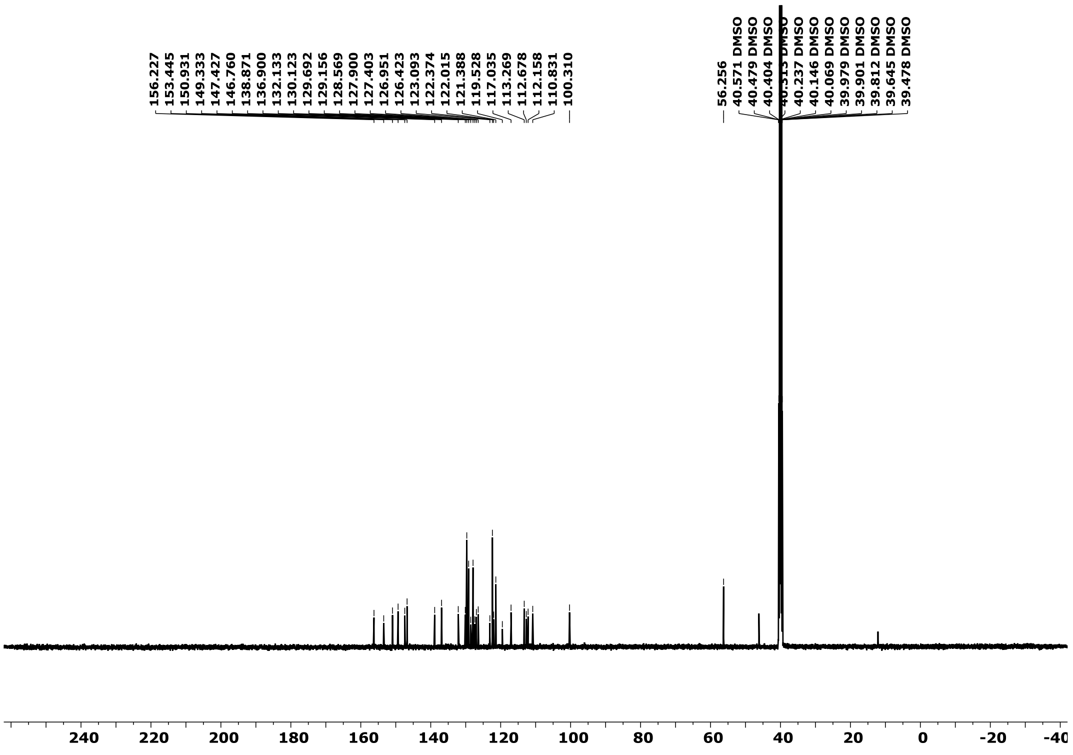

Figure S11. ^{13}C NMR (126 MHz, DMSO-d_6) spectrum of compound **8e**

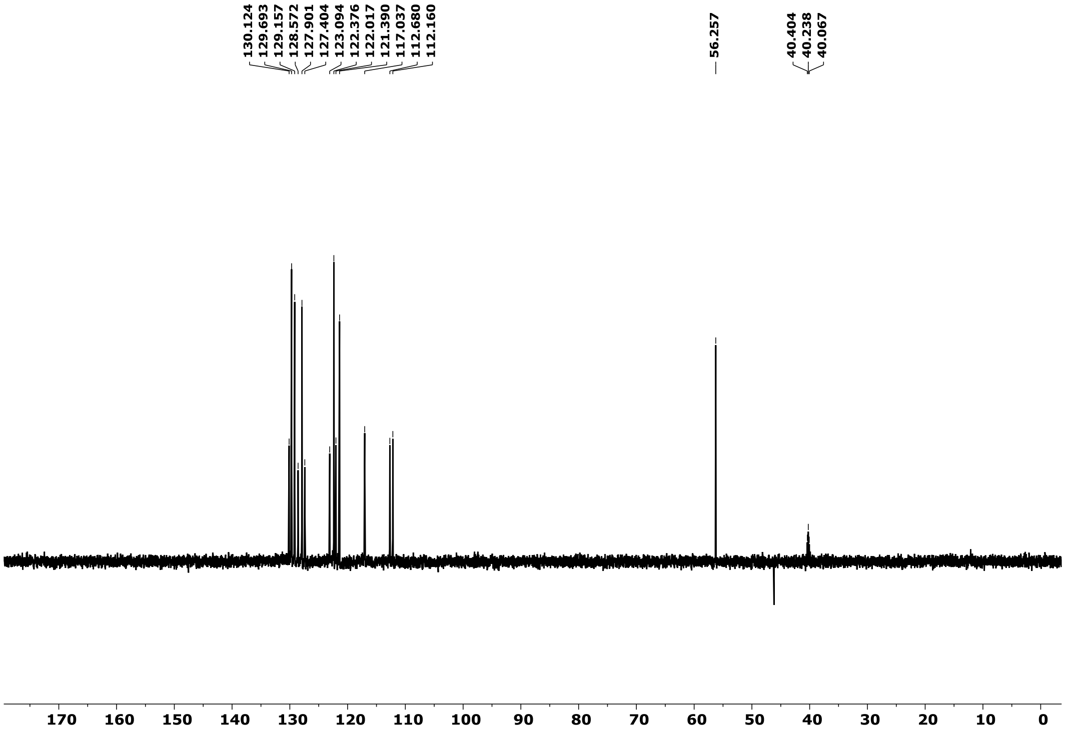

Figure S12. DEPT-135 (126 MHz, DMSO-d_6) spectrum of compound **8e**

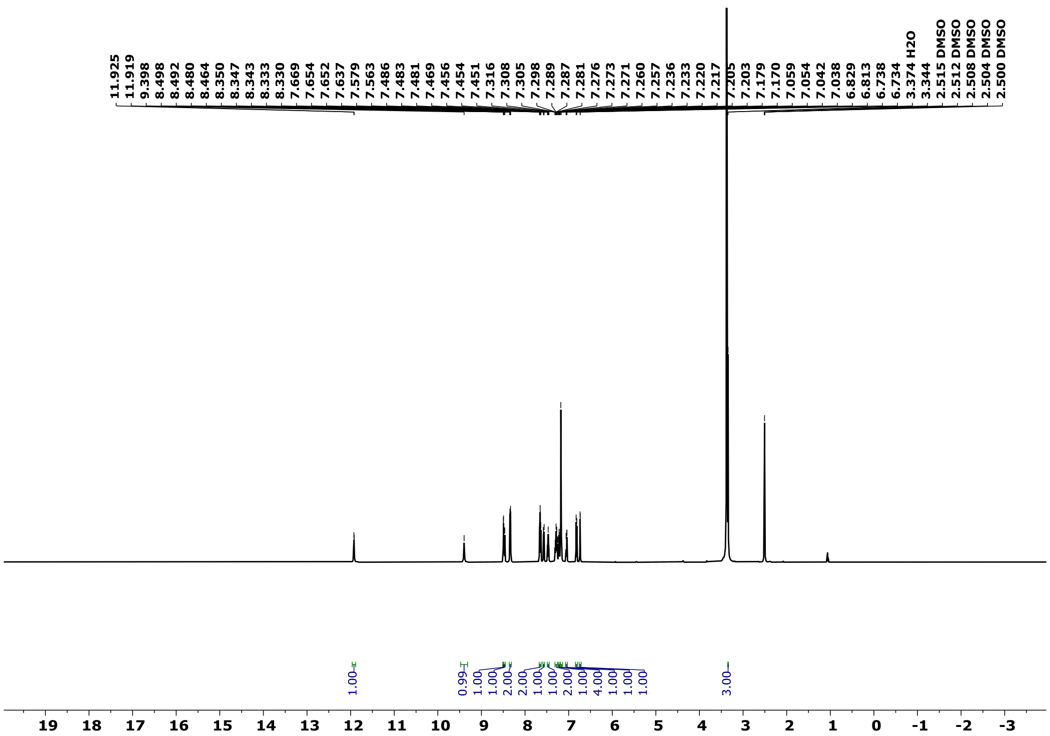

Figure S13. ^{1}H NMR (500 MHz, DMSO-d_6) spectrum of compound **8f**

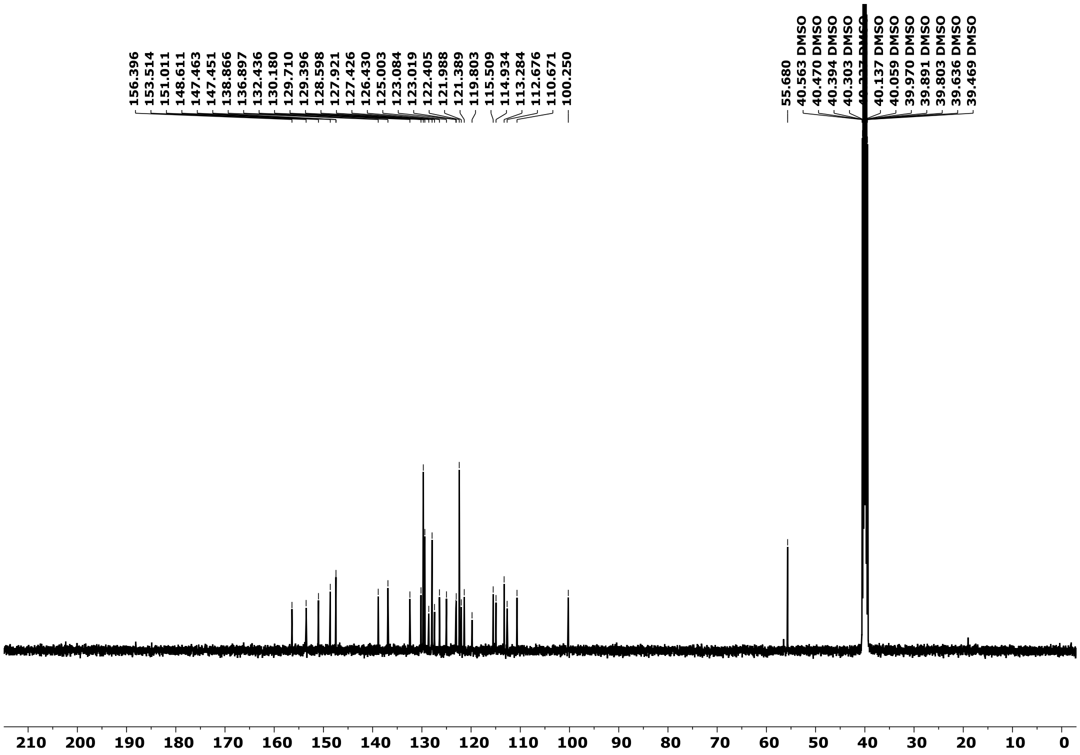

Figure S14. ^{13}C NMR (126 MHz, DMSO-d_6) spectrum of compound **8f**

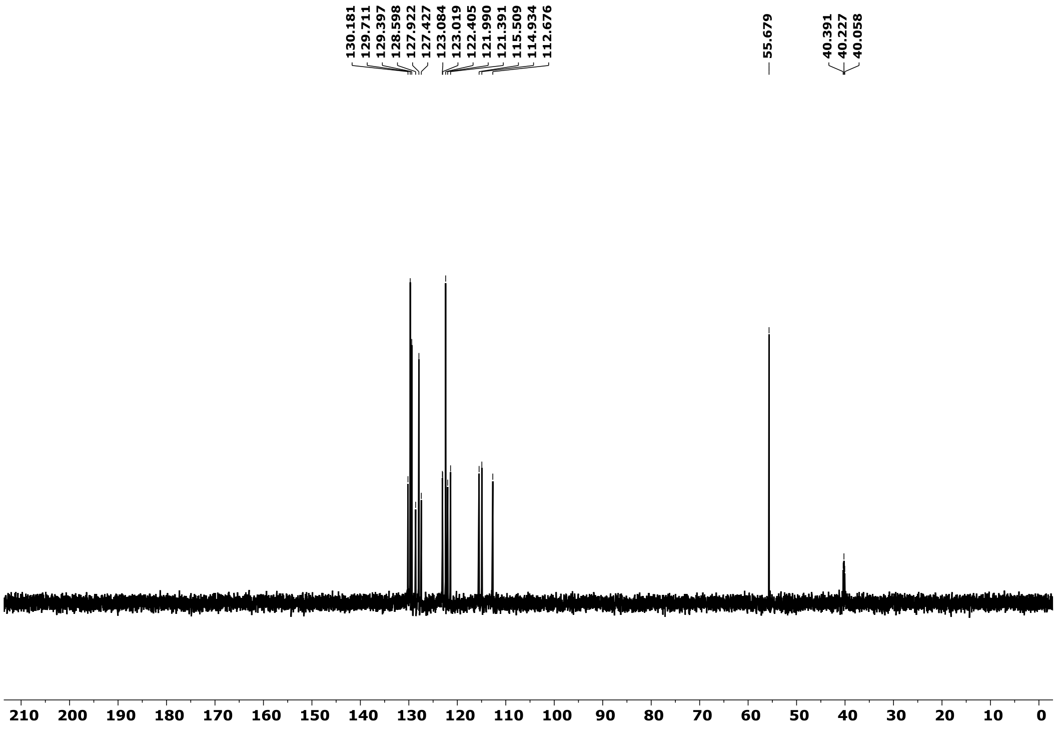

Figure S15. DEPT-135 (126 MHz, DMSO-d_6) spectrum of compound **8f**

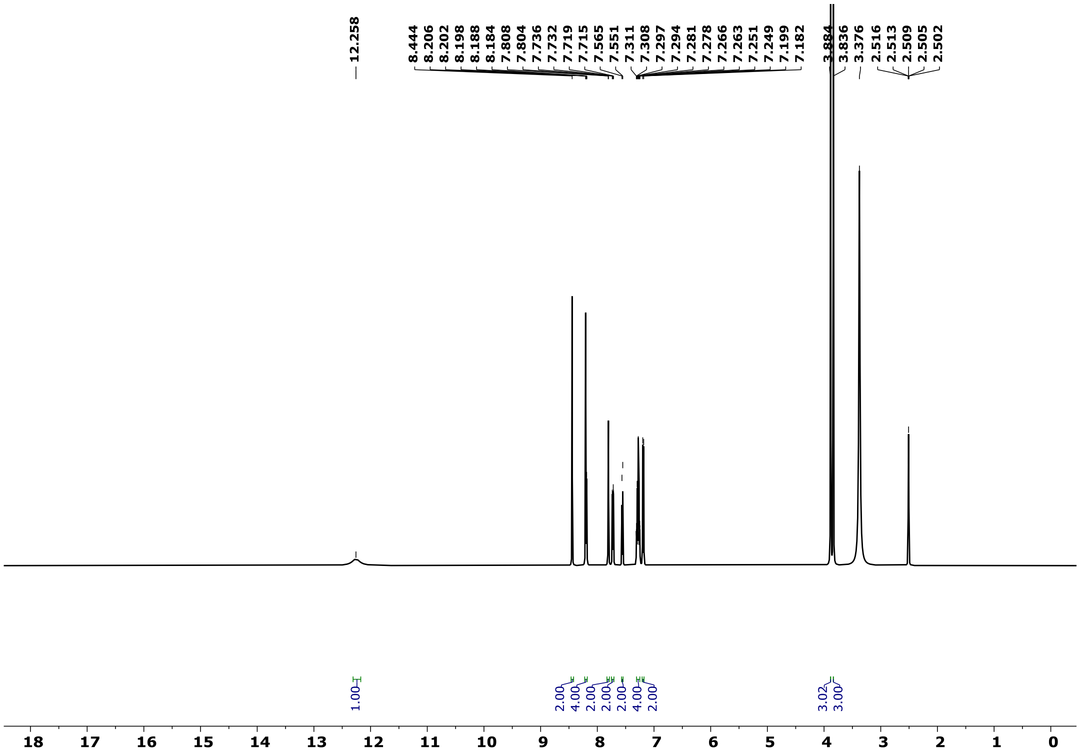

Figure S16. ^{1}H NMR (500 MHz, DMSO-d_6) spectrum of compound **8g**

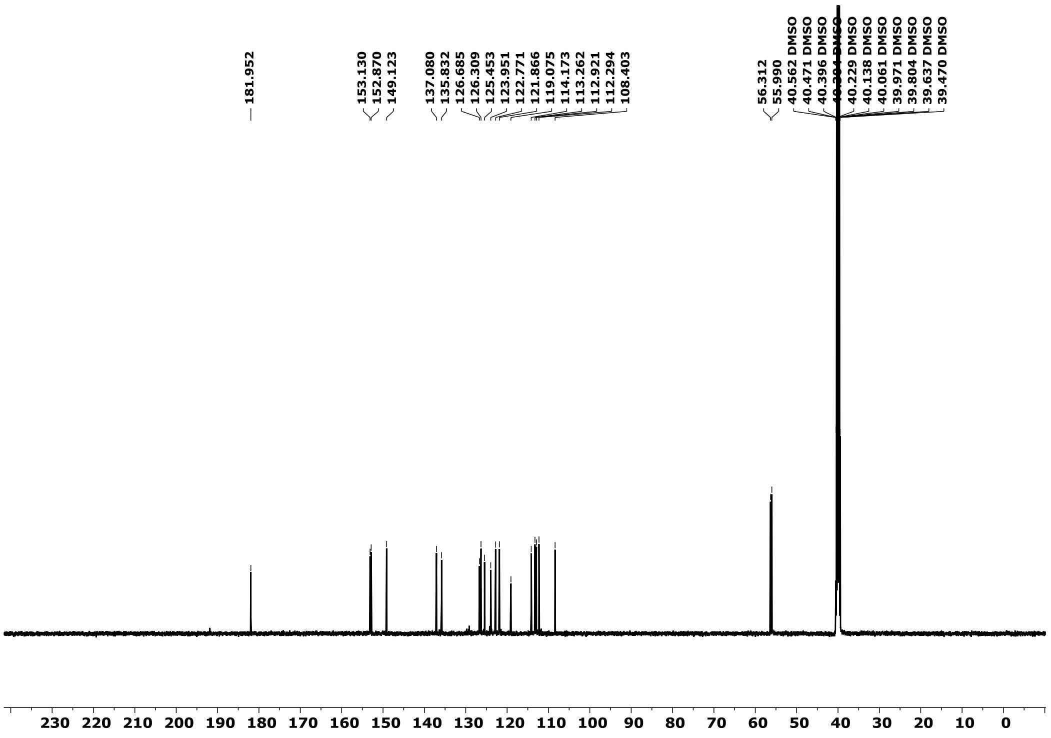

Figure S17. ^{13}C NMR (126 MHz, DMSO-d_6) spectrum of compound **8g**

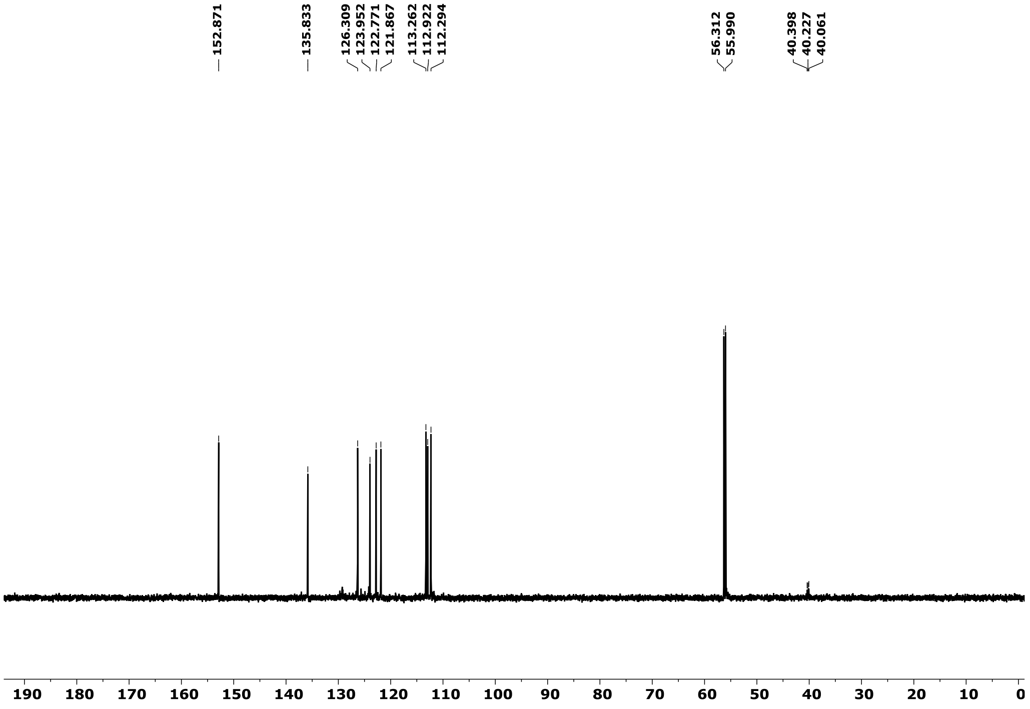

Figure S18. DEPT-135 (126 MHz, DMSO-d_6) spectrum of compound **8g**

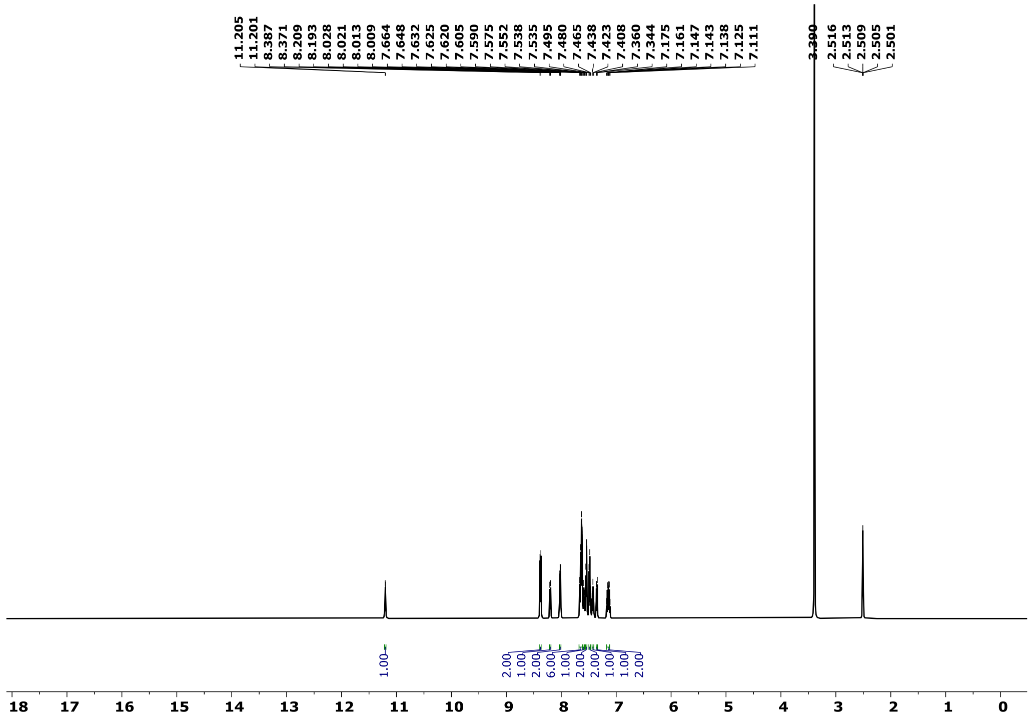

Figure S19. ^{1}H NMR (500 MHz, DMSO-d_6) spectrum of compound **10a**

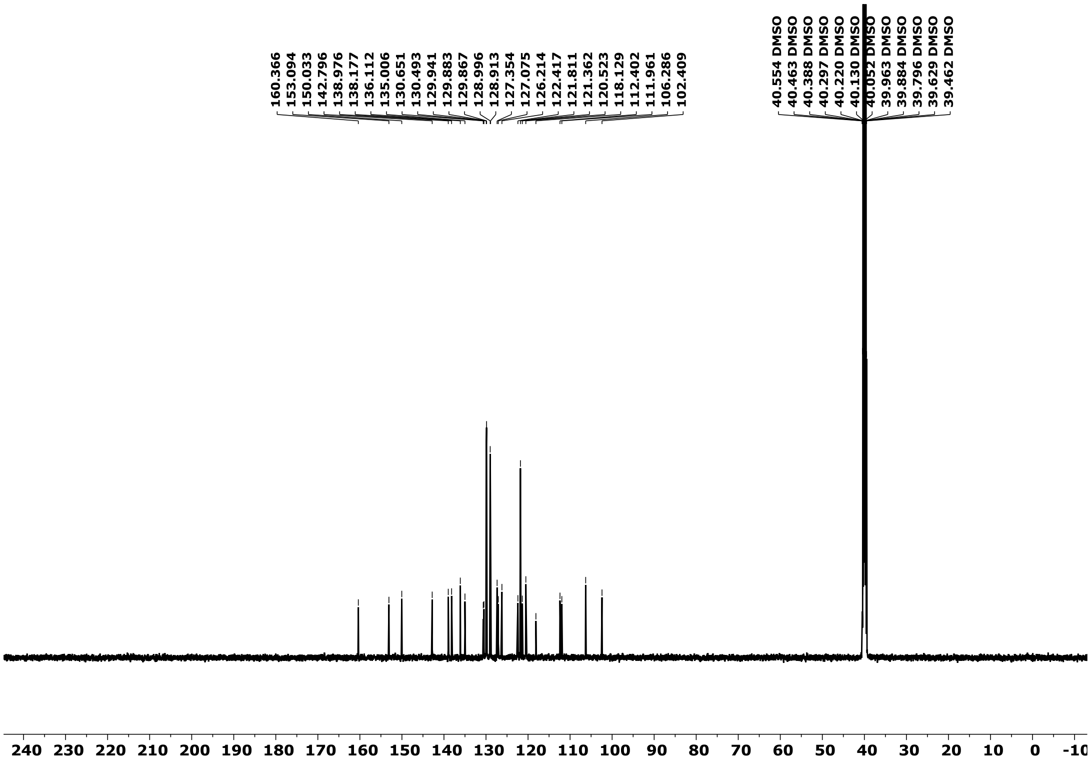

Figure S20. ^{13}C NMR (126 MHz, DMSO-d_6) spectrum of compound **10a**

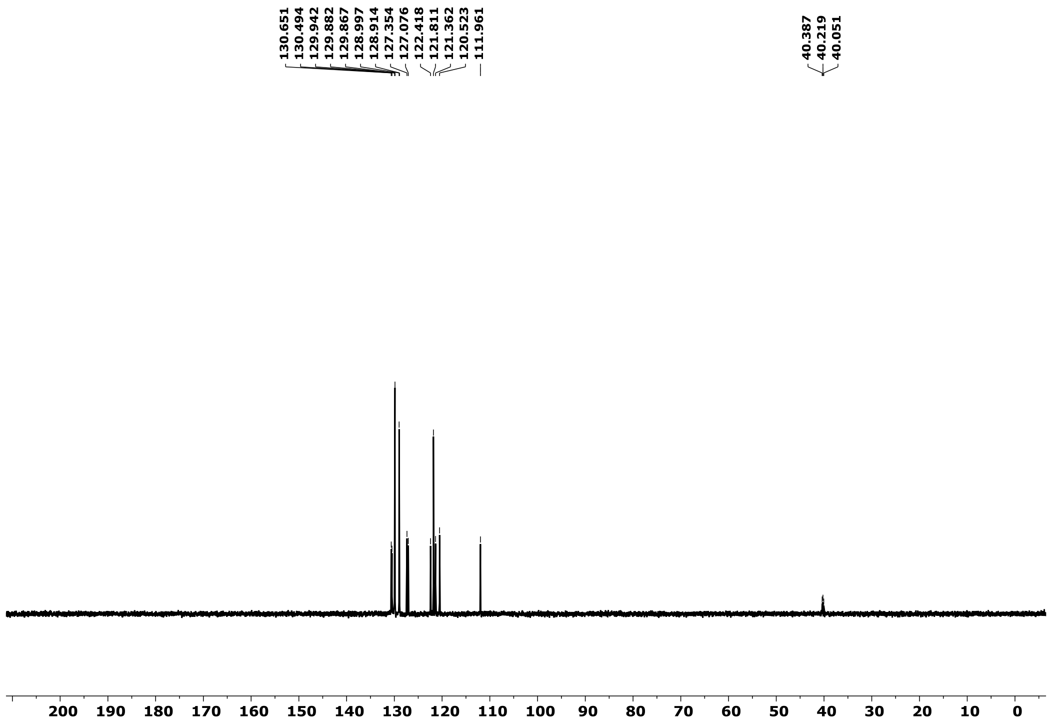

Figure S21. DEPT-135 (126 MHz, DMSO-d_6) spectrum of compound **10a**

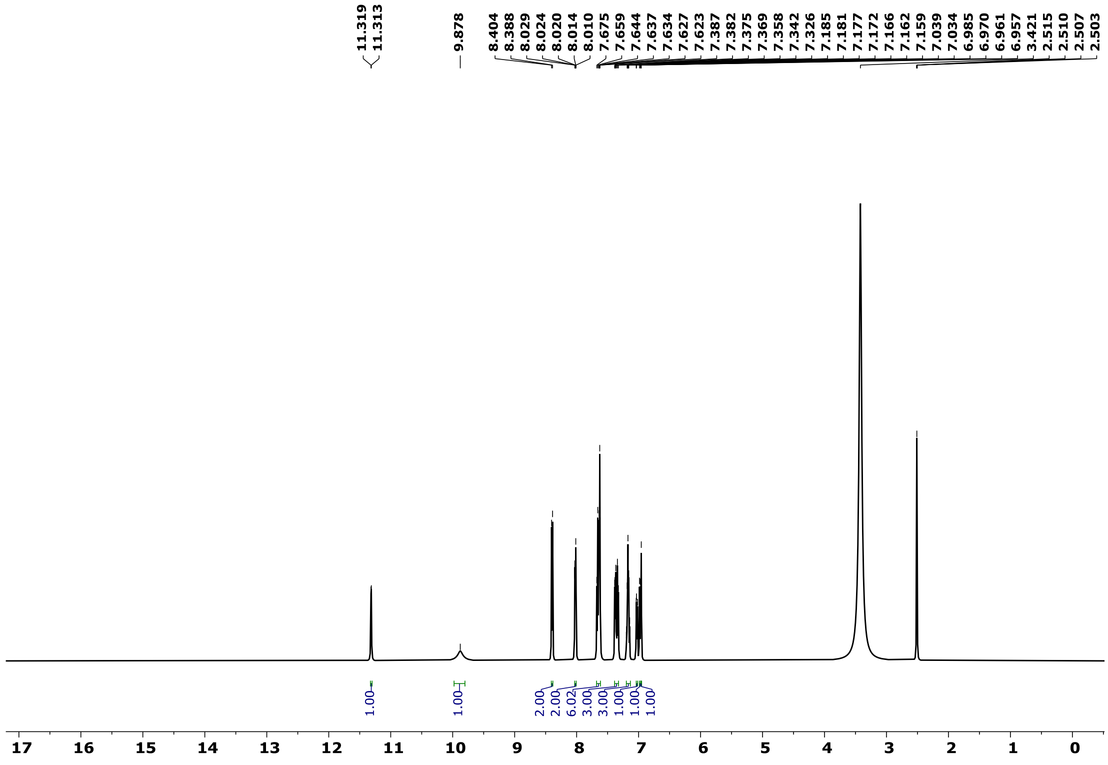

Figure S22. ^{1}H NMR (500 MHz, DMSO-d_6) spectrum of compound **10b**

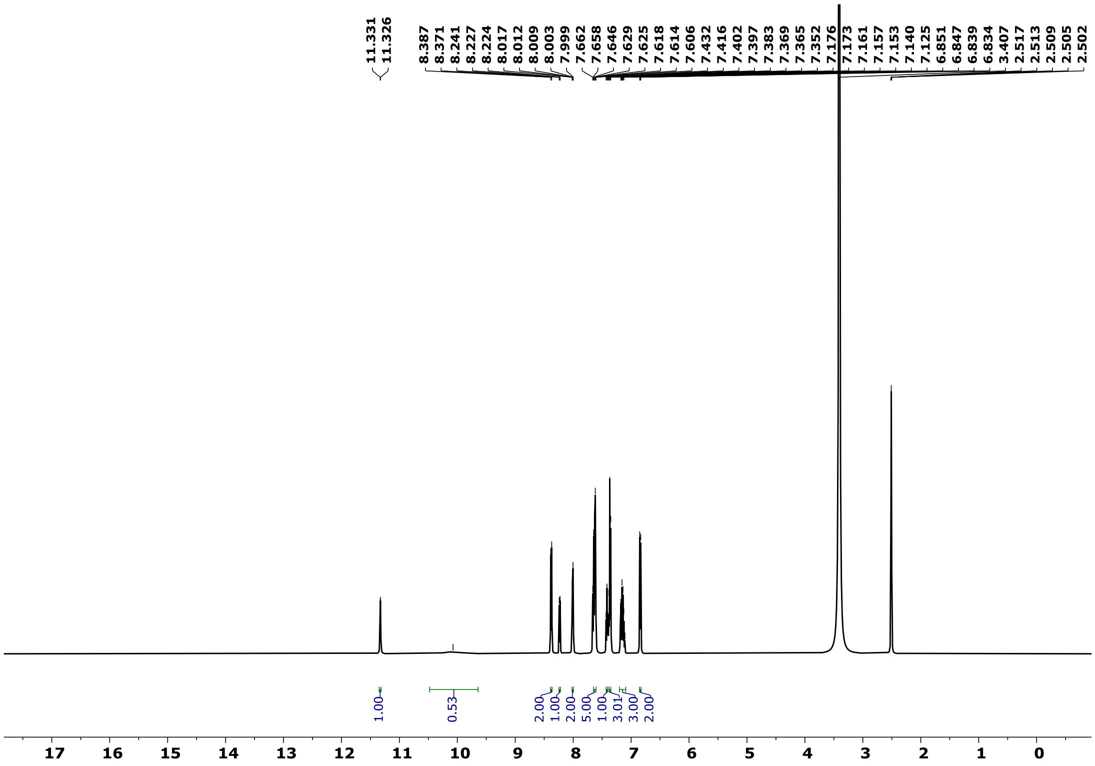

Figure S23. ^{1}H NMR (500 MHz, DMSO-d_6) spectrum of compound **10c**

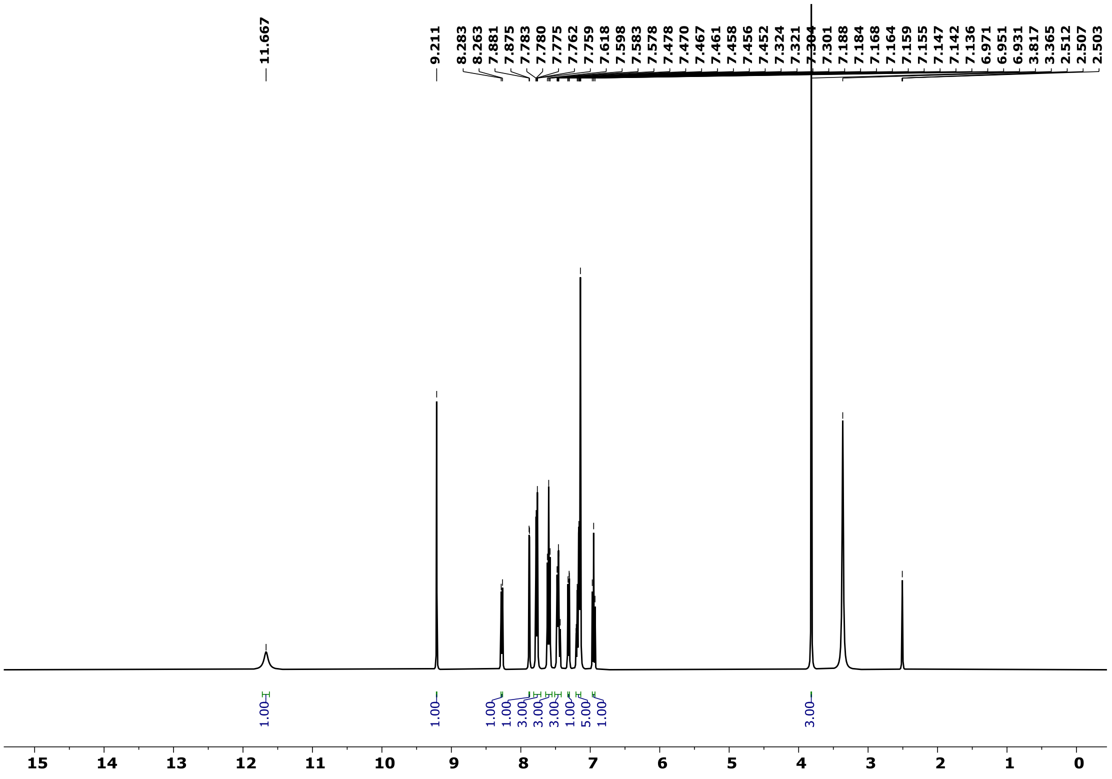

Figure S24. ^{1}H NMR (400 MHz, DMSO-d_6) spectrum of compound **10d**

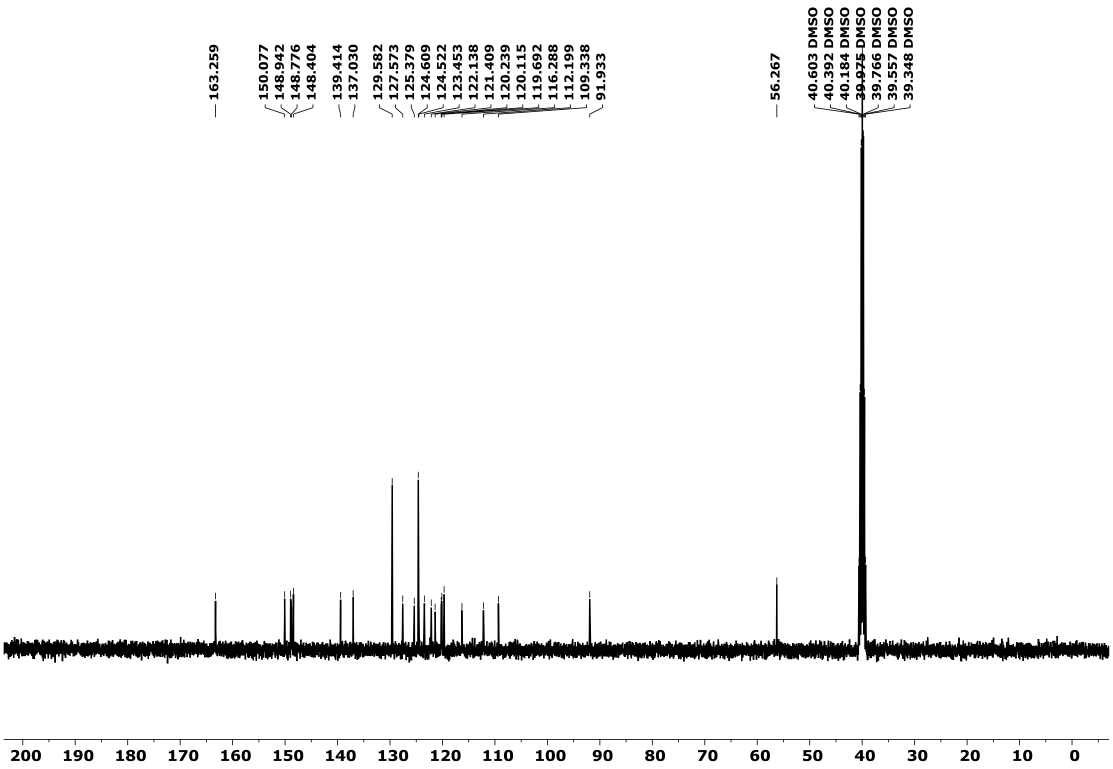

Figure S25. ^{13}C NMR (101 MHz, DMSO-d_6) spectrum of compound **10d**

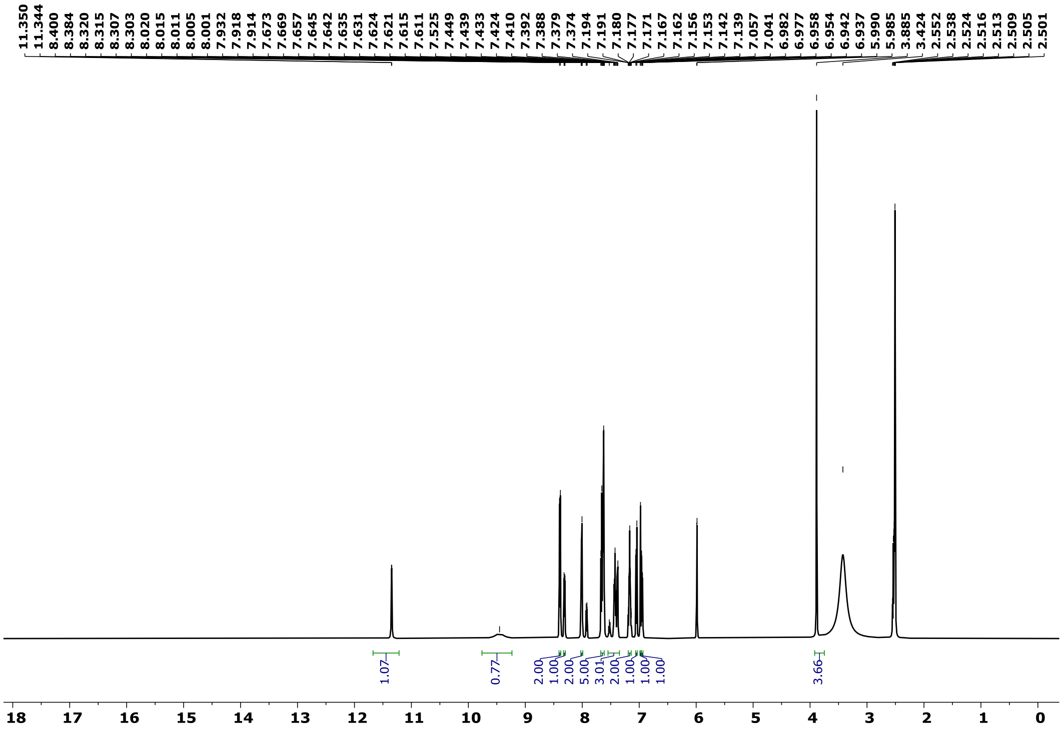

Figure S26. ^{1}H NMR (500 MHz, DMSO-d_6) spectrum of compound **10e**

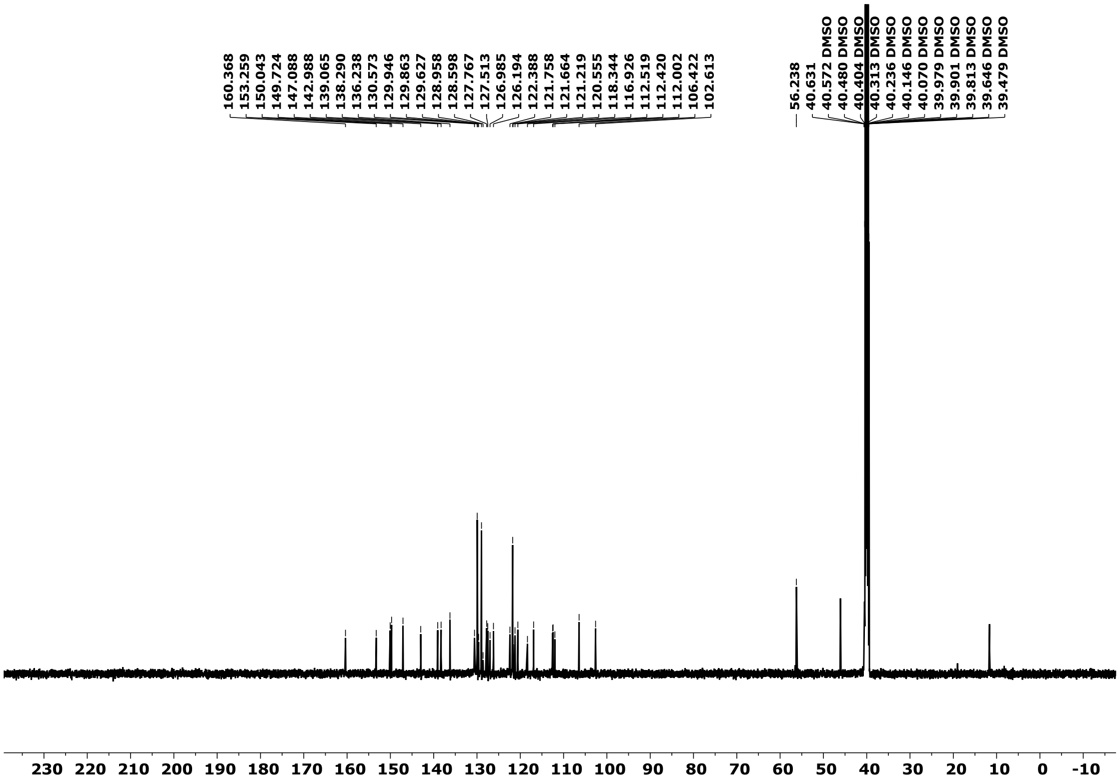

Figure S27. ^{13}C NMR (126 MHz, DMSO-d_6) spectrum of compound **10e**

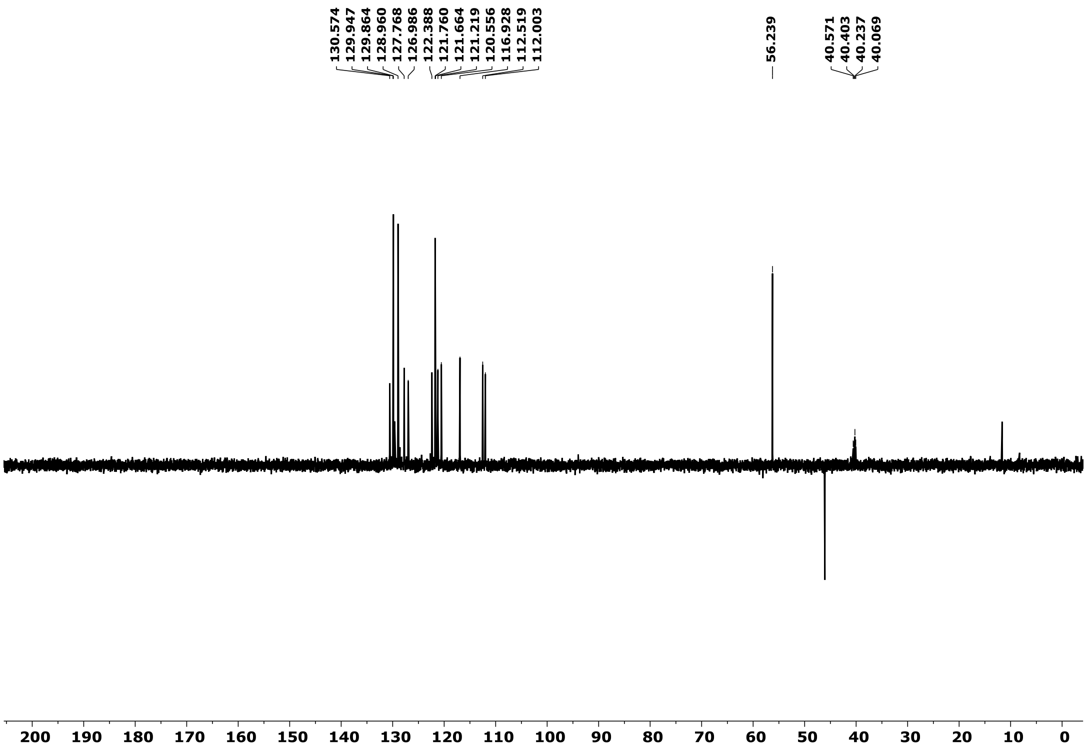

Figure S28. DEPT-135 (126 MHz, DMSO-d_6) spectrum of compound **10e**

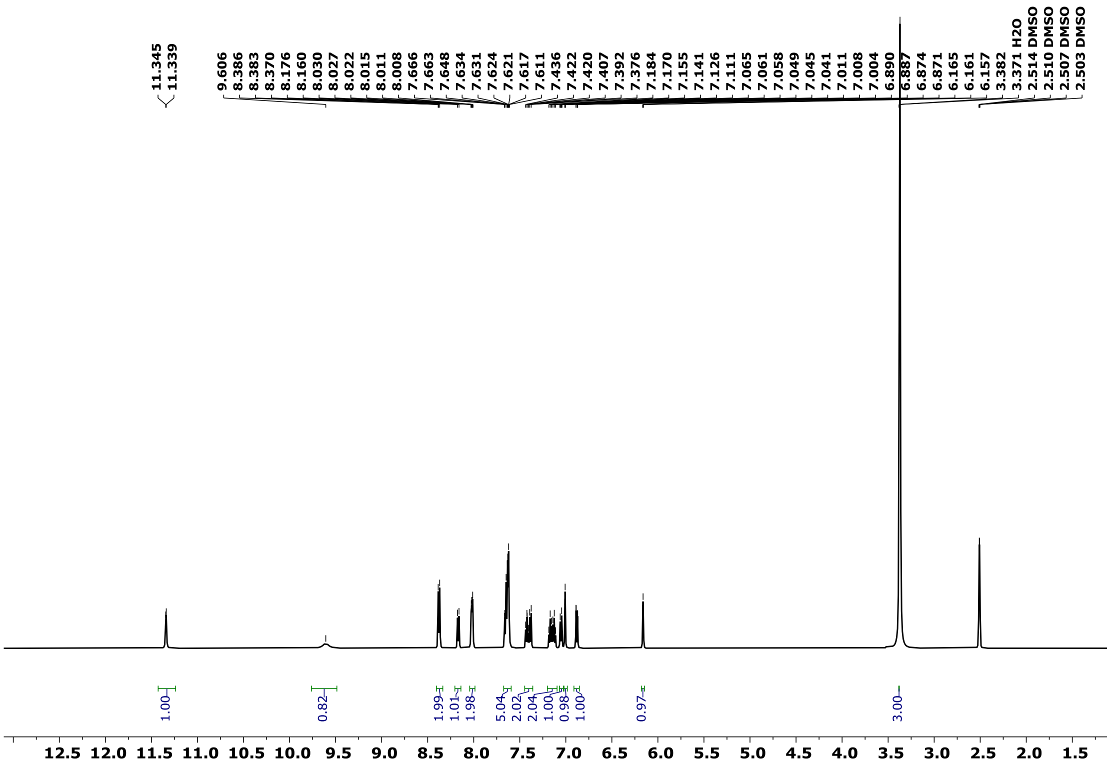

Figure S29. ^{1}H NMR (500 MHz, DMSO-d_6) spectrum of compound **10f**

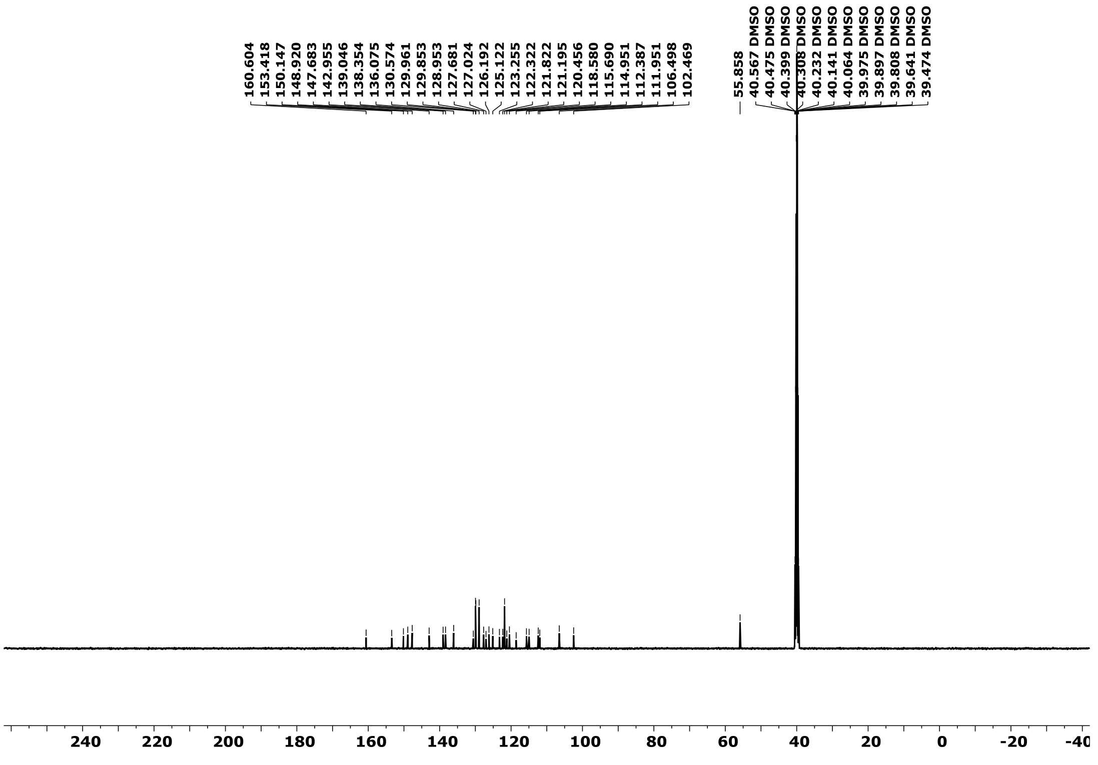

Figure S30. ^{13}C NMR (126 MHz, DMSO-d_6) spectrum of compound **10f**

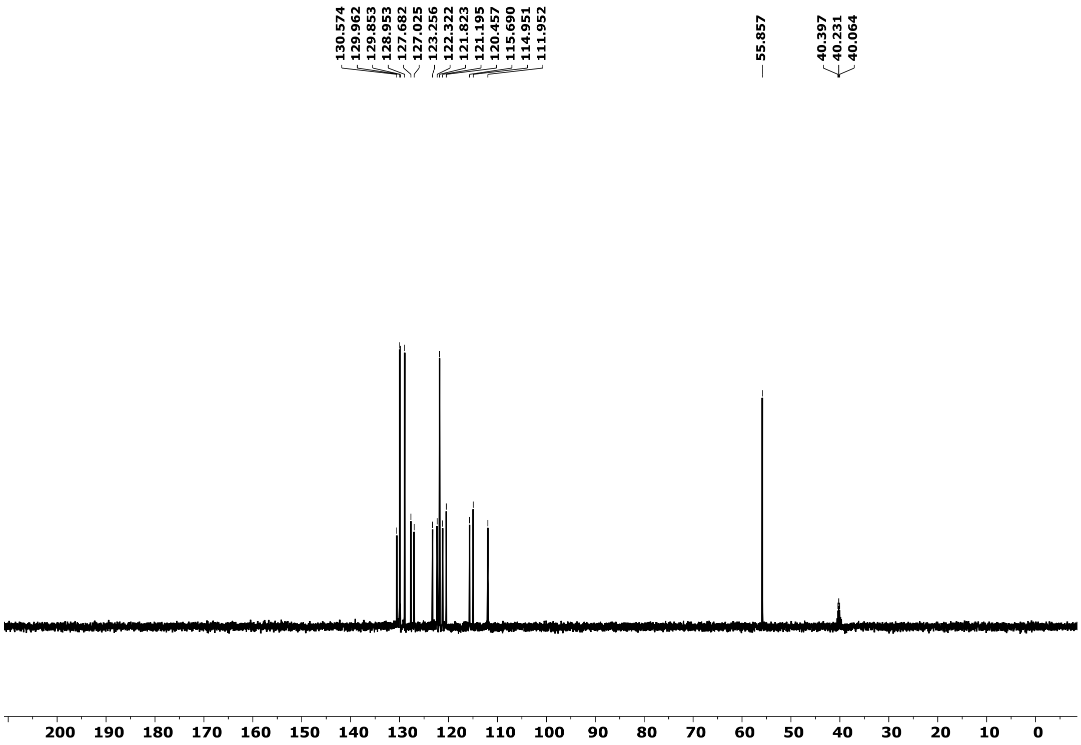

Figure S31. DEPT-135 (126 MHz, DMSO-d_6) spectrum of compound **10f**

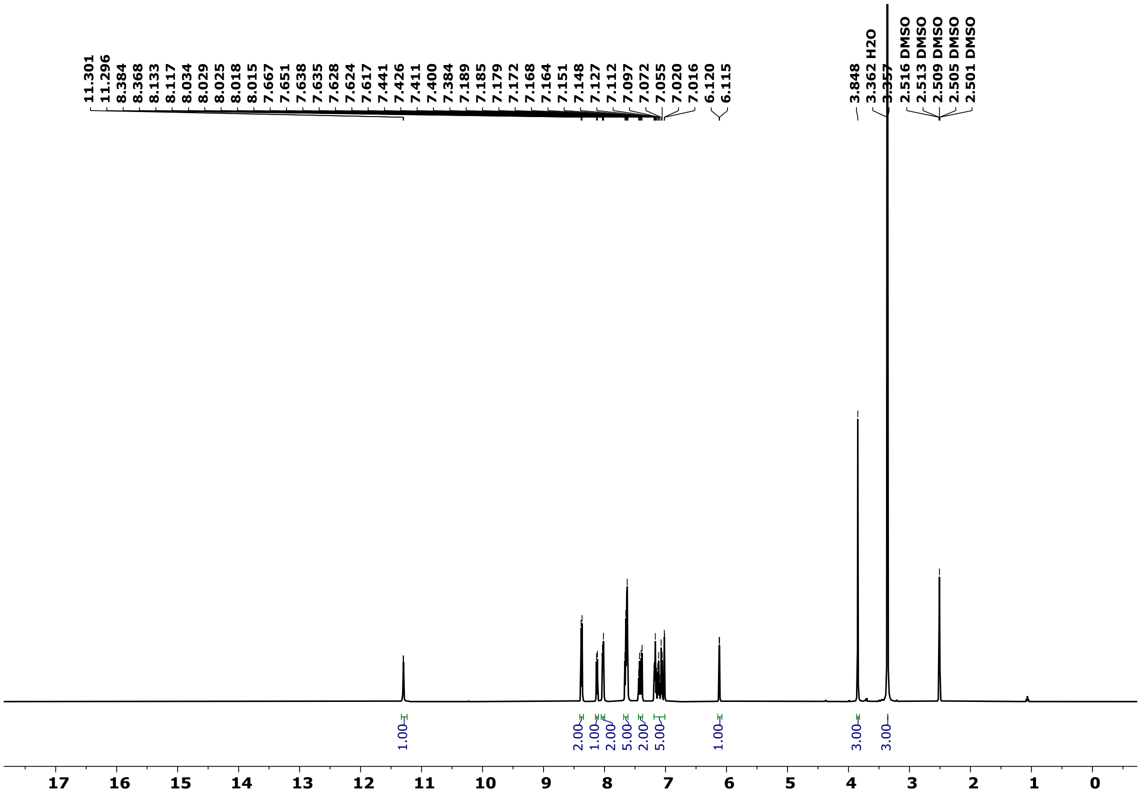

Figure S32. ^{1}H NMR (500 MHz, DMSO-d_6) spectrum of compound **10g**

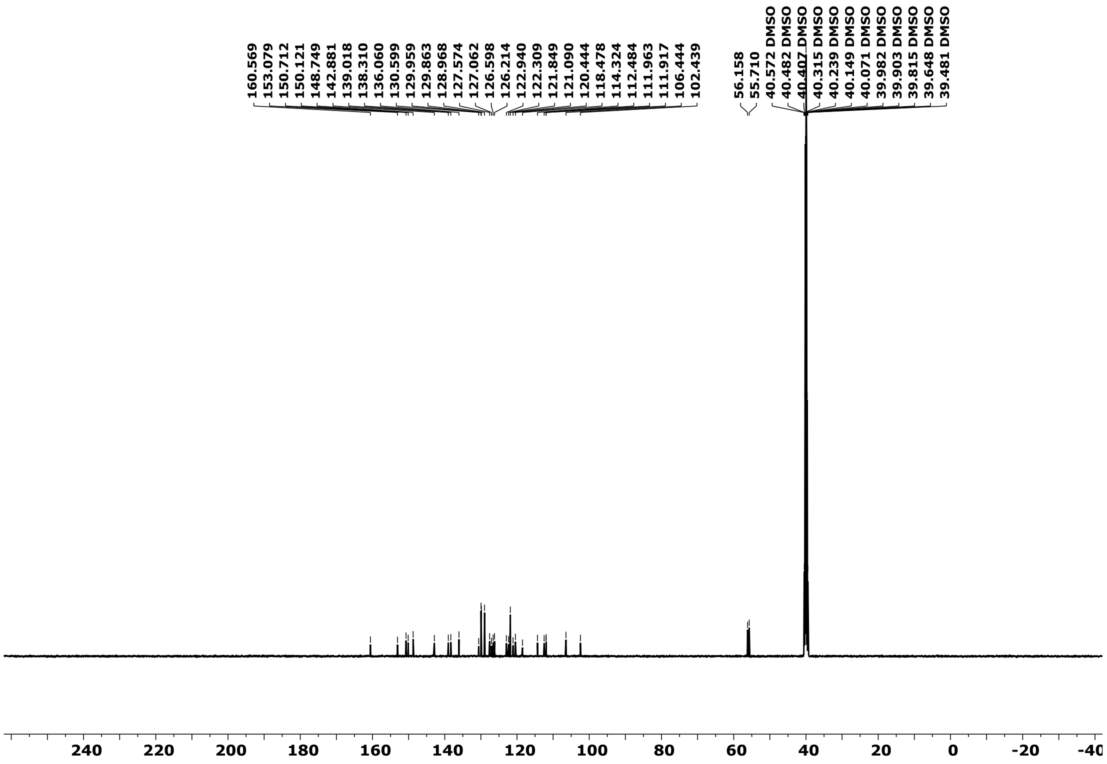

Figure S33. ^{13}C NMR (126 MHz, DMSO-d_6) spectrum of compound **10g**

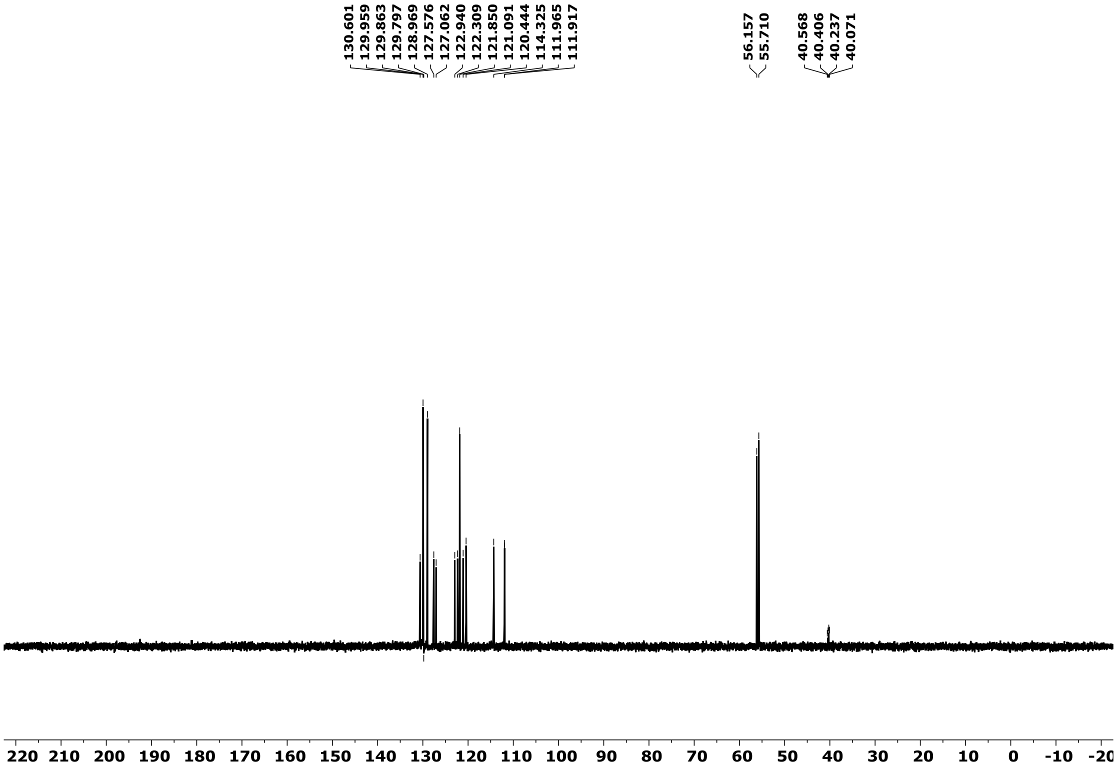

Figure S34. DEPT-135 (126 MHz, DMSO-d_6) spectrum of compound **10g**

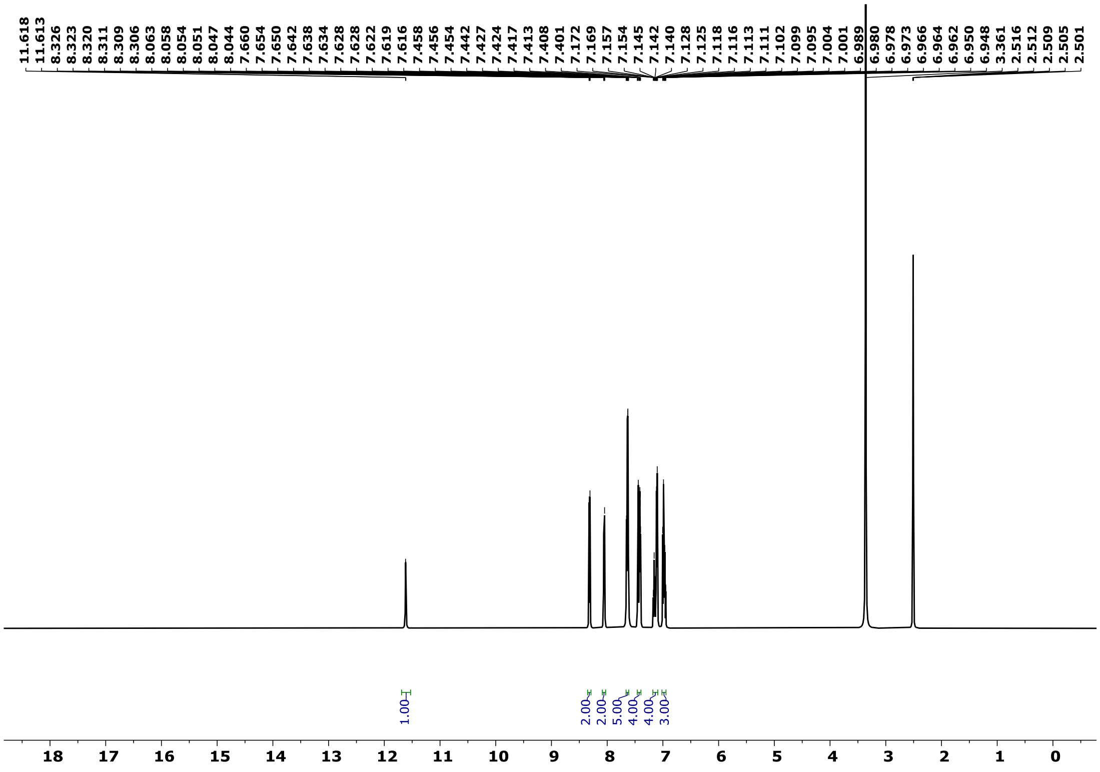

Figure S35. ^{1}H NMR (500 MHz, DMSO-d_6) spectrum of compound **12**

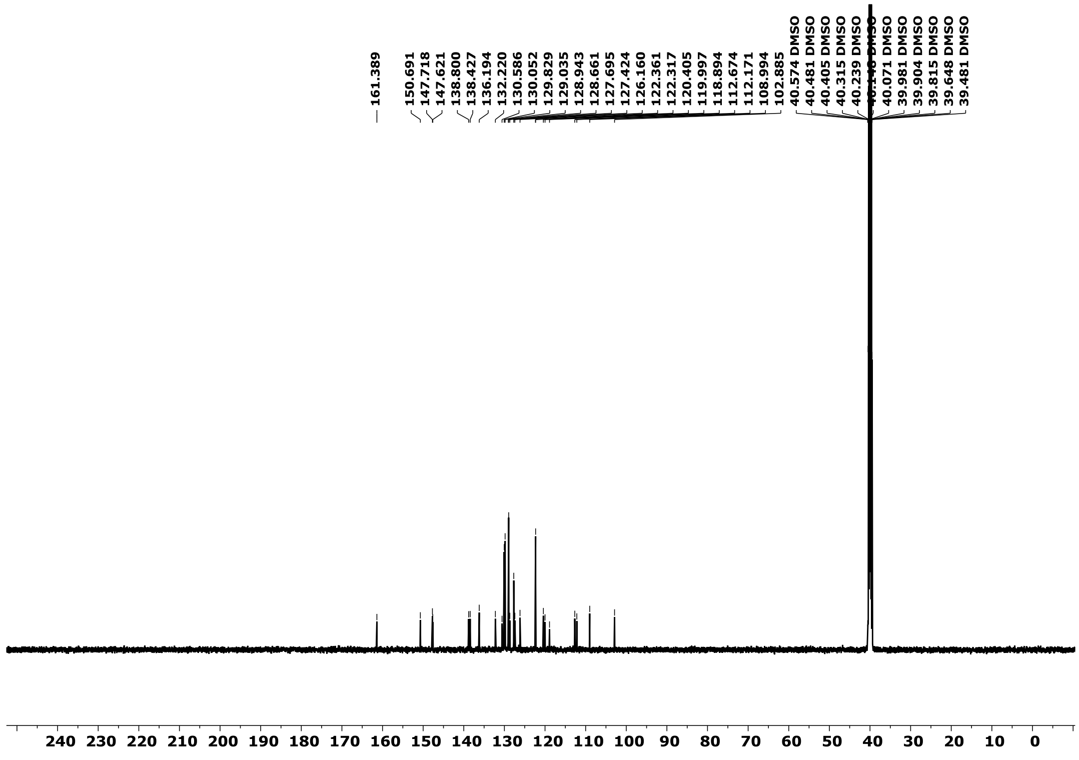

Figure S36. ^{13}C NMR (126 MHz, DMSO-d_6) spectrum of compound **12**

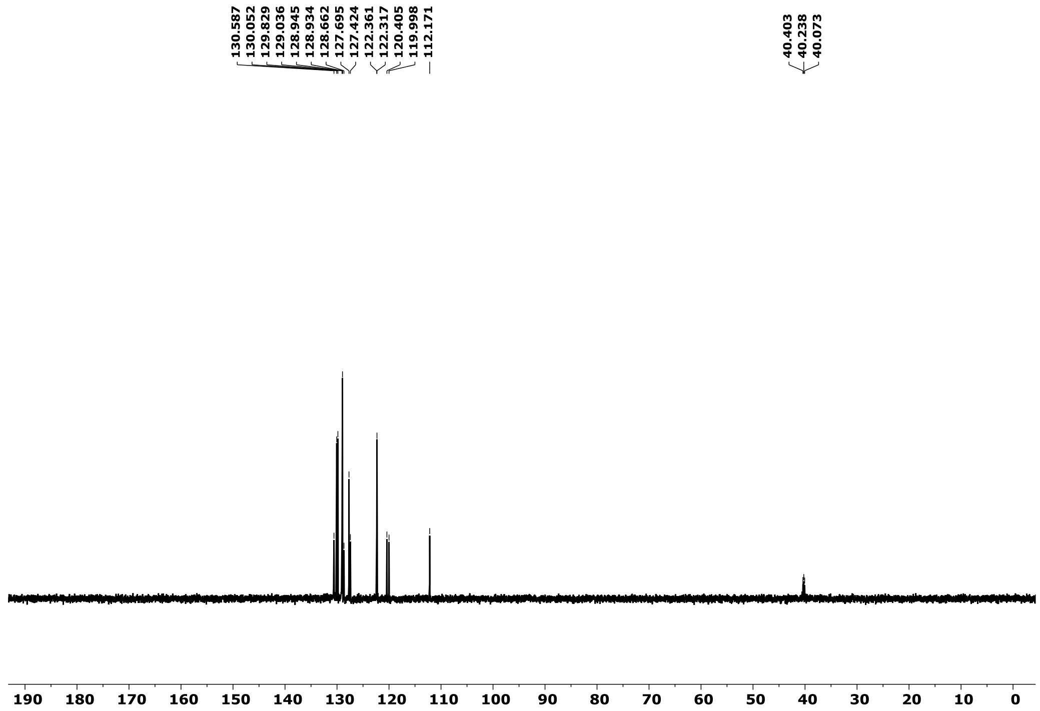

Figure S37. DEPT-135 (126 MHz, DMSO-d_6) spectrum of compound **12**

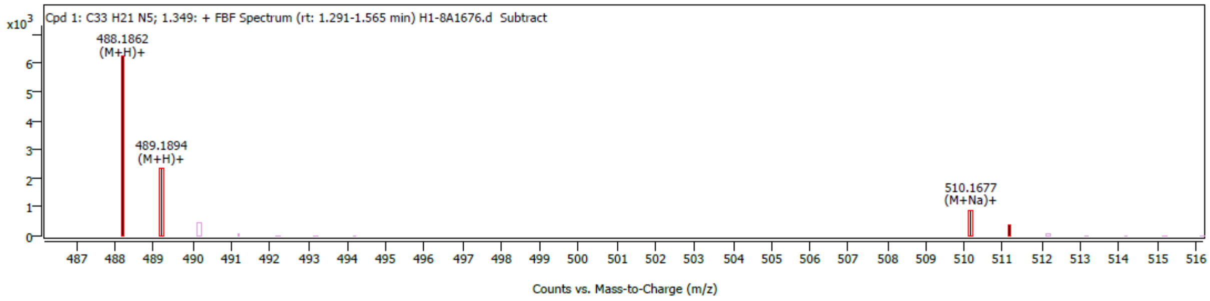

Figure S38. HRMS spectrum of compound **8a**

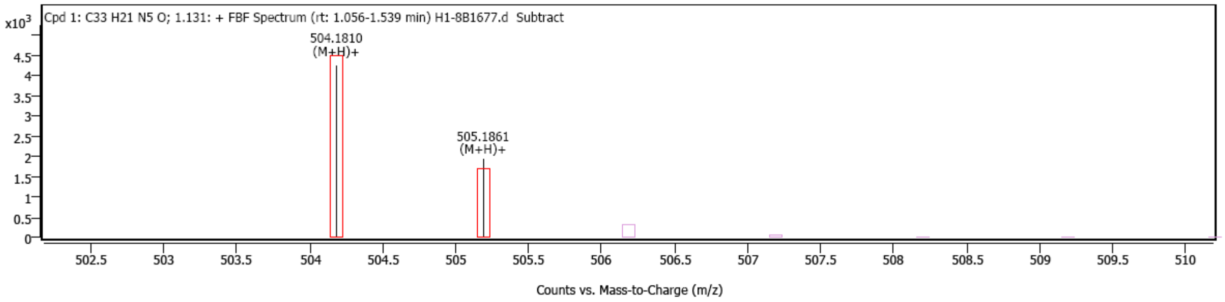

Figure S39. HRMS spectrum of compound **8b**

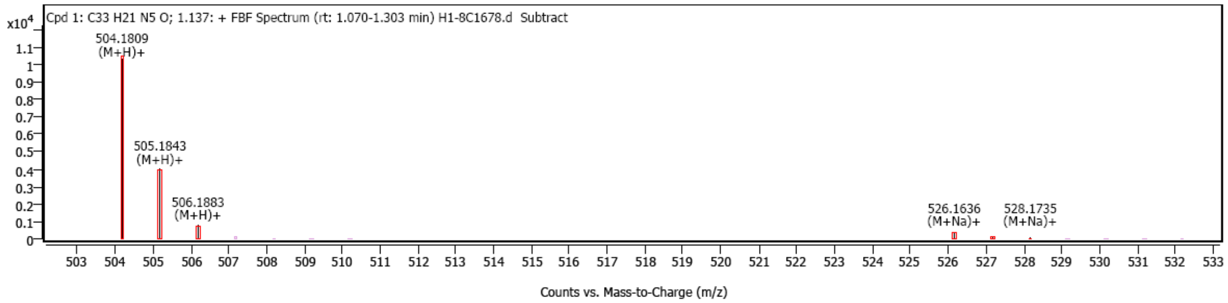

Figure S40. HRMS spectrum of compound **8c**

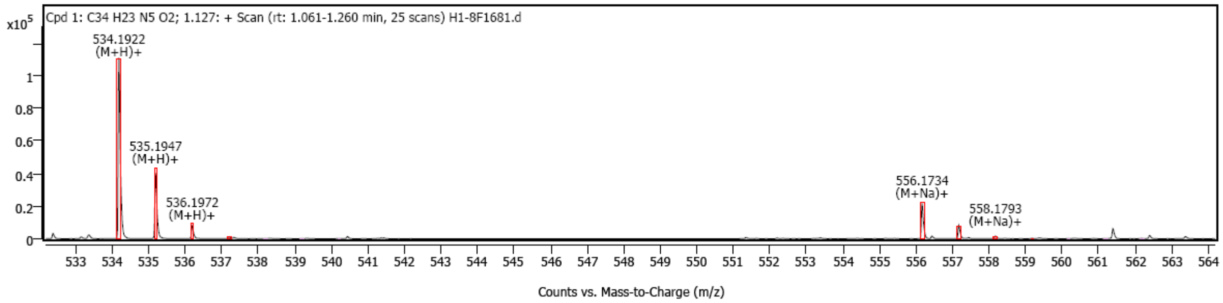

Figure S41. HRMS spectrum of compound **8e**

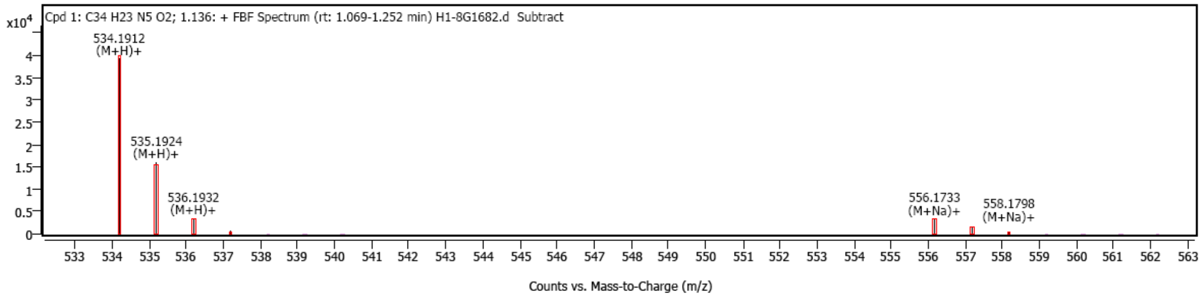

Figure S42. HRMS spectrum of compound **8f**

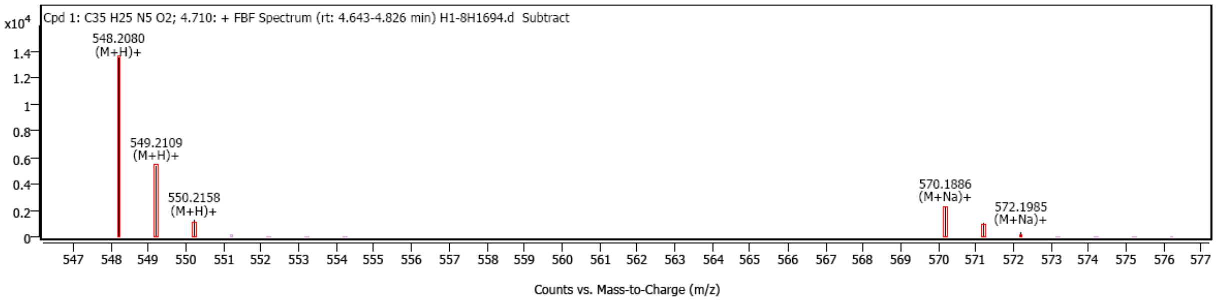

Figure S43. HRMS spectrum of compound **8g**

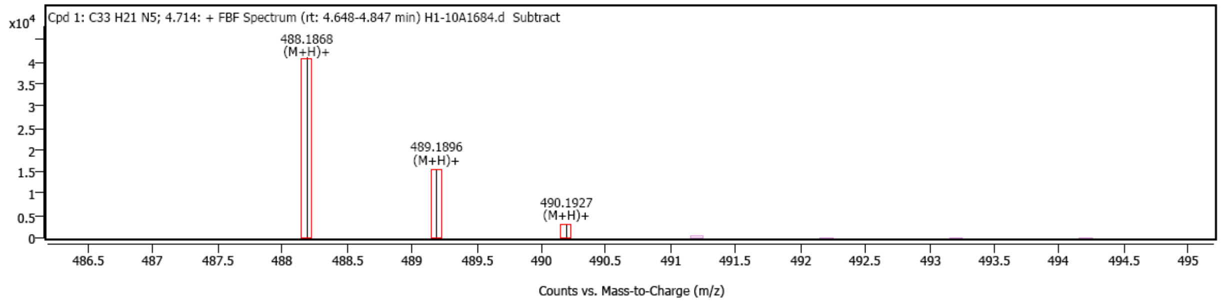

Figure S44. HRMS spectrum of compound **10a**

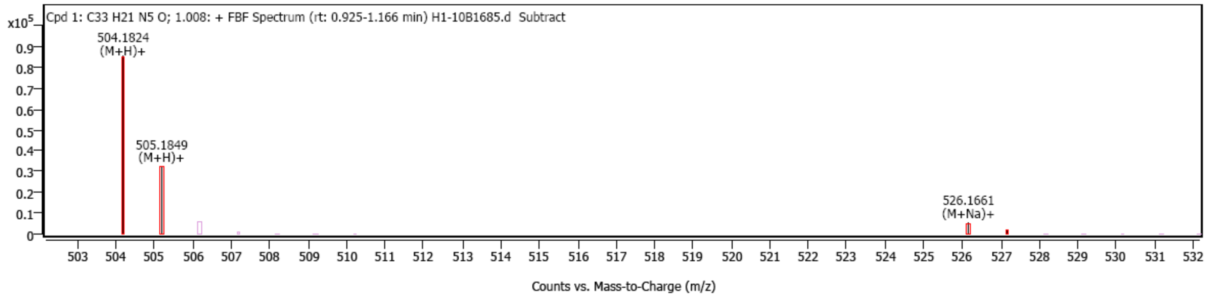

Figure S45. HRMS spectrum of compound **10b**

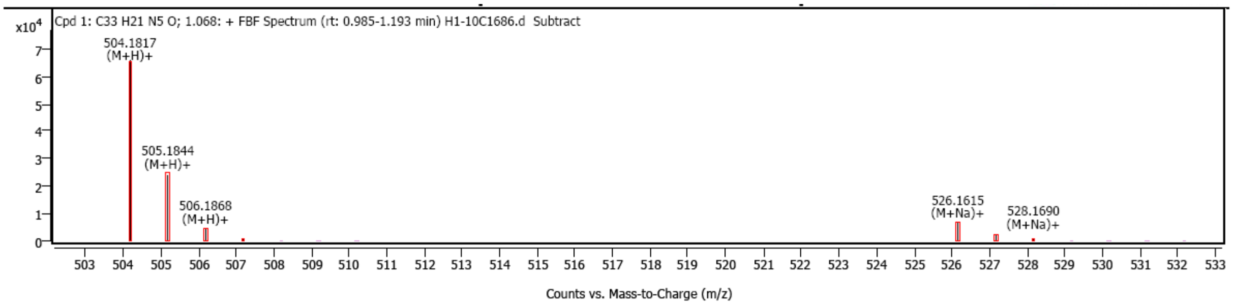

Figure S46. HRMS spectrum of compound **10c**

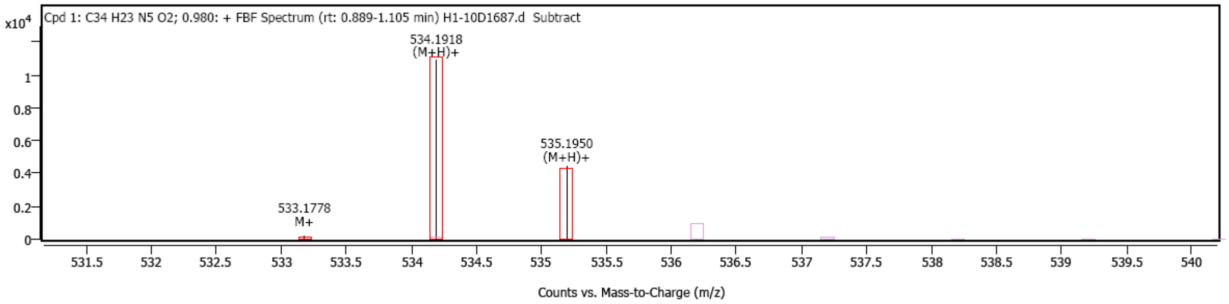

Figure S47. HRMS spectrum of compound **10d**

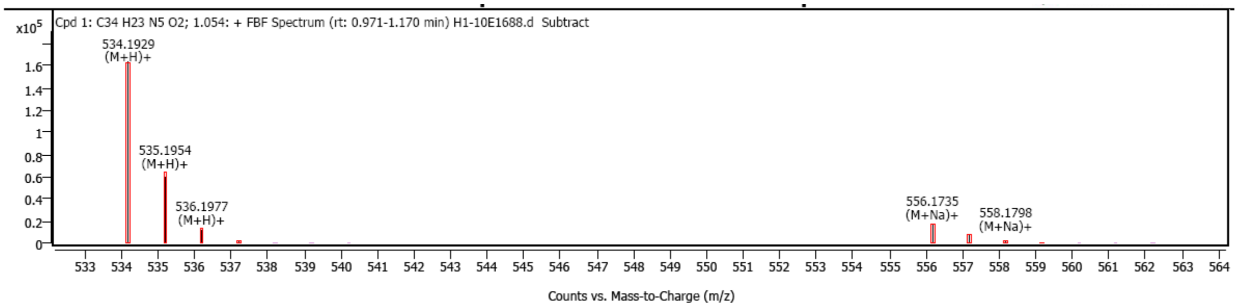

Figure S48. HRMS spectrum of compound **10e**

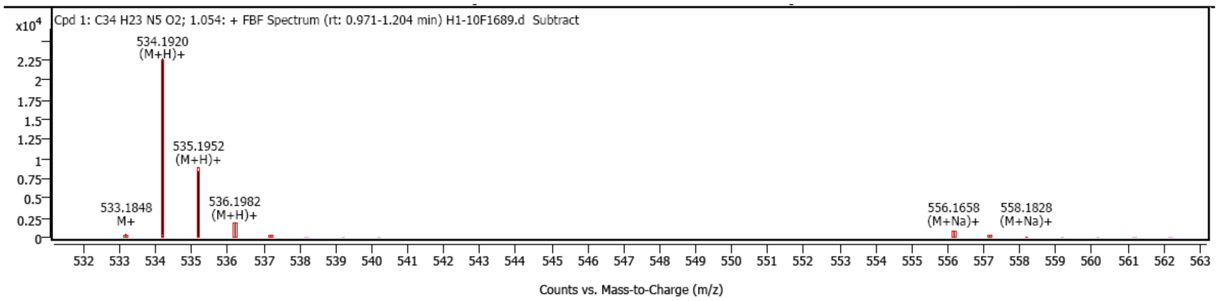

Figure S49. HRMS spectrum of compound **10f**

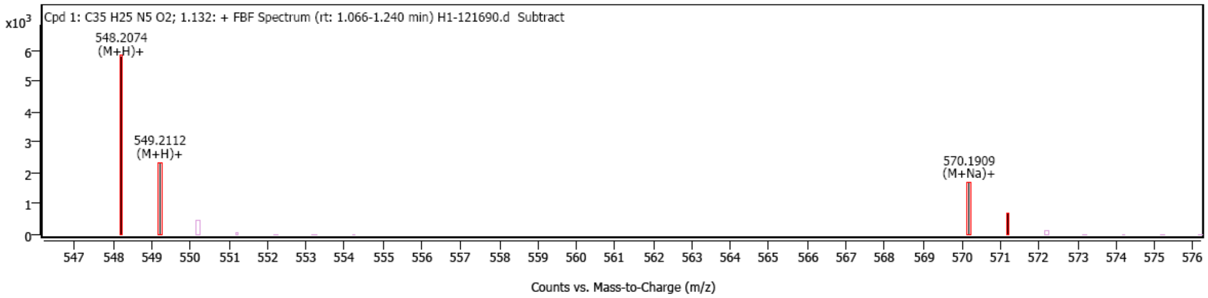

Figure S50. HRMS spectrum of compound **10g**

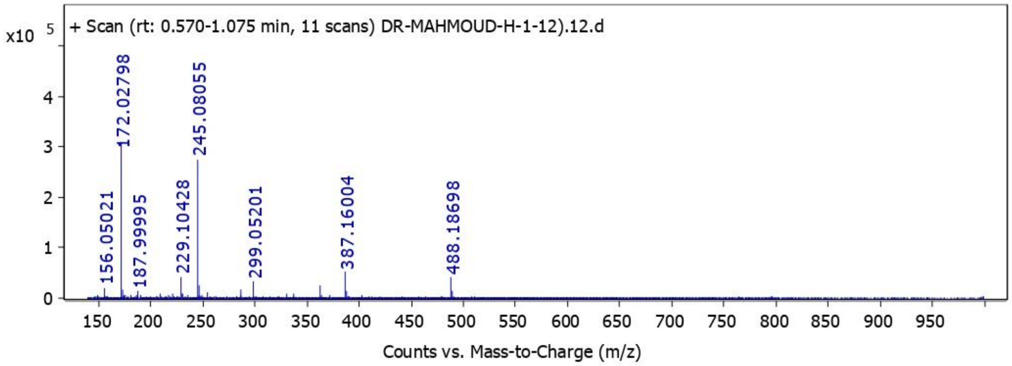

Figure S51. HRMS spectrum of compound **12**

Area Percent Report

Agilent Technologies

Data file:	C:\CHEM32\1\DATA\HAYTHAM\8A-001.D		
Sample name:	8a		
Description:	Solvent: DMSO + ACN, Flow rate: 1.5 mL/min<br>Mobile phase: 35% ACN + 65% Phosphate buffer		
Sample amount:	2.500	Sample type:	Sample
Instrument:	HPLC	Location:	Vial 1
Injection date:	4/29/2025 12:58:56AM	Injection:	1 of 1
Acq. method:	HAYTHAM.M	Injection volume:	5.000
Analysis method:	HAYTHAM.M	Acq. operator:	SYSTEM
Last changed:	4/29/2025 12:59:25AM		

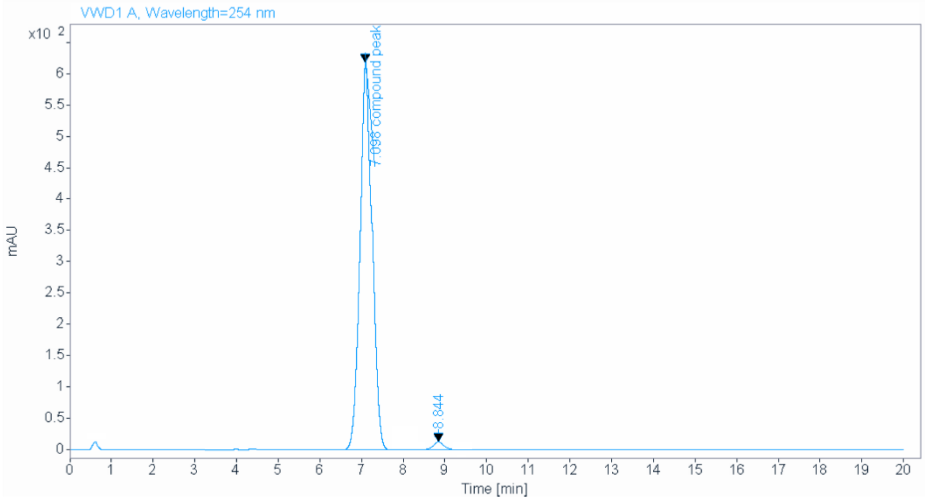

Signal: VWD1 A, Wavelength=254 nm

RT [min]	Type	Width [min]	Area	Height	Area%	Name
7.098	BV	0.3379	14213.5120	617.3170	96.0045	Compound peak
8.844	VB	0.7443	591.5216	12.6961	3.9955	
		Sum	14805.0336			

Figure S52. HPLC spectrum of compound **8a**

Area Percent Report

Agilent Technologies

Data file:	C:\CHEM32\1\DATA\HAYTHAM\8B-002.D		
Sample name:	8b		
Description:	Solvent: DMSO + ACN, Flow rate: 1.5 mL/min<br>Mobile phase: 35% ACN + 65% Phosphate buffer		
Sample amount:	2.500	Sample type:	Sample
Instrument:	HPLC	Location:	Vial 2
Injection date:	4/29/2025 4:05:48 AM	Injection:	1 of 1
Acq. method:	HAYTHAM.M	Injection volume:	5.000
Analysis method:	HAYTHAM.M	Acq. operator:	SYSTEM
Last changed:	4/29/2025 4:20:10 AM<br>(modified after loading)		

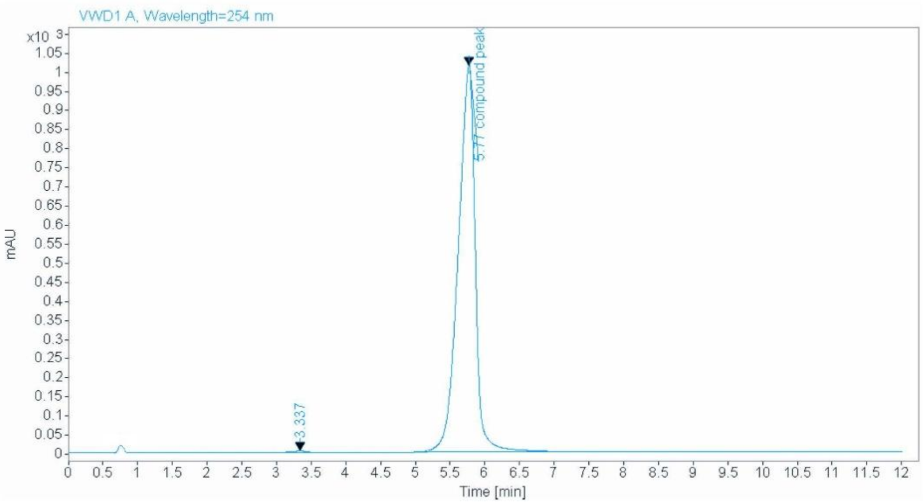

Signal: VWD1 A, Wavelength=254 nm

RT [min]	Type	Width [min]	Area	Height	Area%	Name
3.337	BV	0.2564	101.1603	5.3740	0.5927	
5.770	BB	0.2646	16967.1275	1011.5413	99.4073	Compound peak
		Sum	17068.2878			

Figure S53. HPLC spectrum of compound **8b**

Area Percent Report

Agilent Technologies

Data file:	C:\CHEM32\1\DATA\HAYTHAM\8C-007.D		
Sample name:	8c		
Description:	Solvent: DMSO + ACN, Flow rate: 1.5 mL/min<br>Mobile phase: 40% ACN + 60% Phosphate buffer		
Sample amount:	2.500	Sample type:	Sample
Instrument:	HPLC	Location:	Vial 1
Injection date:	4/29/2025 12:20:32 AM	Injection:	1 of 1
Acq. method:	HAYTHAM.M	Injection volume:	5.000
Analysis method:	HAYTHAM.M	Acq. operator:	SYSTEM
Last changed:	4/29/2025 12:33:10 AM<br>(modified after loading)		

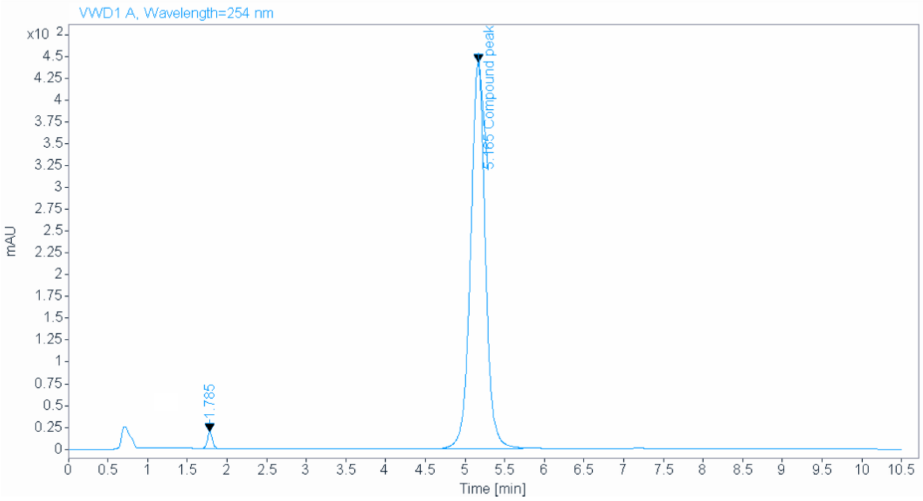

Signal: VWD1 A, Wavelength=254 nm

RT [min]	Type	Width [min]	Area	Height	Area%	Name
1.785	BV	0.0676	85.5028	19.3174	1.4683	
5.165	BV	0.1993	5737.7798	442.4784	98.5317	Compound peak
		Sum	5823.2826			

Figure S54. HPLC spectrum of compound **8c**

Area Percent Report

Agilent Technologies

Data file:	C:\CHEM32\1\DATA\HAYTHAM\8D-003.D		
Sample name:	8d		
Description:	Solvent: DMSO + ACN, Flow rate: 1.5 mL/min<br>Mobile phase: 35% ACN + 65% Phosphate buffer		
Sample amount:	2.500	Sample type:	Sample
Instrument:	HPLC	Location:	Vial 4
Injection date:	4/29/2025 2:14:26 AM	Injection:	1 of 1
Acq. method:	HAYTHAM.M	Injection volume:	5.000
Analysis method:	HAYTHAM.M	Acq. operator:	SYSTEM
Last changed:	4/29/2025 4:30:42 AM<br>(modified after loading)		

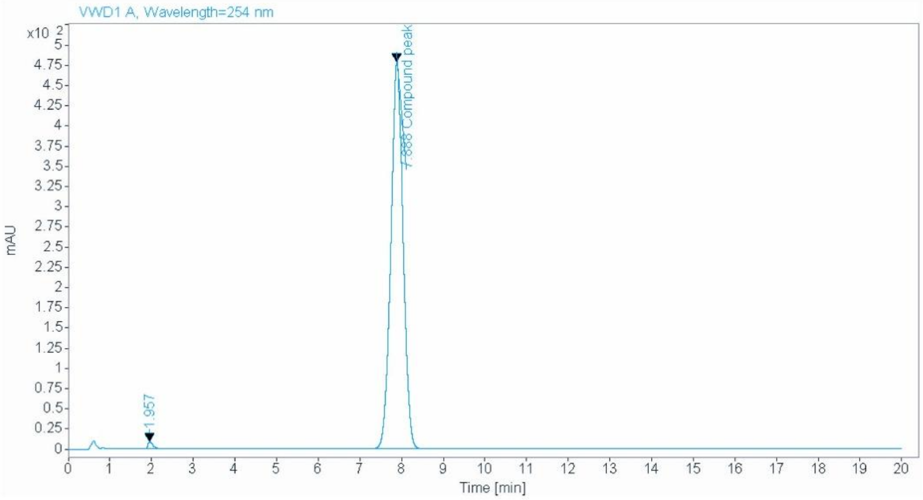

Signal: VWD1 A, Wavelength=254 nm

RT [min]	Type	Width [min]	Area	Height	Area%	Name
1.957	BV	0.1360	75.8321	8.7987	0.5808	
7.888	BV	0.3774	12981.9246	478.9035	99.4192	Compound peak
		Sum	13057.7567			

Figure S55. HPLC spectrum of compound **8d**

Area Percent Report

Agilent Technologies

Data file:	C:\CHEM32\1\DATA\HAYTHAM\8E-004.D		
Sample name:	8e		
Description:	Solvent: DMSO + ACN, Flow rate: 1.5 mL/min<br>Mobile phase: 35% ACN + 65% Phosphate buffer		
Sample amount:	2.500	Sample type:	Sample
Instrument:	HPLC	Location:	Vial 5
Injection date:	4/29/2025 4:20:30 AM	Injection:	1 of 1
Acq. method:	HAYTHAM.M	Injection volume:	5.000
Analysis method:	HAYTHAM.M	Acq. operator:	SYSTEM
Last changed:	4/29/2025 4:32:34 AM<br>(modified after loading)		

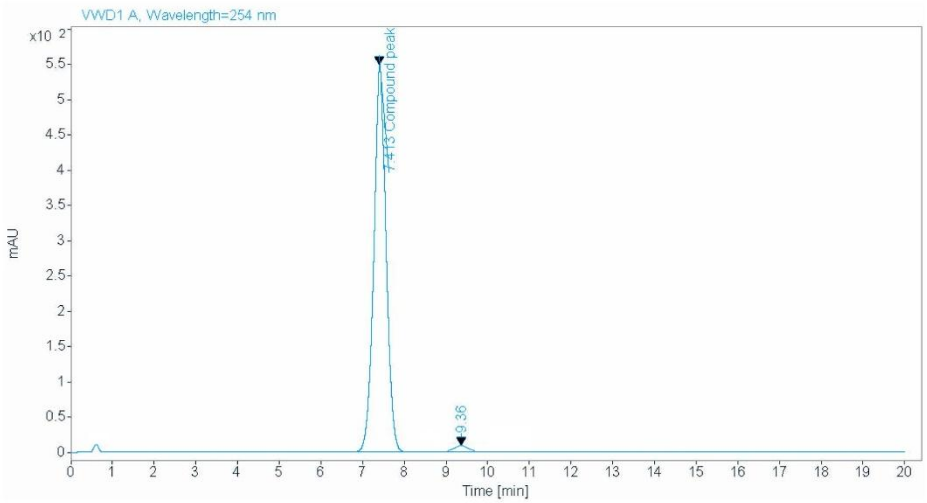

Signal: VWD1 A, Wavelength=254 nm

RT [min]	Type	Width [min]	Area	Height	Area%	Name
7.413	BV	0.3590	13612.8221	548.8845	96.4440	Compound peak
9.360	VV	0.9061	501.9150	9.2525	3.5560	
		Sum	14114.7371			

Figure S56. HPLC spectrum of compound **8e**

Area Percent Report

Agilent Technologies

**Data file:**	C:\CHEM32\1\DATA\HAYTHAM\8F-003.D		
**Sample name:**	8f		
**Description:**	Solvent: DMSO + ACN<br>Mobile phase: 35% ACN + 65% Phosphate buffer		
**Sample amount:**	2.500	**Sample type:**	Sample
**Instrument:**	HPLC	**Location:**	Vial 6
**Injection date:**	4/29/2025 4:09:03 AM	**Injection:**	1 of 1
**Acq. method:**	HAYTHAM.M	**Injection volume:**	5.000
**Analysis method:**	HAYTHAM.M	**Acq. operator:**	SYSTEM
**Last changed:**	4/29/2025 4:22:34 AM<br>(modified after loading)		

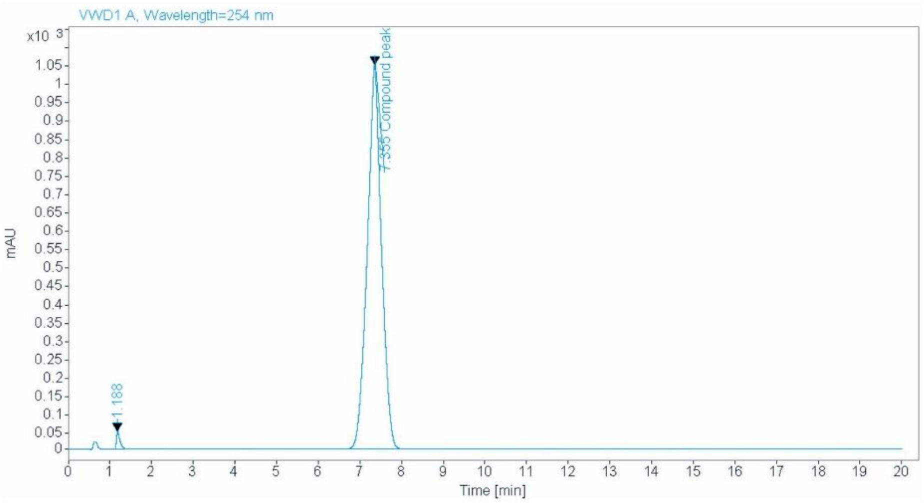

Signal: VWD1 A, Wavelength=254 nm

RT [min]	Type	Width [min]	Area	Height	Area%	Name
1.188	BV	0.1294	521.1734	56.0039	1.4424	
7.355	BB	0.4210	35612.2109	1046.2883	98.5576	Compound peak
		Sum	36133.3843			

Figure S57. HPLC spectrum of compound **8f**

Area Percent Report

Agilent Technologies

**Data file:**	C:\CHEM32\1\DATA\HAYTHAM\8G-003.D		
**Sample name:**	8g		
**Description:**	Solvent: DMSO + ACN, Flow rate: 1.5 mL/min<br>Mobile phase: 35% ACN + 65% Phosphate buffer		
**Sample amount:**	2.500	**Sample type:**	Sample
**Instrument:**	HPLC	**Location:**	Vial 7
**Injection date:**	4/29/2025 4:41:50 AM	**Injection:**	1 of 1
**Acq. method:**	HAYTHAM.M	**Injection volume:**	5.000
**Analysis method:**	HAYTHAM.M	**Acq. operator:**	SYSTEM
**Last changed:**	4/29/2025 4:47:17 AM<br>(modified after loading)		

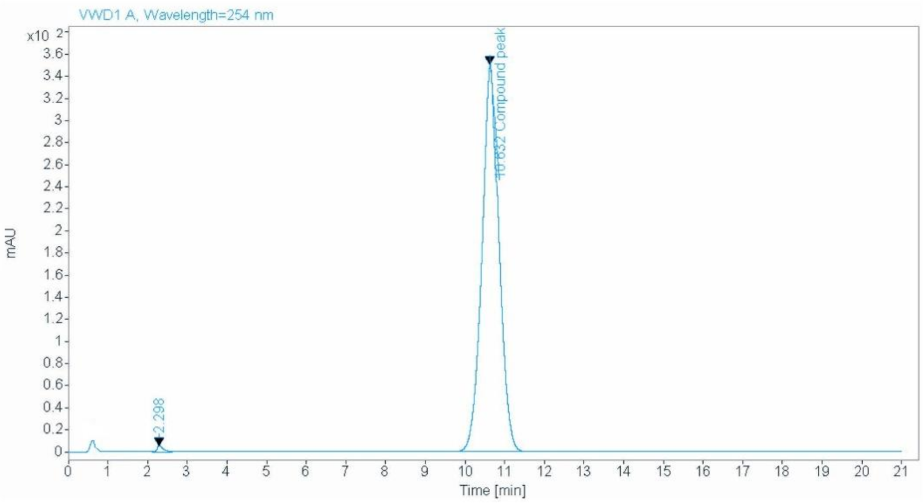

Signal: VWD1 A, Wavelength=254 nm

RT [min]	Type	Width [min]	Area	Height	Area%	Name
2.298	VV	0.1846	62.1824	5.2683	0.4425	
10.632	BV	0.5386	13991.1523	350.2096	99.5575	Compound peak
		Sum	14053.3347			

Figure S58. HPLC spectrum of compound **8g**

Area Percent Report

Agilent Technologies

Data file:	C:\CHEM32\1\DATA\HAYTHAM\10A-003.D		
Sample name:	10a		
Description:	Solvent: DMSO + ACN<br>Mobile phase: 35% ACN + 65% Phosphate buffer		
Sample amount:	2.500	Sample type:	Sample
Instrument:	HPLC	Location:	Vial 8
Injection date:	4/29/2025 3:16:21 AM	Injection:	1 of 1
Acq. method:	HAYTHAM.M	Injection volume:	5.000
Analysis method:	HAYTHAM.M	Acq. operator:	SYSTEM
Last changed:	4/29/2025 4:25:22 AM<br>(modified after loading)		

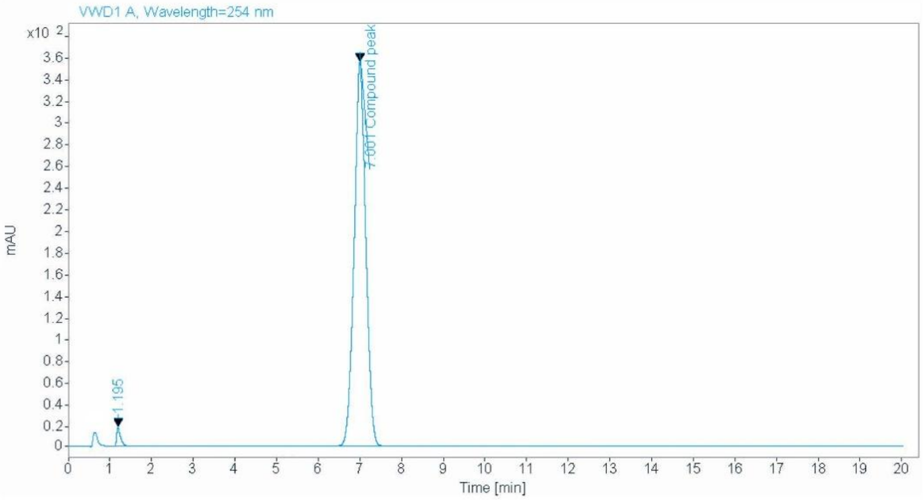

Signal: VWD1 A, Wavelength=254 nm

RT [min]	Type	Width [min]	Area	Height	Area%	Name
1.195	BV	0.1220	161.4325	19.5307	1.7251	
7.001	VB	0.3691	9196.6911	354.3398	98.2749	Compound peak
		Sum	9358.1236			

Figure S59. HPLC spectrum of compound **10a**

Area Percent Report

Agilent Technologies

Data file:	C:\CHEM32\1\DATA\HAYTHAM\10B-001.D		
Sample name:	10b		
Description:	Solvent: DMSO + ACN<br>Mobile phase: 35% ACN + 65% Phosphate buffer		
Sample amount:	2.500	Sample type:	Sample
Instrument:	HPLC	Location:	Vial 9
Injection date:	4/29/2025 2:55:03 AM	Injection:	1 of 1
Acq. method:	HAYTHAM.M	Injection volume:	5.000
Analysis method:	HAYTHAM.M	Acq. operator:	SYSTEM
Last changed:	4/29/2025 4:23:25 AM<br>(modified after loading)		

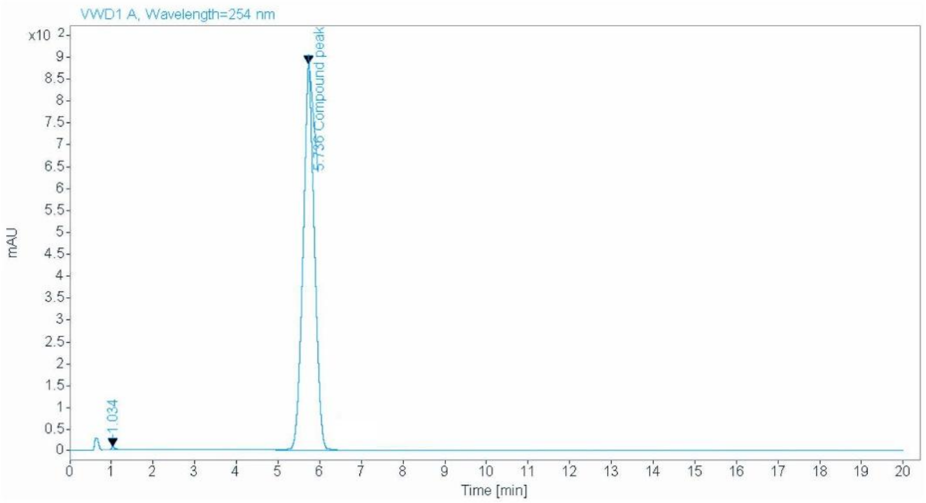

Signal: VWD1 A, Wavelength=254 nm

RT [min]	Type	Width [min]	Area	Height	Area%	Name
1.034	BV	0.0868	44.6291	8.2231	0.2045	
5.736	VB	0.3404	21780.9163	882.1411	99.7955	Compound peak
		Sum	21825.5454			

Figure S60. HPLC spectrum of compound **10b**

Area Percent Report

Agilent Technologies

**Data file:**	C:\CHEM32\1\DATA\HAYTHAM\10C-003.D		
**Sample name:**	10c		
**Description:**	Solvent: DMSO + ACN, Flow rate: 1.5 mL/min<br>Mobile phase: 35% ACN + 65% Phosphate buffer		
**Sample amount:**	2.500	**Sample type:**	Sample
**Instrument:**	HPLC	**Location:**	Vial 10
**Injection date:**	4/29/2025 2:03:08 AM	**Injection:**	1 of 1
**Acq. method:**	HAYTHAM.M	**Injection volume:**	1.000
**Analysis method:**	HAYTHAM.M	**Acq. operator:**	SYSTEM
**Last changed:**	4/29/2025 4:45:01 AM<br>(modified after loading)		

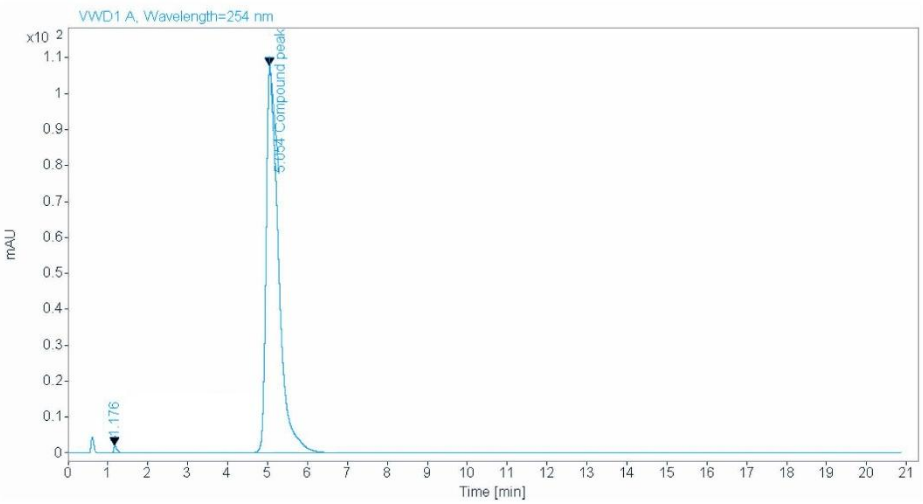

Signal: VWD1 A, Wavelength=254 nm

RT [min]	Type	Width [min]	Area	Height	Area%	Name
1.176	BV	0.1016	15.0112	2.4991	0.6729	
5.054	VV	0.2989	2215.8843	106.4907	99.3271	Compound peak
		Sum	2230.8955			

Figure S61. HPLC spectrum of compound **10c**

Area Percent Report

Agilent Technologies

Data file:	C:\CHEM32\1\DATA\HAYTHAM\10D-001.D		
Sample name:	10d		
Description:	Solvent: DMSO + ACN, Flow rate: 1.5 mL/min<br>Mobile phase: 35% ACN + 65% Phosphate buffer		
Sample amount:	2.500	Sample type:	Sample
Instrument:	HPLC	Location:	Vial 11
Injection date:	4/29/2025 1:21:40 AM	Injection:	1 of 1
Acq. method:	HAYTHAM.M	Injection volume:	5.000
Analysis method:	HAYTHAM.M	Acq. operator:	SYSTEM
Last changed:	4/29/2025 4:30:42 AM<br>(modified after loading)		

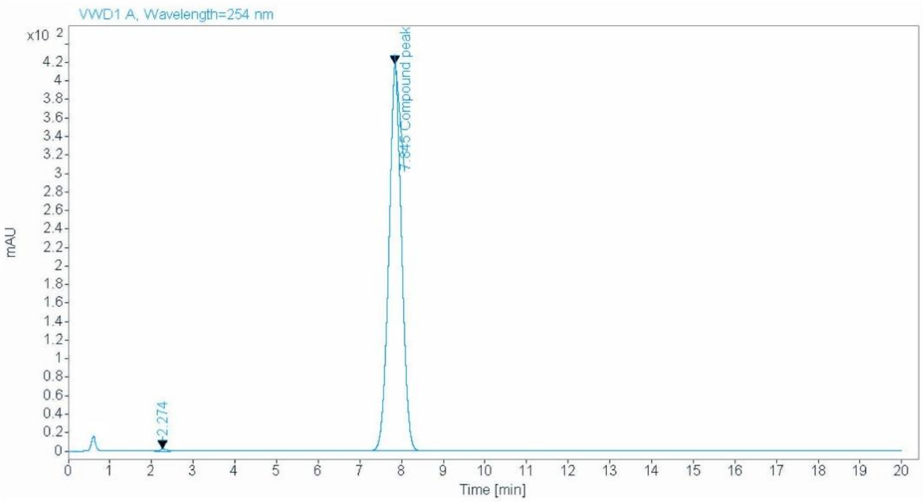

Signal: VWD1 A, Wavelength=254 nm

RT [min]	Type	Width [min]	Area	Height	Area%	Name
2.274	VV	0.1837	23.1214	2.0103	0.1845	
7.845	VV	0.3667	12511.5370	417.8322	99.8155	Compound peak
		Sum	12534.6584			

Figure S62. HPLC spectrum of compound **10d**

Area Percent Report

Agilent Technologies

Data file:	C:\CHEM32\1\DATA\HAYTHAM\10E-001.D		
Sample name:	10e		
Description:	Solvent: DMSO + ACN, Flow rate: 1.5 mL/min<br>Mobile phase: 35% ACN + 65% Phosphate buffer		
Sample amount:	2.500	Sample type:	Sample
Instrument:	HPLC	Location:	Vial 12
Injection date:	4/29/2025 11:40:14 AM	Injection:	1 of 1
Acq. method:	HAYTHAM.M	Injection volume:	5.000
Analysis method:	HAYTHAM.M	Acq. operator:	SYSTEM
Last changed:	4/29/2025 12:01:31 AM<br>(modified after loading)		

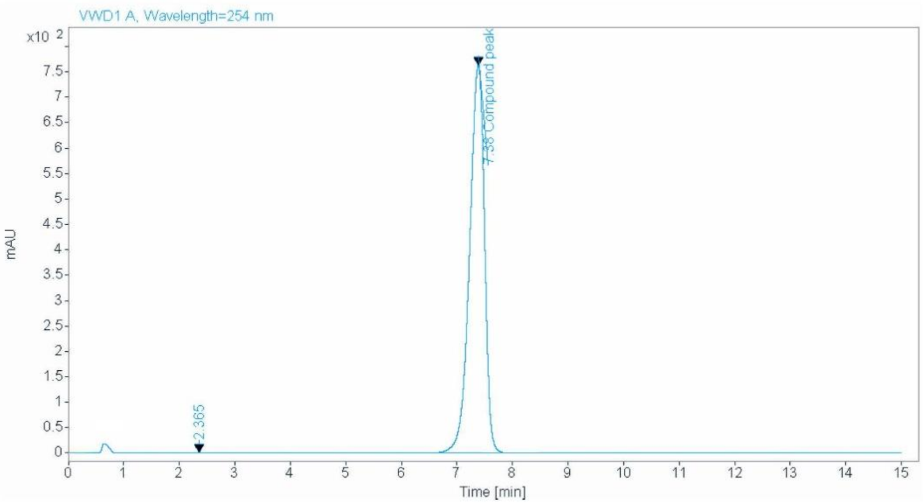

Signal: VWD1 A, Wavelength=254 nm

RT [min]	Type	Width [min]	Area	Height	Area%	Name
2.365	VB	0.0798	6.9937	1.4561	0.0489	
7.380	VB	0.2973	14314.5010	762.0634	99.9511	Compound peak
		Sum	14321.4947			

Figure S63. HPLC spectrum of compound **10e**

Area Percent Report

Agilent Technologies

Data file:	C:\CHEM32\1\DATA\HAYTHAM\10F-003.D		
Sample name:	10f		
Description:	Solvent: DMSO + ACN, Flow rate: 1.5 mL/min<br>Mobile phase: 35% ACN + 65% Phosphate buffer		
Sample amount:	2.500	Sample type:	Sample
Instrument:	HPLC	Location:	Vial 13
Injection date:	4/29/2025 3:32:50 AM	Injection:	1 of 1
Acq. method:	HAYTHAM.M	Injection volume:	5.000
Analysis method:	HAYTHAM.M	Acq. operator:	SYSTEM
Last changed:	4/29/2025 4:12:34 AM<br>(modified after loading)		

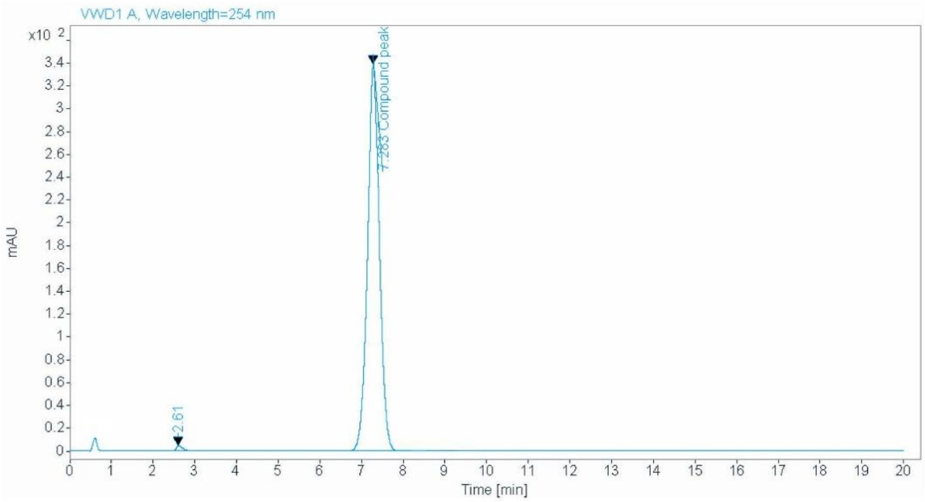

Signal: VWD1 A, Wavelength=254 nm

RT [min]	Type	Width [min]	Area	Height	Area%	Name
2.610	VV	0.1804	51.9178	4.5290	0.5728	
7.283	BV	0.3404	9012.5012	339.0104	99.4272	Compound peak
		Sum	9064.4190			

Figure S64. HPLC spectrum of compound **10f**

Area Percent Report

Agilent Technologies

**Data file:**	C:\CHEM32\1\DATA\HAYTHAM\10G-002.D		
**Sample name:**	10g		
**Description:**	Solvent: DMSO + ACN, Flow rate: 1.5 mL/min<br>Mobile phase: 35% ACN + 65% Phosphate buffer		
**Sample amount:**	2.500	**Sample type:**	Sample
**Instrument:**	HPLC	**Location:**	Vial 14
**Injection date:**	4/29/2025 4:26:32 AM	**Injection:**	1 of 1
**Acq. method:**	HAYTHAM.M	**Injection volume:**	5.000
**Analysis method:**	HAYTHAM.M	**Acq. operator:**	SYSTEM
**Last changed:**	4/29/2025 4:33:57 AM<br>(modified after loading)		

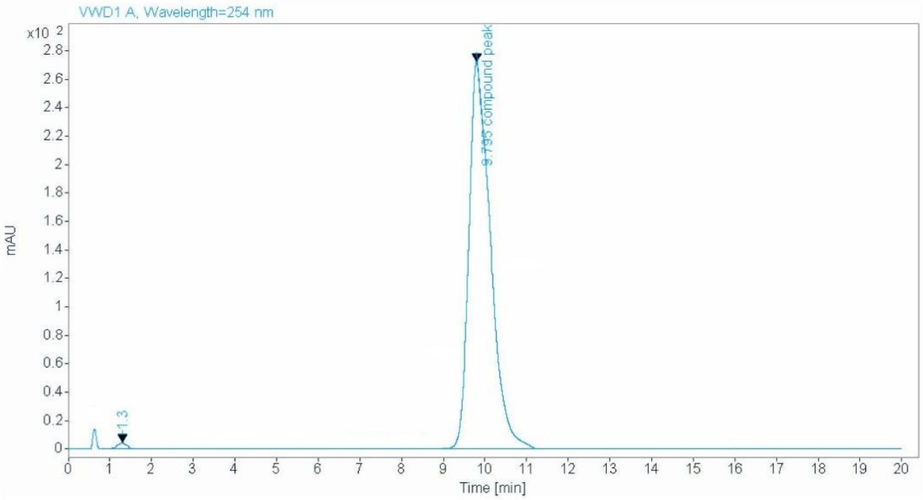

Signal: VWD1 A, Wavelength=254 nm

RT [min]	Type	Width [min]	Area	Height	Area%	Name
1.300	BV	0.2665	72.3911	3.5147	0.6770	
9.795	VV	0.5309	10622.0169	268.8050	99.3230	compound peak
		Sum	10694.4080			

Figure S65. HPLC spectrum of compound **10g**

Area Percent Report

Agilent Technologies

Data file:	C:\CHEM32\1\DATA\HAYTHAM\12-002.D		
Sample name:	12		
Description:	Solvent: DMSO + ACN, Flow rate: 1.5 mL/min<br>Mobile phase: 35% ACN + 65% Phosphate buffer		
Sample amount:	2.500	Sample type:	Sample
Instrument:	HPLC	Location:	Vial 15
Injection date:	4/29/2025 2:20:14 AM	Injection:	1 of 1
Acq. method:	HAYTHAM.M	Injection volume:	5.000
Analysis method:	HAYTHAM.M	Acq. operator:	SYSTEM
Last changed:	4/29/2025 2:40:50 AM		

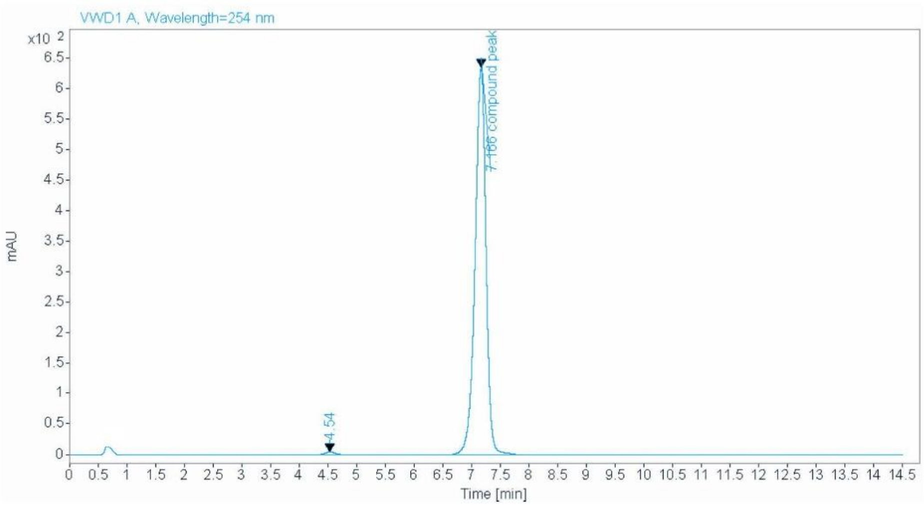

Signal: VWD1 A, Wavelength=254 nm

RT [min]	Type	Width [min]	Area	Height	Area%	Name
4.540	BV	0.1970	61.2690	4.6703	0.7405	
7.166	VB	0.2025	8213.7191	634.6093	99.2595	compound peak
		Sum	8274.9881			

Figure S66. HPLC spectrum of compound **12**

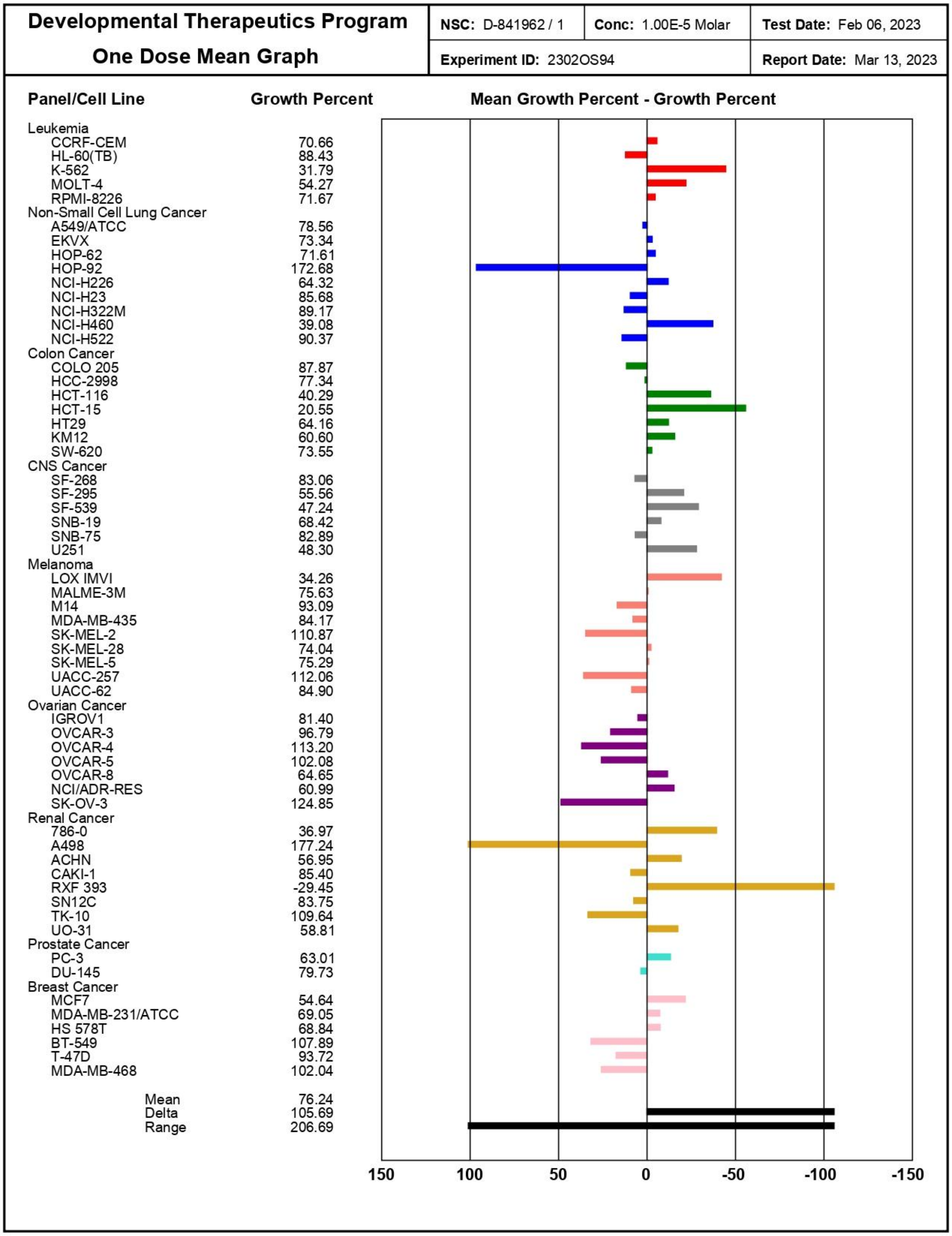

Figure S67. One dose mean graph for compound **8a** at 10 µM

Developmental Therapeutics Program One Dose Mean Graph	NSC: D-841963 / 1	Conc: 1.00E-5 Molar	Test Date: Feb 06, 2023
	Experiment ID: 2302OS94		Report Date: Mar 13, 2023

Panel/Cell Line	Growth Percent
**Leukemia**	
CCRF-CEM	20.90
HL-60(TB)	0.13
K-562	5.54
MOLT-4	18.00
RPMI-8226	-8.98
**Non-Small Cell Lung Cancer**	
A549/ATCC	4.07
EKVX	-17.28
HOP-62	-11.65
HOP-92	-4.91
NCI-H226	-42.68
NCI-H23	-60.83
NCI-H322M	-18.19
NCI-H460	7.56
NCI-H522	10.62
**Colon Cancer**	
COLO 205	-66.33
HCC-2998	-74.66
HCT-116	-11.32
HCT-15	-32.08
HT29	-40.55
KM12	-74.56
SW-620	-62.35
**CNS Cancer**	
SF-268	35.64
SF-295	-36.35
SF-539	-48.17
SNB-19	-3.80
SNB-75	35.25
U251	-56.91
**Melanoma**	
LOX IMVI	-93.11
MALME-3M	-64.50
M14	-22.61
MDA-MB-435	-23.95
SK-MEL-2	33.94
SK-MEL-28	-54.92
SK-MEL-5	-78.72
UACC-257	41.28
UACC-62	-79.30
**Ovarian Cancer**	
IGROV1	-23.31
OVCAR-3	-0.73
OVCAR-4	5.66
OVCAR-5	-29.67
OVCAR-8	-42.01
NCI/ADR-RES	-7.41
SK-OV-3	43.93
**Renal Cancer**	
786-0	7.98
A498	-3.37
ACHN	-23.39
CAKI-1	1.46
RXF 393	-42.03
SN12C	-80.96
TK-10	32.91
UO-31	-15.44
**Prostate Cancer**	
PC-3	-0.99
DU-145	-66.35
**Breast Cancer**	
MCF7	-17.95
MDA-MB-231/ATCC	-71.91
HS 578T	-12.35
BT-549	-2.31
T-47D	17.55
MDA-MB-468	-15.45
Mean	-20.71
Delta	72.40
Range	137.04

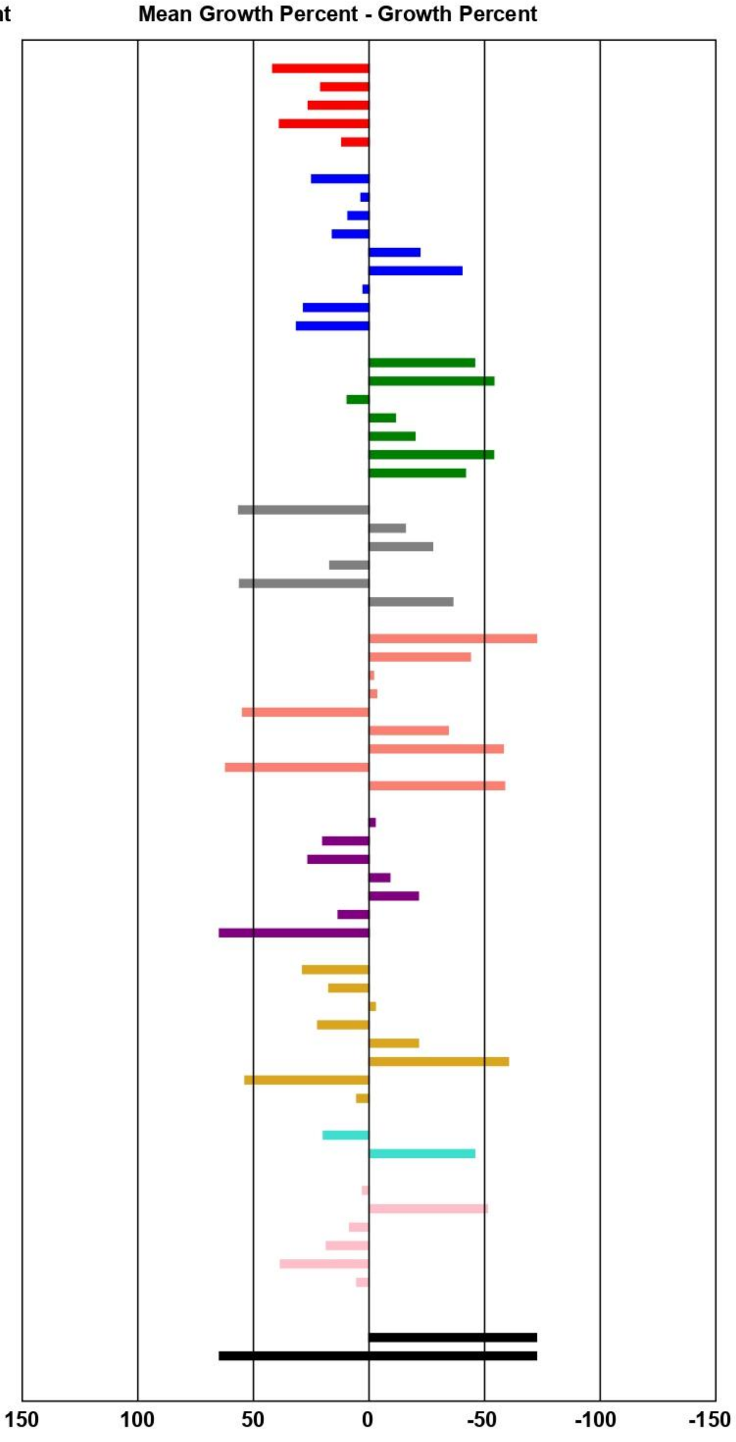

Figure S68. One dose mean graph for compound **8b** at 10 μM

National Cancer Institute Developmental Therapeutics Program In-Vitro Testing Results			
NSC : D - 841963 / 1	Experiment ID : 2307NS43	Test Type : 08	Units : Molar
Report Date : March 16, 2024	Test Date : July 10, 2023	QNS :	MC :
COMI : H1-8b	Stain Reagent : SRB Dual-Pass Related	SSPL : 1AJN	

			Log10 Concentration												
	Time		Mean Optical Densities					Percent Growth							
Panel/Cell Line	Zero	Ctrl	-8.0	-7.0	-6.0	-5.0	-4.0	-8.0	-7.0	-6.0	-5.0	-4.0	GI50	TGI	LC50
**Leukemia**															
CCRF-CEM	0.418	1.849	1.883	1.924	1.632	0.463	0.555	102	105	85	3	10	2.67E-6	> 1.00E-4	> 1.00E-4
HL-60(TB)	0.393	1.465	1.448	1.431	1.365	0.558	0.389	98	97	91	15	-1	3.47E-6	8.67E-5	> 1.00E-4
K-562	0.218	1.895	1.811	1.889	1.698	0.215	0.339	95	100	88	-1	7	2.67E-6	.	> 1.00E-4
MOLT-4	0.497	1.902	1.961	1.782	1.628	0.676	0.572	104	91	81	13	5	2.82E-6	> 1.00E-4	> 1.00E-4
RPMI-8226	0.736	2.670	2.706	2.759	2.583	0.715	0.792	102	105	96	-3	3	2.90E-6	.	> 1.00E-4
SR	0.438	2.063	2.071	1.989	1.902	0.481	0.412	100	95	90	3	-6	2.87E-6	2.02E-5	> 1.00E-4
**Non-Small Cell Lung Cancer**															
A549/ATCC	0.509	2.478	2.340	2.436	2.269	0.557	0.256	93	98	89	2	-50	2.84E-6	1.11E-5	> 1.00E-4
EKVX	0.807	1.868	1.774	1.804	1.754	0.681	0.237	91	94	89	-16	-71	2.37E-6	7.10E-6	4.22E-5
HOP-62	0.734	2.541	2.623	2.616	2.349	0.600	0.268	105	104	89	-18	-64	2.32E-6	6.76E-6	5.02E-5
HOP-92	1.384	1.761	1.706	1.713	1.626	0.900	0.337	85	87	64	-35	-76	1.39E-6	4.43E-6	2.34E-5
NCI-H226	0.974	1.490	1.370	1.501	1.412	0.802	0.380	77	102	85	-18	-61	2.19E-6	6.72E-6	5.57E-5
NCI-H23	0.837	1.931	1.862	1.898	1.820	0.645	0.146	94	97	90	-23	-83	2.25E-6	6.26E-6	2.84E-5
NCI-H322M	0.529	1.859	1.707	1.781	1.606	0.456	0.010	89	94	81	-14	-98	2.12E-6	7.14E-6	2.68E-5
NCI-H460	0.284	2.720	2.795	2.704	2.580	0.215	0.167	103	99	94	-24	-41	2.36E-6	6.24E-6	> 1.00E-4
NCI-H522	1.097	2.826	2.613	2.725	2.575	1.014	0.222	88	94	85	-8	-80	2.40E-6	8.28E-6	3.87E-5
**Colon Cancer**															
COLO 205	0.517	2.472	2.427	2.347	2.095	0.188	0.152	98	94	81	-64	-71	1.63E-6	3.62E-6	8.03E-6
HCC-2998	0.628	1.649	1.610	1.551	1.727	0.262	0.004	96	90	108	-58	-99	2.22E-6	4.45E-6	8.90E-6
HCT-116	0.186	2.100	1.642	2.549	1.855	0.159	0.153	76	123	87	-15	-18	2.32E-6	7.20E-6	> 1.00E-4
HCT-15	0.301	1.782	1.766	1.789	1.627	0.165	0.013	99	101	90	-45	-96	1.97E-6	4.62E-6	1.24E-5
HT29	0.324	2.154	2.098	2.189	2.194	0.238	0.099	97	102	102	-27	-69	2.54E-6	6.21E-6	3.51E-5
KM12	0.481	2.402	2.310	2.305	2.097	0.065	0.019	95	95	84	-86	-96	1.59E-6	3.11E-6	6.11E-6
SW-620	0.294	2.269	2.221	2.204	2.093	1.209	1.071	98	97	91	46	39	8.28E-6	> 1.00E-4	> 1.00E-4
**CNS Cancer**															
SF-268	1.122	2.845	2.710	2.755	2.678	1.119	0.059	92	95	90	0	-95	2.79E-6	9.93E-6	3.36E-5
SF-295	0.657	2.157	1.957	2.089	2.070	0.365	0.090	87	95	94	-45	-86	2.08E-6	4.78E-6	1.35E-5
SF-539	0.796	2.454	2.454	2.429	2.269	0.142	0.025	100	98	89	-82	-97	1.69E-6	3.31E-6	6.48E-6
SNB-75	1.106	1.966	1.802	1.847	1.691	1.104	0.101	81	86	68	0	-91	1.84E-6	9.94E-6	3.54E-5
U251	0.366	1.954	1.893	1.945	1.820	0.098	0.102	96	99	92	-73	-72	1.79E-6	3.59E-6	7.23E-6
**Melanoma**															
LOX IMVI	0.434	2.234	2.067	2.136	1.922	0.024	0.017	91	95	83	-95	-96	1.53E-6	2.93E-6	5.60E-6
MALME-3M	0.419	1.009	0.923	0.978	0.950	0.283	0.038	85	95	90	-32	-91	2.12E-6	5.43E-6	1.99E-5
M14	0.546	2.315	2.280	2.426	2.346	0.348	0.204	98	106	102	-36	-63	2.37E-6	5.46E-6	3.30E-5
MDA-MB-435	0.524	2.290	2.257	2.283	2.164	0.393	0.026	98	100	93	-25	-95	2.31E-6	6.14E-6	2.27E-5
SK-MEL-2	1.307	3.102	3.097	3.090	3.037	0.897	0.038	100	99	96	-31	-97	2.31E-6	5.68E-6	1.92E-5
SK-MEL-28	0.650	1.946	1.948	2.053	1.905	0.706	0.011	100	108	97	4	-98	3.21E-6	1.10E-5	3.38E-5
SK-MEL-5	0.961	2.839	2.725	2.852	2.789	0.511	.	94	101	97	-47	-100	2.13E-6	4.73E-6	1.14E-5
UACC-257	1.082	2.650	2.534	2.629	2.489	1.476	0.036	93	99	90	25	-97	4.12E-6	1.61E-5	4.14E-5
UACC-62	0.762	2.283	2.241	2.264	2.043	0.295	0.027	97	99	84	-61	-96	1.72E-6	3.79E-6	8.36E-6
**Ovarian Cancer**															
IGROV1	0.228	1.413	1.302	1.258	1.143	0.165	0.066	91	87	77	-28	-71	1.82E-6	5.43E-6	3.24E-5
OVCAR-3	0.589	1.965	1.889	1.953	1.873	0.344	0.009	95	99	93	-42	-98	2.09E-6	4.92E-6	1.41E-5
OVCAR-4	0.909	2.118	2.030	2.103	2.022	0.983	0.095	93	99	92	6	-90	3.08E-6	1.16E-5	3.86E-5
OVCAR-5	0.511	1.538	1.559	1.557	1.483	0.415	0.030	102	102	95	-19	-94	2.47E-6	6.82E-6	2.59E-5
OVCAR-8	0.699	2.815	2.763	2.813	2.677	0.615	0.017	98	100	93	-12	-98	2.58E-6	7.69E-6	2.78E-5
NCI/ADR-RES	0.445	1.316	1.285	1.315	1.241	0.294	0.122	96	100	91	-34	-73	2.14E-6	5.35E-6	2.59E-5
SK-OV-3	1.032	2.620	2.532	2.483	2.357	1.416	0.634	94	91	83	24	-39	3.67E-6	2.43E-5	> 1.00E-4
**Renal Cancer**															
786-0	0.909	3.063	3.055	3.149	3.123	0.898	0.242	100	104	103	-1	-73	3.22E-6	9.72E-6	4.74E-5
A498	1.400	2.555	2.540	2.425	2.515	1.736	0.288	99	89	97	29	-79	4.90E-6	1.85E-5	5.35E-5
ACHN	0.301	1.472	1.508	1.525	1.325	0.267	0.012	103	104	87	-11	-96	2.39E-6	7.68E-6	2.86E-5
CAKI-1	0.799	2.638	2.492	2.560	2.249	0.903	0.146	92	96	79	6	-82	2.48E-6	1.16E-5	4.33E-5
RXF 393	0.921	1.440	1.348	1.412	1.397	0.622	0.270	82	95	92	-33	-71	2.17E-6	5.47E-6	2.87E-5
SN12C	0.640	2.215	2.190	2.158	1.961	0.118	0.049	98	96	84	-82	-92	1.60E-6	3.21E-6	6.44E-6
UO-31	0.571	1.918	1.698	1.689	1.541	0.505	0.032	84	83	72	-12	-94	1.83E-6	7.26E-6	2.91E-5
**Prostate Cancer**															
PC-3	0.621	2.022	1.865	1.942	1.684	0.413	0.256	89	94	76	-34	-59	1.72E-6	4.93E-6	4.46E-5
DU-145	0.467	1.923	1.893	1.984	1.848	0.299	0.037	98	104	95	-36	-92	2.20E-6	5.30E-6	1.77E-5
**Breast Cancer**															
MCF7	0.336	1.693	1.607	1.623	1.409	0.257	0.105	94	95	79	-24	-69	1.92E-6	5.88E-6	3.82E-5
MDA-MB-231/ATCC	0.524	1.176	1.189	1.176	1.108	0.282	0.076	102	100	90	-46	-86	1.96E-6	4.57E-6	1.25E-5
HS 578T	1.160	2.278	2.279	2.247	2.176	0.256	0.064	100	97	91	-78	-94	1.75E-6	3.45E-6	6.83E-6
BT-549	1.006	1.992	1.953	2.019	2.002	0.932	0.476	96	103	101	-7	-53	2.96E-6	8.55E-6	8.70E-5
T-47D	0.718	1.977	1.901	1.947	1.837	0.686	0.368	94	98	89	-4	-49	2.61E-6	8.96E-6	> 1.00E-4
MDA-MB-468	0.720	1.061	1.024	1.047	1.037	0.584	0.058	89	96	93	-19	-92	2.43E-6	6.78E-6	2.67E-5

Figure S69. Values of log molar concentration of response parameters (log_{10} GI_{50}, log_{10} TGI & log_{10} LC_{50}) for compound **8b**

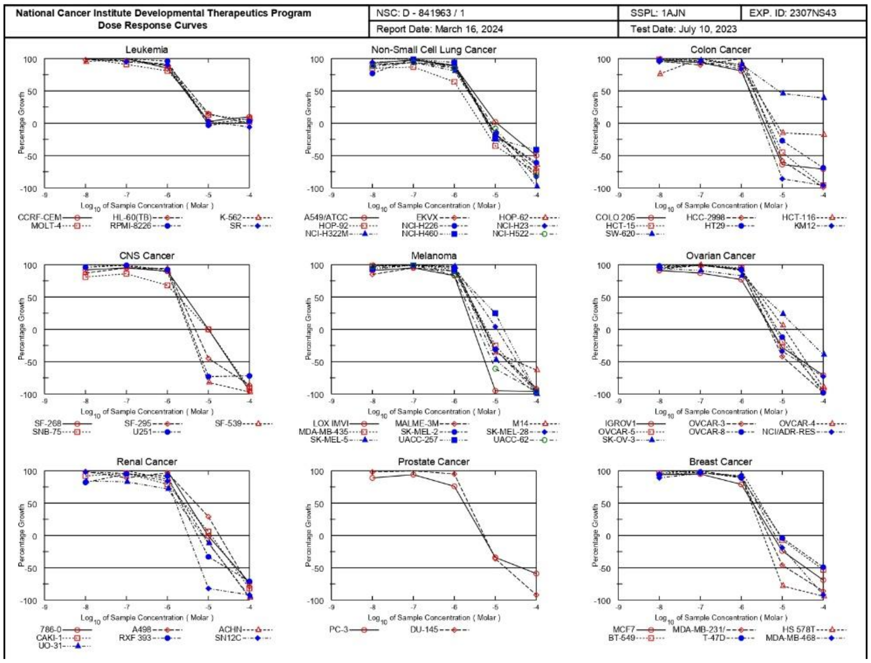

Figure S70. Dose-response curves (% growth versus sample concentration) for all cell lines with different subpanel obtained from the NCI's in vitro disease-oriented human cancer cells line for compound **8b** on nine types of cancer

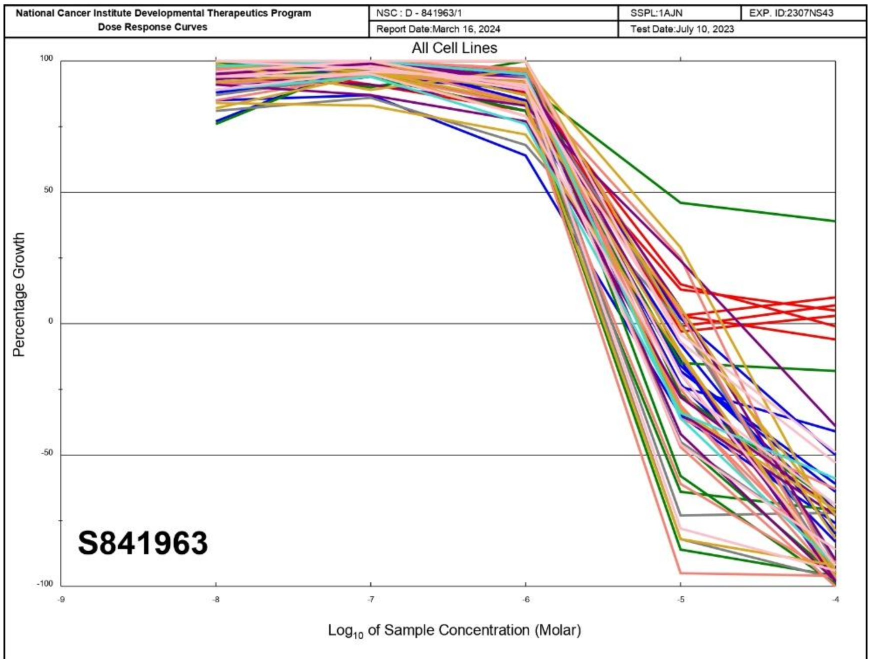

Figure S71. Dose-response curves for all cell lines in the NCI60 panel exposed compound **8b** with tissue originated colors and shapes.

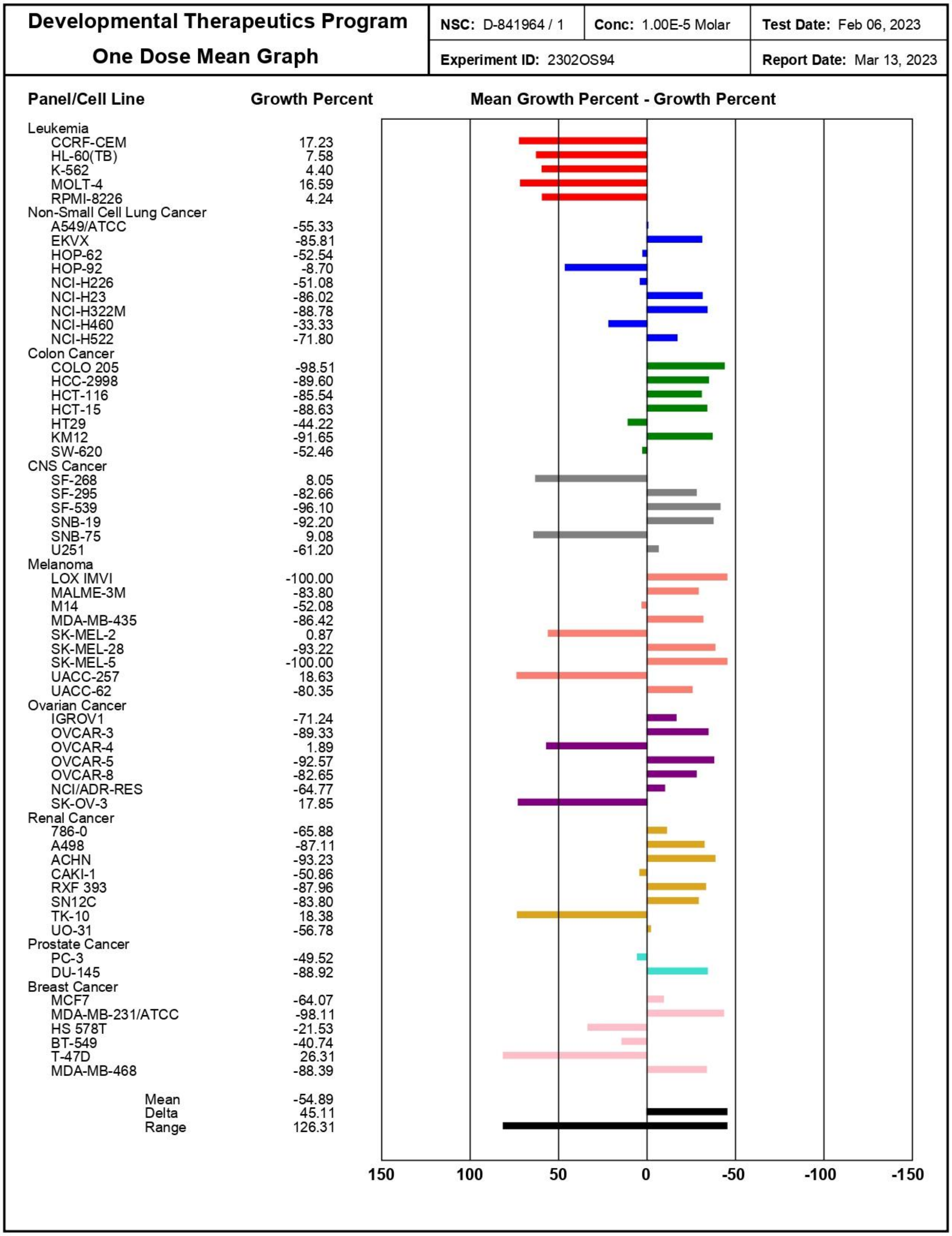

Figure S72. One dose mean graph for compound **8c** at 10 μM

National Cancer Institute Developmental Therapeutics Program
In-Vitro Testing Results

NSC : D - 841964 / 1	Experiment ID : 2307NS43	Test Type : 08	Units : Molar
Report Date : March 16, 2024	Test Date : July 10, 2023	QNS :	MC :
COMI : H1-8c	Stain Reagent : SRB Dual-Pass Related	SSPL : 1AJN	

			Log10 Concentration													
	Time		Mean Optical Densities					Percent Growth								
Panel/Cell Line	Zero	Ctrl	-8.0	-7.0	-6.0	-5.0	-4.0	-8.0	-7.0	-6.0	-5.0	-4.0		GI50	TGI	LC50
**Leukemia**																
CCRF-CEM	0.418	2.115	2.112	1.633	0.911	0.491	0.633	100	72	29	4	13		3.21E-7	> 1.00E-4	> 1.00E-4
HL-60(TB)	0.393	1.484	1.439	1.472	0.817	0.527	0.371	96	99	39	12	-6		6.52E-7	4.81E-5	> 1.00E-4
K-562	0.218	1.869	1.916	1.891	0.602	0.218	0.285	103	101	23	0	4		4.54E-7	.	> 1.00E-4
MOLT-4	0.497	1.991	1.928	1.780	1.003	0.808	0.547	96	86	34	21	3		4.89E-7	> 1.00E-4	> 1.00E-4
RPMI-8226	0.736	2.729	2.823	2.997	1.662	1.289	1.006	105	113	46	28	14		8.86E-7	> 1.00E-4	> 1.00E-4
SR	0.438	2.247	2.184	2.139	0.972	0.460	0.582	97	94	29	1	8		4.81E-7	> 1.00E-4	> 1.00E-4
**Non-Small Cell Lung Cancer**																
A549/ATCC	0.509	2.482	2.470	2.452	2.063	0.168	0.148	99	99	79	-67	-71		1.57E-6	3.47E-6	7.63E-6
EKVX	0.807	1.880	1.815	1.847	1.452	0.514	0.272	94	97	60	-36	-66		1.27E-6	4.20E-6	2.86E-5
HOP-62	0.734	2.598	2.372	2.535	1.810	0.275	0.405	88	97	58	-63	-45		1.16E-6	3.02E-6	.
HOP-92	1.384	1.790	1.730	1.713	1.762	1.029	0.201	85	81	93	-26	-85		2.31E-6	6.08E-6	2.55E-5
NCI-H226	0.974	1.598	1.489	1.517	1.554	0.341	0.340	82	87	93	-65	-65		1.87E-6	3.88E-6	8.04E-6
NCI-H23	0.837	1.956	1.854	1.865	1.639	0.297	0.122	91	92	72	-65	-85		1.44E-6	3.36E-6	7.82E-6
NCI-H322M	0.529	1.843	1.917	1.858	1.833	0.111	0.043	106	101	99	-79	-92		1.89E-6	3.60E-6	6.87E-6
NCI-H460	0.284	2.731	2.836	2.793	1.816	0.114	0.146	104	103	63	-60	-49		1.27E-6	3.25E-6	.
NCI-H522	1.097	2.715	2.580	2.524	2.496	0.272	0.318	92	88	86	-75	-71		1.68E-6	3.43E-6	6.98E-6
**Colon Cancer**																
COLO 205	0.517	2.441	2.506	2.387	2.390	0.233	0.281	103	97	97	-55	-46		2.05E-6	4.36E-6	.
HCC-2998	0.628	1.699	1.680	1.676	1.560	0.021	0.033	98	98	87	-97	-95		1.59E-6	2.98E-6	5.57E-6
HCT-116	0.186	1.731	1.800	1.703	1.153	0.130	0.218	104	98	63	-30	2		1.37E-6	.	> 1.00E-4
HCT-15	0.301	1.799	1.766	1.639	1.199	0.028	0.019	98	89	60	-91	-94		1.16E-6	2.50E-6	5.36E-6
HT29	0.324	2.106	2.001	2.044	1.453	0.059	0.114	94	97	63	-82	-65		1.24E-6	2.73E-6	6.04E-6
KM12	0.481	2.487	2.581	2.512	1.868	0.088	0.190	105	101	69	-82	-60		1.34E-6	2.87E-6	6.16E-6
SW-620	0.294	2.259	2.249	2.151	1.429	0.997	0.822	100	94	58	36	27		2.26E-6	> 1.00E-4	> 1.00E-4
**CNS Cancer**																
SF-268	1.122	2.923	2.880	2.893	2.674	1.512	0.521	98	98	86	22	-54		3.64E-6	1.94E-5	8.95E-5
SF-295	0.657	2.153	2.020	1.894	0.617	0.134	0.091	91	83	-6	-80	-86		2.33E-7	8.52E-7	3.95E-6
SF-539	0.796	2.461	2.335	1.984	1.268	0.116	0.043	92	71	28	-85	-95		3.14E-7	1.77E-6	4.88E-6
SNB-75	1.106	2.007	1.740	1.656	1.392	1.078	0.143	70	61	32	-3	-87		2.38E-7	8.44E-6	3.64E-5
U251	0.366	1.967	1.981	1.697	1.028	0.045	0.078	101	83	41	-88	-79		6.20E-7	2.09E-6	5.10E-6
**Melanoma**																
LOX IMVI	0.434	2.259	2.096	2.094	1.612	-0.001	0.028	91	91	65	-100	-94		1.23E-6	2.47E-6	4.97E-6
MALME-3M	0.419	1.053	1.141	1.283	1.209	0.171	0.154	114	136	125	-59	-63		2.54E-6	4.76E-6	8.90E-6
M14	0.546	2.300	2.286	2.258	1.846	0.278	0.297	99	98	74	-49	-46		1.57E-6	3.99E-6	> 1.00E-4
MDA-MB-435	0.524	2.366	2.233	2.166	1.760	0.089	0.083	93	89	67	-83	-84		1.30E-6	2.80E-6	6.03E-6
SK-MEL-2	1.307	3.085	3.050	3.032	2.603	0.521	0.150	98	97	73	-60	-89		1.49E-6	3.53E-6	8.39E-6
SK-MEL-28	0.650	1.956	1.921	1.860	1.575	0.106	0.007	97	93	71	-84	-99		1.36E-6	2.87E-6	6.05E-6
SK-MEL-5	0.961	2.899	2.810	2.832	2.740	0.014	0.007	95	97	92	-99	-99		1.66E-6	3.03E-6	5.56E-6
UACC-257	1.082	2.663	2.604	2.574	2.558	1.497	0.151	96	94	93	26	-86		4.43E-6	1.71E-5	4.77E-5
UACC-62	0.762	2.302	2.232	2.179	1.842	0.181	0.033	95	92	70	-76	-96		1.37E-6	3.01E-6	6.61E-6
**Ovarian Cancer**																
IGROV1	0.228	1.376	1.451	1.670	1.200	0.193	0.307	107	126	85	-16	7		2.22E-6	.	> 1.00E-4
OVCAR-3	0.589	2.046	2.120	2.011	1.804	0.126	0.095	105	98	83	-79	-84		1.61E-6	3.27E-6	6.66E-6
OVCAR-4	0.909	2.128	2.062	1.932	1.858	1.110	0.159	95	84	78	16	-83		2.84E-6	1.47E-5	4.69E-5
OVCAR-5	0.511	1.441	1.414	1.359	1.270	0.072	0.003	97	91	82	-86	-99		1.54E-6	3.07E-6	6.10E-6
OVCAR-8	0.699	2.802	2.837	2.812	2.482	0.551	0.144	102	100	85	-21	-79		2.13E-6	6.30E-6	3.12E-5
NCI/ADR-RES	0.445	1.339	1.348	1.275	1.268	0.164	0.081	101	93	92	-63	-82		1.87E-6	3.92E-6	8.23E-6
SK-OV-3	1.032	2.524	2.515	2.371	2.150	1.175	0.304	99	90	75	10	-71		2.41E-6	1.32E-5	5.53E-5
**Renal Cancer**																
786-0	0.909	3.028	3.031	2.598	1.487	0.271	0.307	100	80	27	-70	-66		3.68E-7	1.90E-6	6.20E-6
A498	1.400	2.517	2.391	2.441	2.085	0.300	0.267	89	93	61	-79	-81		1.20E-6	2.74E-6	6.25E-6
ACHN	0.301	1.478	1.503	1.465	0.925	0.213	0.002	102	99	53	-29	-100		1.09E-6	4.40E-6	1.97E-5
CAKI-1	0.799	2.704	2.610	2.518	1.907	0.369	0.081	95	90	58	-54	-90		1.18E-6	3.31E-6	9.24E-6
RXF 393	0.921	1.484	1.470	1.253	0.991	0.197	0.234	97	59	12	-79	-75		1.56E-7	1.37E-6	4.85E-6
SN12C	0.640	2.208	2.062	2.094	2.042	0.077	0.058	91	93	89	-88	-91		1.67E-6	3.19E-6	6.10E-6
UO-31	0.571	1.935	1.740	1.887	1.235	0.225	0.056	86	97	49	-61	-90		9.38E-7	2.79E-6	8.00E-6
**Prostate Cancer**																
PC-3	0.621	2.033	2.013	1.938	1.404	0.120	0.149	99	93	55	-81	-76		1.10E-6	2.55E-6	5.94E-6
DU-145	0.467	2.022	2.094	2.019	1.575	0.127	0.081	105	100	71	-73	-83		1.40E-6	3.12E-6	6.95E-6
**Breast Cancer**																
MCF7	0.336	1.759	1.597	1.575	0.895	0.067	0.112	89	87	39	-80	-67		5.97E-7	2.13E-6	5.59E-6
MDA-MB-231/ATCC	0.524	1.168	1.168	1.164	1.015	0.084	0.104	100	99	76	-84	-80		1.46E-6	2.99E-6	6.13E-6
HS 578T	1.160	2.309	2.309	2.330	1.692	0.112	0.175	100	102	46	-90	-85		8.57E-7	2.18E-6	5.07E-6
BT-549	1.006	1.957	1.955	1.879	1.587	0.544	0.374	100	92	61	-46	-63		1.27E-6	3.72E-6	1.74E-5
T-47D	0.718	2.108	2.070	1.922	1.876	0.840	0.667	97	87	83	9	-7		2.80E-6	3.56E-5	> 1.00E-4
MDA-MB-468	0.720	1.101	1.092	1.076	1.093	0.125	0.124	98	93	98	-83	-83		1.84E-6	3.48E-6	6.59E-6

Figure S73. Values of log molar concentration of response parameters ($\log_{10} GI_{50}$, $\log_{10}$ TGI & $\log_{10} LC_{50}$) for compound **8c**

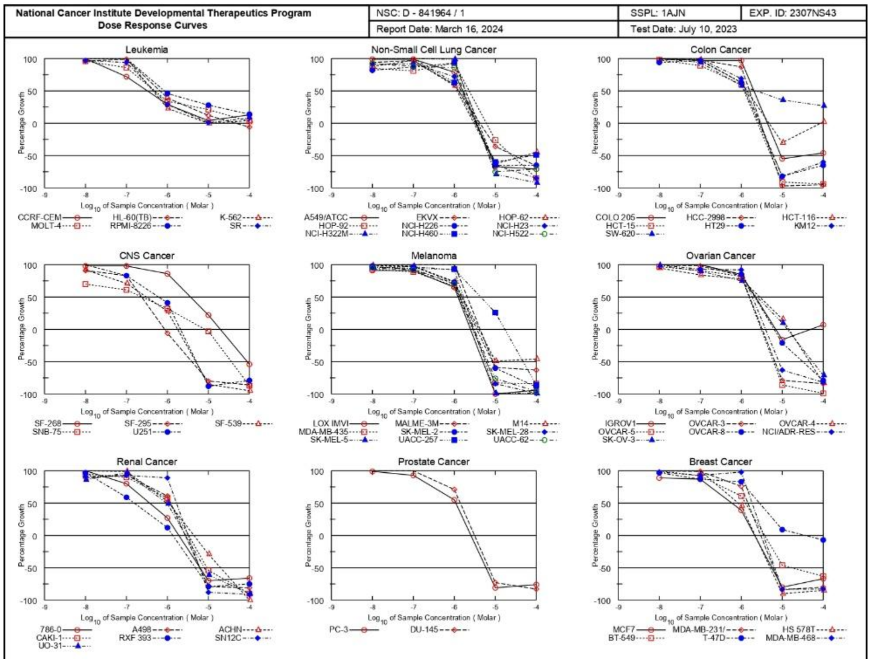

Figure S74. Dose-response curves (% growth versus sample concentration) for all cell lines with different subpanel obtained from the NCI's in vitro disease-oriented human cancer cells line for compound **8c** on nine types of cancer

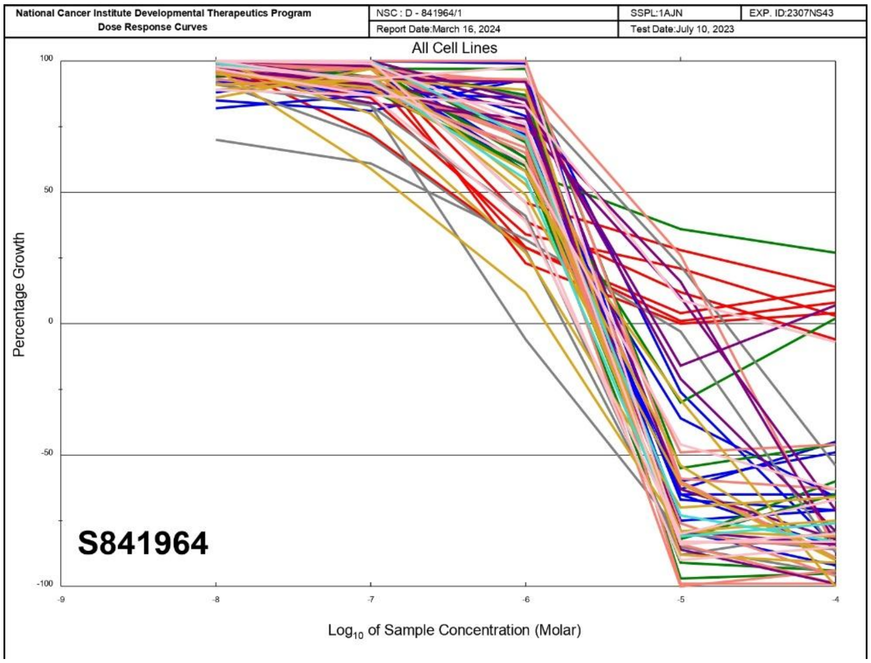

Figure S75. Dose-response curves for all cell lines in the NCI60 panel exposed compound **8c** with tissue originated colors and shapes.

Developmental Therapeutics Program One Dose Mean Graph	NSC: D-841966 / 1	Conc: 1.00E-5 Molar	Test Date: Feb 06, 2023
	Experiment ID: 2302OS94		Report Date: Mar 13, 2023

Panel/Cell Line	Growth Percent
**Leukemia**	
CCRF-CEM	104.38
HL-60(TB)	115.62
K-562	94.19
MOLT-4	109.20
RPMI-8226	85.08
**Non-Small Cell Lung Cancer**	
A549/ATCC	96.33
EKVX	85.04
HOP-62	78.97
HOP-92	98.97
NCI-H226	52.99
NCI-H23	90.93
NCI-H322M	91.84
NCI-H460	102.43
NCI-H522	93.81
**Colon Cancer**	
COLO 205	127.83
HCC-2998	97.04
HCT-116	97.89
HCT-15	86.30
HT29	102.25
KM12	104.98
SW-620	107.69
**CNS Cancer**	
SF-268	85.25
SF-295	78.72
SF-539	86.95
SNB-19	76.43
SNB-75	87.95
U251	87.15
**Melanoma**	
LOX IMVI	87.07
MALME-3M	87.54
M14	93.58
MDA-MB-435	101.68
SK-MEL-2	107.20
SK-MEL-28	102.68
SK-MEL-5	74.78
UACC-257	103.03
UACC-62	88.69
**Ovarian Cancer**	
IGROV1	104.67
OVCAR-3	94.83
OVCAR-4	78.05
OVCAR-5	110.05
OVCAR-8	82.90
NCI/ADR-RES	79.52
SK-OV-3	106.27
**Renal Cancer**	
786-0	101.69
A498	96.72
ACHN	102.39
CAKI-1	83.10
RXF 393	64.85
SN12C	86.51
TK-10	102.55
UO-31	88.04
**Prostate Cancer**	
PC-3	88.86
DU-145	91.37
**Breast Cancer**	
MCF7	84.45
MDA-MB-231/ATCC	95.01
HS 578T	82.55
BT-549	99.72
T-47D	86.82
MDA-MB-468	100.35
Mean	92.94
Delta	39.95
Range	74.84

Mean Growth Percent - Growth Percent

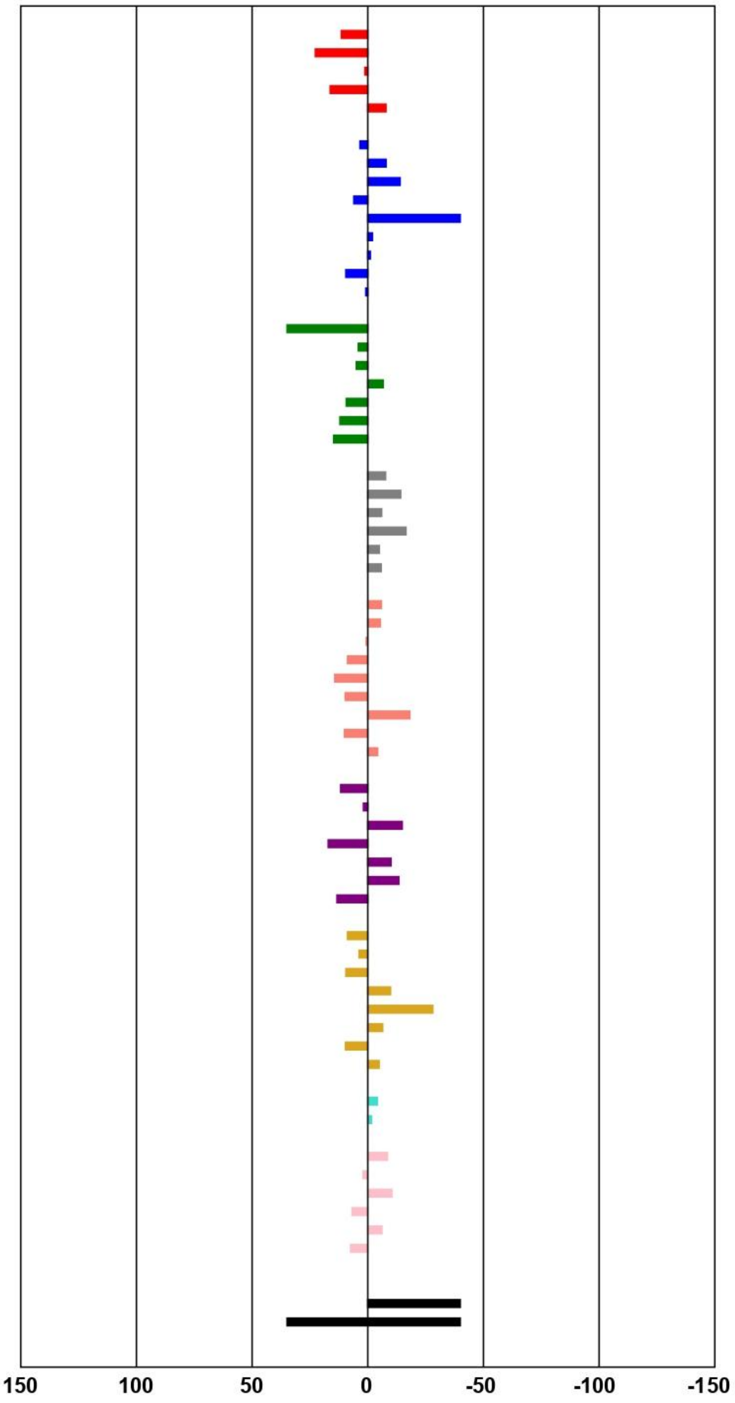

Figure S76. One dose mean graph for compound **8d** at 10 μM

Developmental Therapeutics Program — One Dose Mean Graph	NSC: D-841967 / 1	Conc: 1.00E-5 Molar	Test Date: Feb 06, 2023
	Experiment ID: 2302OS94		Report Date: Mar 13, 2023

Panel/Cell Line	Growth Percent
**Leukemia**	
CCRF-CEM	17.04
HL-60(TB)	5.95
K-562	1.84
MOLT-4	11.41
RPMI-8226	4.30
**Non-Small Cell Lung Cancer**	
A549/ATCC	-2.67
EKVX	-70.75
HOP-62	-31.97
HOP-92	18.25
NCI-H226	-43.03
NCI-H23	-77.13
NCI-H322M	-5.39
NCI-H460	-66.02
NCI-H522	0.98
**Colon Cancer**	
COLO 205	-97.71
HCC-2998	-92.86
HCT-116	-79.66
HCT-15	-92.45
HT29	-58.51
KM12	-78.18
SW-620	-88.30
**CNS Cancer**	
SF-268	19.00
SF-295	-76.67
SF-539	-92.17
SNB-19	-49.31
SNB-75	1.40
U251	-61.99
**Melanoma**	
LOX IMVI	-100.00
MALME-3M	-51.33
M14	-40.55
MDA-MB-435	-34.11
SK-MEL-2	2.80
SK-MEL-28	-76.33
SK-MEL-5	-99.26
UACC-257	39.24
UACC-62	-77.24
**Ovarian Cancer**	
IGROV1	-17.71
OVCAR-3	-85.80
OVCAR-4	7.29
OVCAR-5	-55.96
OVCAR-8	-22.08
NCI/ADR-RES	-31.22
SK-OV-3	34.25
**Renal Cancer**	
786-0	-73.33
A498	-86.77
ACHN	3.15
CAKI-1	8.75
RXF 393	-63.96
SN12C	-78.12
TK-10	37.12
UO-31	-55.24
**Prostate Cancer**	
PC-3	-9.58
DU-145	-84.58
**Breast Cancer**	
MCF7	-70.89
MDA-MB-231/ATCC	-92.56
HS 578T	-8.03
BT-549	-20.18
T-47D	28.57
MDA-MB-468	-26.13
Mean	-38.72
Delta	61.28
Range	139.24

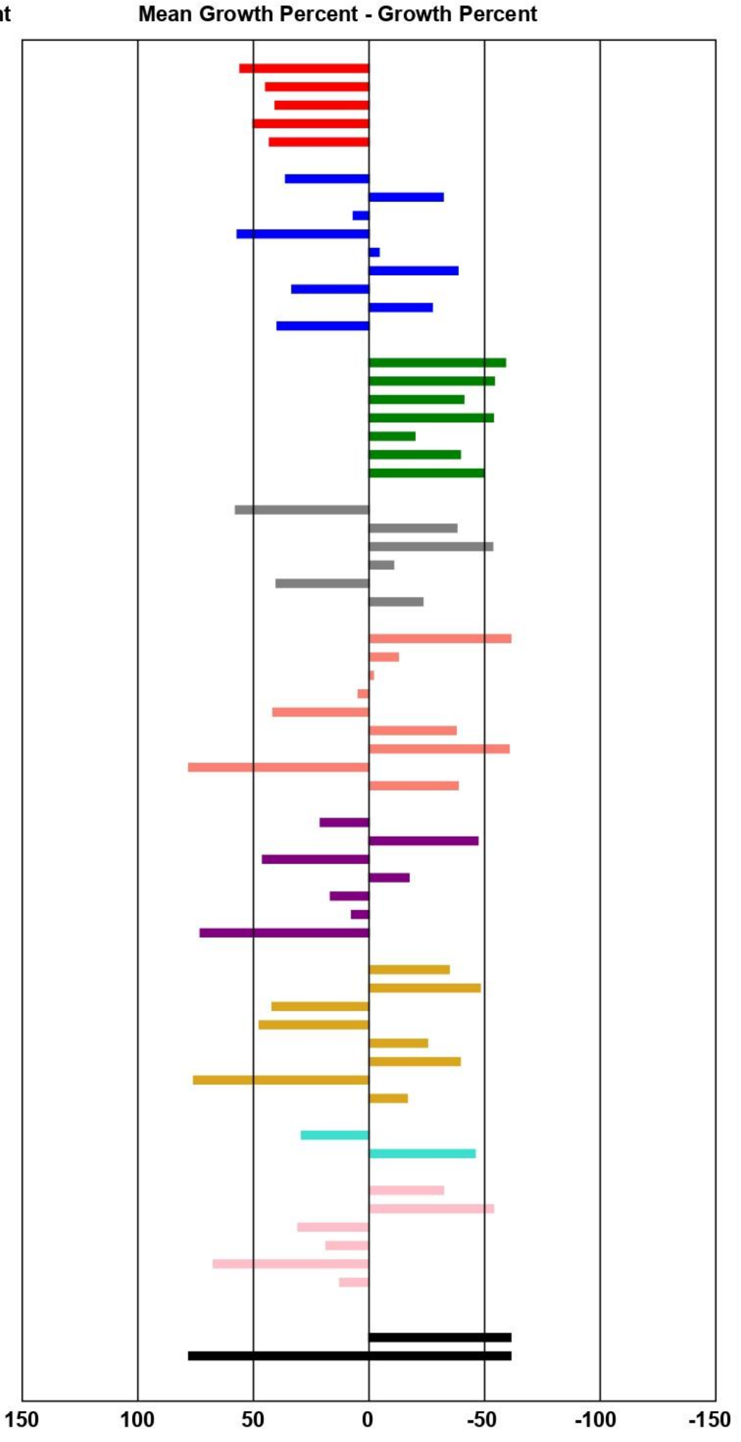

Figure S77. One dose mean graph for compound **8e** at 10 μM

National Cancer Institute Developmental Therapeutics Program
In-Vitro Testing Results

NSC : D - 841967 / 1	Experiment ID : 2307NS43	Test Type : 08	Units : Molar
Report Date : March 16, 2024	Test Date : July 10, 2023	QNS :	MC :
COMI : H1-8f	Stain Reagent : SRB Dual-Pass Related	SSPL : 1AJN	

			Log10 Concentration													
	Time		Mean Optical Densities					Percent Growth								
Panel/Cell Line	Zero	Ctrl	-8.1	-7.1	-6.1	-5.1	-4.1	-8.1	-7.1	-6.1	-5.1	-4.1	GI50	TGI	LC50
**Leukemia**															
CCRF-CEM	0.418	2.115	1.928	2.044	1.266	0.453	0.484	89	96	50	2	4	7.48E-7	> 7.50E-5	> 7.50E-5
HL-60(TB)	0.393	1.484	1.427	1.453	1.057	0.287	0.363	95	97	61	-27	-8	9.96E-7	3.69E-6	> 7.50E-5
K-562	0.218	1.869	1.918	1.947	1.561	0.191	0.338	103	105	81	-13	7	1.62E-6	.	> 7.50E-5
MOLT-4	0.497	1.991	1.979	1.654	1.491	0.444	0.477	99	77	67	-11	-4	1.23E-6	5.46E-6	> 7.50E-5
RPMI-8226	0.736	2.729	2.767	2.845	1.592	0.821	0.839	102	106	43	4	5	5.79E-7	> 7.50E-5	> 7.50E-5
SR	0.438	2.247	2.155	2.161	1.325	0.430	0.428	95	95	49	-2	-2	7.14E-7	6.87E-6	> 7.50E-5
**Non-Small Cell Lung Cancer**															
A549/ATCC	0.509	2.482	2.322	2.375	2.255	0.718	0.071	92	95	88	11	-86	2.34E-6	9.65E-6	3.18E-5
EKVX	0.807	1.880	1.784	1.836	1.627	0.689	0.245	91	96	76	-15	-70	1.46E-6	5.17E-6	3.29E-5
HOP-62	0.734	2.598	2.514	2.584	2.454	1.280	0.462	95	99	92	29	-37	3.52E-6	2.07E-5	> 7.50E-5
HOP-92	1.384	1.790	1.694	1.754	1.652	1.177	0.267	76	91	66	-15	-81	1.18E-6	4.90E-6	2.56E-5
NCI-H226	0.974	1.598	1.510	1.530	1.493	0.968	0.349	86	89	83	0	-64	1.86E-6	7.37E-6	4.49E-5
NCI-H23	0.837	1.956	1.861	1.907	1.693	0.756	0.080	91	96	76	-10	-90	1.52E-6	5.78E-6	2.37E-5
NCI-H322M	0.529	1.843	1.669	1.655	1.502	0.790	0.034	87	86	74	20	-94	2.08E-6	1.12E-5	3.10E-5
NCI-H460	0.284	2.731	2.769	2.793	2.552	0.289	0.123	102	103	93	0	-57	2.17E-6	7.56E-6	5.69E-5
NCI-H522	1.097	2.715	2.521	2.684	2.696	1.120	0.237	88	98	99	1	-78	2.38E-6	7.81E-6	3.30E-5
**Colon Cancer**															
COLO 205	0.517	2.441	2.458	2.344	2.215	0.856	0.239	101	95	88	18	-54	2.61E-6	1.32E-5	6.64E-5
HCC-2998	0.628	1.699	1.665	1.541	1.687	0.231	0.013	97	85	99	-63	-98	1.50E-6	3.05E-6	6.21E-6
HCT-116	0.186	1.731	1.779	1.817	1.680	0.107	0.143	103	106	97	-42	-23	1.62E-6	3.71E-6	> 7.50E-5
HCT-15	0.301	1.799	1.769	1.794	1.442	0.180	0.006	98	100	76	-40	-98	1.26E-6	3.38E-6	1.10E-5
HT29	0.324	2.106	2.062	2.185	1.974	0.403	0.051	98	104	93	4	-84	2.28E-6	8.41E-6	3.08E-5
KM12	0.481	2.487	2.446	2.506	2.202	0.345	0.032	98	101	86	-28	-93	1.54E-6	4.23E-6	1.61E-5
SW-620	0.294	2.259	2.162	2.209	1.665	1.241	0.922	95	97	70	48	32	6.19E-6	> 7.50E-5	> 7.50E-5
**CNS Cancer**															
SF-268	1.122	2.923	2.832	3.001	2.972	1.590	0.349	95	104	103	26	-69	3.65E-6	1.41E-5	4.74E-5
SF-295	0.657	2.153	1.990	2.087	1.117	0.398	0.094	89	96	31	-39	-86	3.78E-7	2.06E-6	1.27E-5
SF-539	0.796	2.461	2.403	2.329	1.567	0.482	0.029	97	92	46	-40	-96	6.23E-7	2.60E-6	1.15E-5
SNB-75	1.106	2.007	1.829	1.822	1.481	1.070	0.380	80	79	42	-3	-66	4.49E-7	6.34E-6	4.21E-5
U251	0.366	1.967	1.927	1.896	1.246	0.242	0.045	97	96	55	-34	-88	8.52E-7	3.11E-6	1.49E-5
**Melanoma**															
LOX IMVI	0.434	2.259	2.088	2.109	1.959	0.234	0.004	91	92	84	-46	-99	1.36E-6	3.31E-6	8.89E-6
MALME-3M	0.419	1.053	0.956	1.007	0.915	0.549	0.106	85	93	78	20	-75	2.31E-6	1.23E-5	4.12E-5
M14	0.546	2.300	2.383	2.282	2.042	0.614	0.270	105	99	85	4	-51	2.03E-6	8.84E-6	7.33E-5
MDA-MB-435	0.524	2.366	2.304	2.319	2.208	0.843	0.091	97	97	91	17	-83	2.71E-6	1.12E-5	3.53E-5
SK-MEL-2	1.307	3.085	3.060	3.098	2.930	1.552	0.040	99	101	91	14	-97	2.56E-6	9.99E-6	2.83E-5
SK-MEL-28	0.650	1.956	1.961	2.037	1.562	0.936	0.018	100	106	70	22	-97	1.94E-6	1.14E-5	3.01E-5
SK-MEL-5	0.961	2.899	2.744	2.881	2.794	0.476	-0.001	92	99	95	-50	-100	1.52E-6	3.37E-6	7.44E-6
UACC-257	1.082	2.663	2.522	2.559	2.455	1.796	0.145	91	93	87	45	-87	5.74E-6	1.65E-5	3.96E-5
UACC-62	0.762	2.302	2.216	2.296	2.077	0.950	0.028	94	100	85	12	-96	2.28E-6	9.72E-6	2.81E-5
**Ovarian Cancer**															
IGROV1	0.228	1.376	1.250	1.290	1.109	0.363	0.053	89	92	77	12	-77	1.93E-6	1.02E-5	3.72E-5
OVCAR-3	0.589	2.046	2.019	2.134	1.873	0.227	0.031	98	106	88	-62	-95	1.35E-6	2.91E-6	6.28E-6
OVCAR-4	0.909	2.128	2.096	2.165	2.037	1.419	0.239	97	103	92	42	-74	5.18E-6	1.73E-5	4.68E-5
OVCAR-5	0.511	1.441	1.472	1.441	1.409	0.648	0.011	103	100	97	15	-98	2.78E-6	1.01E-5	2.81E-5
OVCAR-8	0.699	2.802	2.781	2.828	2.676	0.949	0.042	99	101	94	12	-94	2.58E-6	9.71E-6	2.88E-5
NCI/ADR-RES	0.445	1.339	1.312	1.372	1.233	0.397	0.034	97	104	88	-11	-92	1.82E-6	5.84E-6	2.27E-5
SK-OV-3	1.032	2.524	2.470	2.605	2.514	1.501	0.251	96	105	99	31	-76	4.00E-6	1.47E-5	4.32E-5
**Renal Cancer**															
786-0	0.909	3.028	3.006	2.988	2.085	0.418	0.294	99	98	55	-54	-68	8.41E-7	2.41E-6	6.88E-6
A498	1.400	2.517	2.417	2.435	2.325	1.467	0.229	91	93	83	6	-84	2.01E-6	8.75E-6	3.16E-5
ACHN	0.301	1.478	1.517	1.502	1.059	0.376	0.008	103	102	64	6	-97	1.33E-6	8.63E-6	2.62E-5
CAKI-1	0.799	2.704	2.585	2.639	2.205	1.001	0.097	94	97	74	11	-88	1.79E-6	9.61E-6	3.09E-5
RXF 393	0.921	1.484	1.370	1.388	1.011	0.736	0.165	80	83	16	-20	-82	2.33E-7	2.08E-6	2.28E-5
SN12C	0.640	2.208	2.123	2.126	1.992	0.780	0.041	95	95	86	9	-94	2.21E-6	9.16E-6	2.81E-5
UO-31	0.571	1.935	1.733	1.704	1.559	0.662	0.011	85	83	72	7	-98	1.65E-6	8.68E-6	2.61E-5
**Prostate Cancer**															
PC-3	0.621	2.033	2.018	1.969	1.654	0.590	0.216	99	95	73	-5	-65	1.48E-6	6.46E-6	4.18E-5
DU-145	0.467	2.022	2.049	2.078	1.865	0.384	0.013	102	104	90	-18	-97	1.76E-6	5.12E-6	1.91E-5
**Breast Cancer**															
MCF7	0.336	1.759	1.628	1.619	1.354	0.230	0.036	91	90	72	-32	-89	1.21E-6	3.70E-6	1.56E-5
MDA-MB-231/ATCC	0.524	1.168	1.172	1.185	1.084	0.465	0.071	101	103	87	-11	-87	1.78E-6	5.76E-6	2.45E-5
HS 578T	1.160	2.309	2.310	2.321	2.075	0.421	0.075	100	101	80	-64	-94	1.21E-6	2.69E-6	6.01E-6
BT-549	1.006	1.957	1.890	2.094	1.959	1.298	0.439	93	114	100	31	-56	3.95E-6	1.69E-5	6.34E-5
T-47D	0.718	2.108	1.906	2.110	2.205	1.209	0.483	85	100	107	35	-33	4.68E-6	2.48E-5	> 7.50E-5
MDA-MB-468	0.720	1.101	1.060	1.101	1.044	0.615	0.058	89	100	85	-15	-92	1.68E-6	5.34E-6	2.15E-5

Figure S78. Values of log molar concentration of response parameters ($\log_{10}$ GI_{50}, $\log_{10}$ TGI & $\log_{10}$ LC_{50}) for compound **8e**

Supporting Information

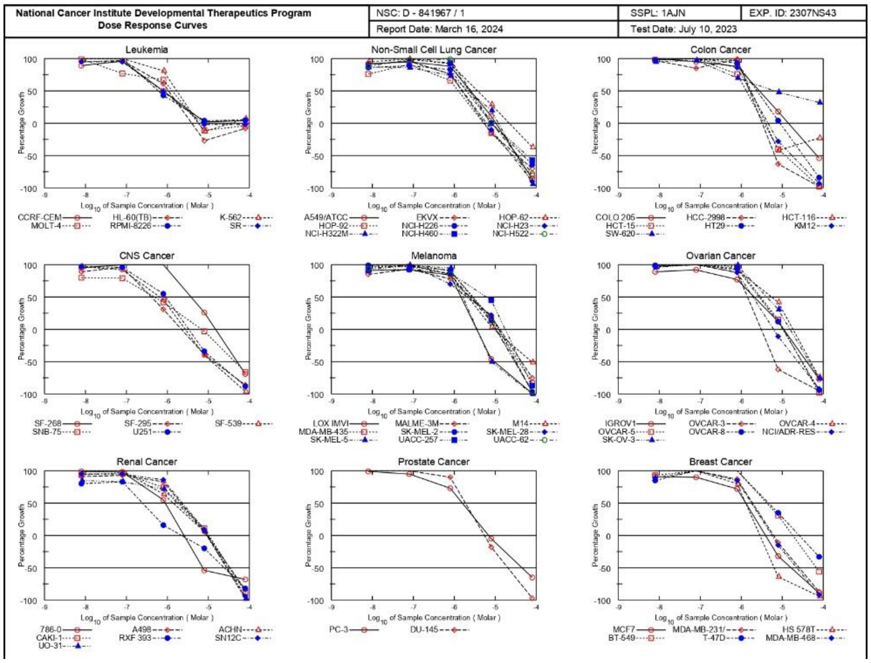

Figure S79. Dose-response curves (% growth versus sample concentration) for all cell lines with different subpanel obtained from the NCI's in vitro disease-oriented human cancer cells line for compound **8e** on nine types of cancer

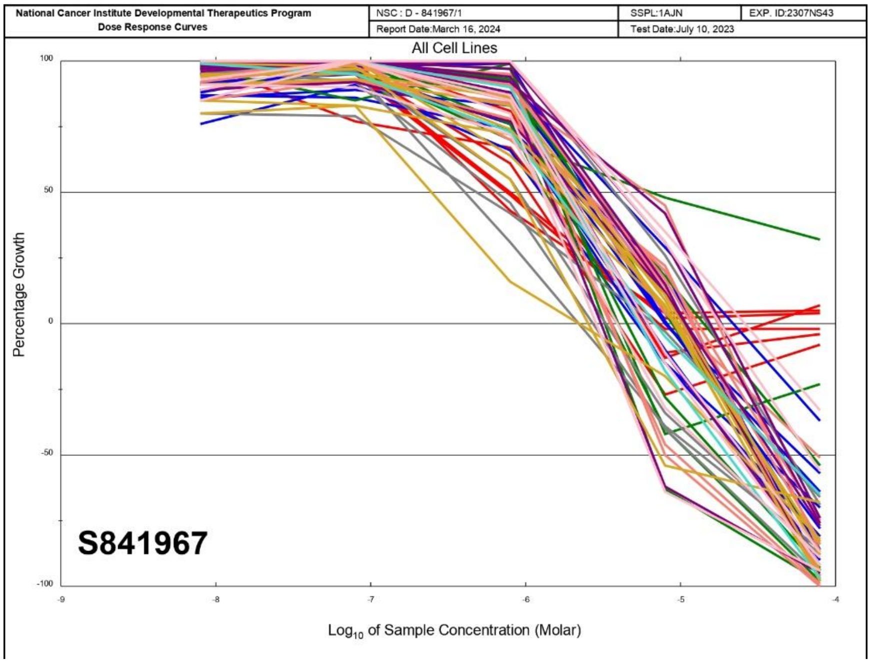

Figure S80. Dose-response curves for all cell lines in the NCI60 panel exposed compound **8e** with tissue originated colors and shapes.

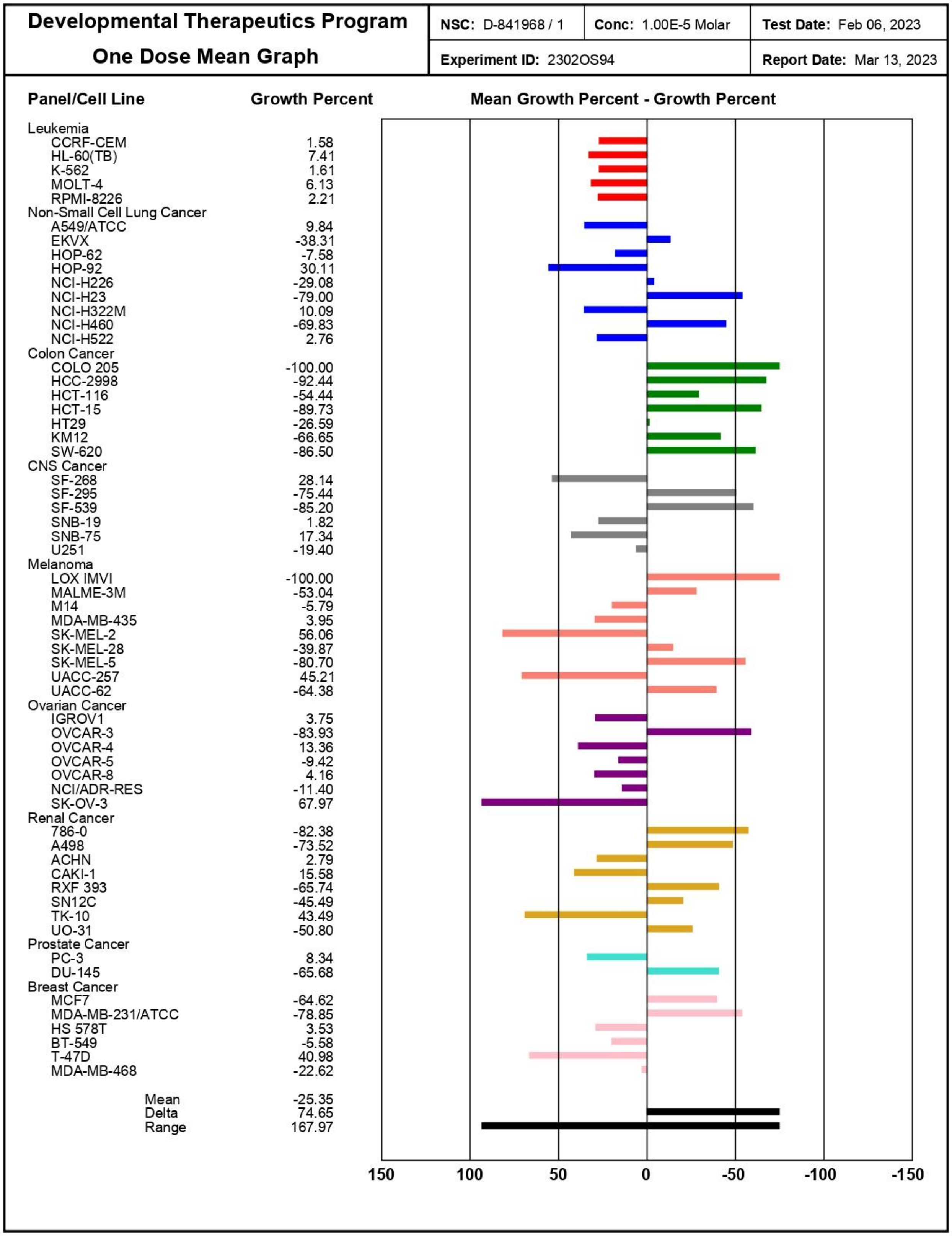

Figure S81. One dose mean graph for compound **8f** at 10 μM

National Cancer Institute Developmental Therapeutics Program In-Vitro Testing Results			
NSC : D - 841968 / 1	Experiment ID : 2307NS43	Test Type : 08	Units : Molar
Report Date : March 16, 2024	Test Date : July 10, 2023	QNS :	MC :
COMI : H1-8g	Stain Reagent : SRB Dual-Pass Related	SSPL : 1AJN	

Panel/Cell Line	Time Zero	Ctrl	Mean Optical Densities -8.0	-7.0	-6.0	-5.0	-4.0	Percent Growth -8.0	-7.0	-6.0	-5.0	-4.0	GI50	TGI	LC50
**Leukemia**															
CCRF-CEM	0.418	1.972	1.880	1.823	1.643	0.485	0.476	94	90	79	4	4	2.44E-6	> 1.00E-4	> 1.00E-4
HL-60(TB)	0.393	1.489	1.765	1.737	1.754	0.555	0.435	125	123	124	15	4	4.77E-6	> 1.00E-4	> 1.00E-4
K-562	0.218	1.825	2.075	2.032	1.916	0.335	0.373	116	113	106	7	10	3.68E-6	> 1.00E-4	> 1.00E-4
MOLT-4	0.497	1.870	2.019	2.058	1.666	0.829	0.638	111	114	85	24	10	3.77E-6	> 1.00E-4	> 1.00E-4
RPMI-8226	0.736	2.776	2.795	2.769	2.756	1.012	1.000	101	100	99	14	13	3.74E-6	> 1.00E-4	> 1.00E-4
SR	0.438	2.247	2.371	2.136	1.955	0.567	0.674	107	94	84	7	13	2.76E-6	> 1.00E-4	> 1.00E-4
**Non-Small Cell Lung Cancer**															
A549/ATCC	0.509	2.552	2.492	2.438	2.501	0.639	0.167	97	94	97	6	-67	3.32E-6	1.22E-5	5.82E-5
EKVX	0.807	1.882	1.808	1.800	1.867	0.760	0.201	93	92	99	-6	-75	2.92E-6	8.79E-6	4.34E-5
HOP-62	0.734	2.367	2.266	2.171	2.209	0.581	0.236	94	88	90	-21	-68	2.30E-6	6.49E-6	4.16E-5
HOP-92	1.384	1.878	1.775	1.744	1.840	1.281	0.244	79	73	92	-7	-82	2.65E-6	8.41E-6	3.69E-5
NCI-H226	0.974	1.664	1.602	1.528	1.478	0.843	0.260	91	80	73	-14	-73	1.84E-6	6.98E-6	4.07E-5
NCI-H23	0.837	1.962	1.903	1.887	1.892	0.764	0.059	95	93	94	-9	-93	2.68E-6	8.22E-6	3.09E-5
NCI-H322M	0.529	1.909	1.886	1.869	1.830	0.783	0.141	98	97	94	18	-73	3.83E-6	1.59E-5	5.56E-5
NCI-H460	0.284	2.801	2.830	2.804	2.781	0.133	0.129	101	100	99	-53	-55	2.10E-6	4.47E-6	9.51E-6
NCI-H522	1.097	2.769	2.716	2.566	2.581	0.983	0.528	97	88	89	-10	-52	2.46E-6	7.86E-6	9.01E-5
**Colon Cancer**															
COLO 205	0.517	2.233	2.254	2.342	2.282	0.151	0.141	101	106	103	-71	-73	2.02E-6	3.91E-6	7.59E-6
HCC-2998	0.628	1.794	1.739	1.780	1.715	0.163	0.017	95	99	93	-74	-97	1.81E-6	3.61E-6	7.18E-6
HCT-116	0.186	1.792	1.721	1.673	1.526	0.094	0.149	96	93	83	-49	-20	1.78E-6	4.24E-6	> 1.00E-4
HCT-15	0.301	1.883	1.751	1.683	1.588	0.135	0.006	92	87	81	-55	-98	1.70E-6	3.94E-6	9.17E-6
HT29	0.324	2.361	2.146	2.153	1.941	0.295	0.129	89	90	79	-9	-60	2.15E-6	7.92E-6	6.33E-5
KM12	0.481	2.547	2.510	2.613	2.494	0.211	0.089	98	103	97	-56	-81	2.04E-6	4.31E-6	9.12E-6
SW-620	0.294	2.348	2.278	2.259	2.223	1.377	1.014	97	96	94	53	35	1.42E-5	> 1.00E-4	> 1.00E-4
**CNS Cancer**															
SF-268	1.122	3.036	2.969	2.956	2.925	1.661	0.283	97	96	94	28	-75	4.67E-6	1.88E-5	5.75E-5
SF-295	0.657	2.209	2.030	1.958	2.082	0.359	0.044	88	84	92	-45	-93	2.02E-6	4.67E-6	1.25E-5
SF-539	0.796	2.535	2.473	2.430	2.388	0.226	0.029	96	94	92	-72	-96	1.80E-6	3.64E-6	7.37E-6
SNB-75	1.106	2.010	1.827	1.701	1.806	1.038	0.218	80	66	77	-6	-80	2.13E-6	8.44E-6	3.90E-5
U251	0.366	2.074	2.038	1.925	2.003	0.247	0.114	98	91	96	-33	-69	2.28E-6	5.58E-6	3.03E-5
**Melanoma**															
LOX IMVI	0.434	2.329	2.198	2.197	2.198	0.014	0.002	93	93	93	-97	-100	1.69E-6	3.09E-6	5.66E-6
MALME-3M	0.419	1.042	1.154	1.201	1.065	0.390	0.189	118	125	104	-7	-55	3.05E-6	8.64E-6	7.90E-5
M14	0.546	2.362	2.294	2.318	2.248	0.416	0.305	96	98	94	-24	-44	2.36E-6	6.27E-6	> 1.00E-4
MDA-MB-435	0.524	2.340	2.134	2.218	2.192	0.291	0.102	89	93	92	-44	-81	2.03E-6	4.72E-6	1.42E-5
SK-MEL-2	1.307	3.146	3.114	3.123	3.107	1.666	0.268	98	99	98	19	-79	4.08E-6	1.57E-5	5.04E-5
SK-MEL-28	0.650	1.988	2.033	1.961	1.958	0.901	0.024	103	98	98	19	-96	4.02E-6	1.46E-5	3.96E-5
SK-MEL-5	0.961	2.947	2.888	2.876	2.879	1.304	0.006	97	96	97	17	-99	3.87E-6	1.41E-5	3.77E-5
UACC-257	1.082	2.678	2.617	2.628	2.608	1.916	0.491	96	97	96	52	-55	1.05E-5	3.08E-5	9.04E-5
UACC-62	0.762	2.407	2.317	2.243	2.186	0.868	0.030	95	90	87	6	-96	2.86E-6	1.16E-5	3.55E-5
**Ovarian Cancer**															
IGROV1	0.228	1.368	1.412	1.497	1.468	0.473	0.171	104	111	109	21	-25	4.71E-6	2.88E-5	> 1.00E-4
OVCAR-3	0.589	2.160	2.180	2.133	2.111	0.128	0.048	101	98	97	-78	-92	1.85E-6	3.57E-6	6.90E-6
OVCAR-4	0.909	2.169	2.087	2.046	2.012	1.287	0.202	94	90	88	30	-78	4.49E-6	1.90E-5	5.52E-5
OVCAR-5	0.511	1.512	1.528	1.437	1.384	0.544	0.006	102	93	87	3	-99	2.78E-6	1.08E-5	3.32E-5
OVCAR-8	0.699	2.884	2.860	2.845	2.810	0.858	0.062	99	98	97	7	-91	3.32E-6	1.19E-5	3.82E-5
NCI/ADR-RES	0.445	1.460	1.452	1.409	1.302	0.361	0.026	99	95	84	-19	-94	2.15E-6	6.55E-6	2.58E-5
SK-OV-3	1.032	2.166	2.308	2.276	2.194	1.442	0.143	112	110	102	36	-86	6.17E-6	1.97E-5	5.06E-5
**Renal Cancer**															
786-0	0.909	3.050	3.033	2.985	3.008	0.261	0.376	99	97	98	-71	-59	1.92E-6	3.79E-6	7.48E-6
A498	1.400	2.417	2.336	2.403	2.447	0.429	0.197	92	99	103	-69	-86	2.03E-6	3.96E-6	7.72E-6
ACHN	0.301	1.527	1.545	1.478	1.450	0.378	0.007	101	96	94	6	-98	3.16E-6	1.15E-5	3.48E-5
CAKI-1	0.799	2.696	2.589	2.592	2.498	1.081	0.060	94	95	90	15	-92	3.38E-6	1.37E-5	4.02E-5
RXF 393	0.921	1.479	1.410	1.409	1.423	0.420	0.178	88	88	90	-54	-81	1.89E-6	4.20E-6	9.32E-6
SN12C	0.640	2.360	2.258	2.194	2.059	0.333	0.050	94	90	83	-48	-92	1.77E-6	4.29E-6	1.11E-5
UO-31	0.571	1.942	1.767	1.726	1.763	0.406	0.026	87	84	87	-29	-95	2.08E-6	5.63E-6	2.08E-5
**Prostate Cancer**															
PC-3	0.621	1.970	1.950	1.910	1.925	0.695	0.278	98	96	97	5	-55	3.25E-6	1.23E-5	8.18E-5
DU-145	0.467	2.040	2.082	2.014	1.982	0.299	0.032	103	98	96	-36	-93	2.24E-6	5.34E-6	1.75E-5
**Breast Cancer**															
MCF7	0.336	1.767	1.638	1.633	1.531	0.193	0.047	91	91	83	-43	-86	1.84E-6	4.59E-6	1.48E-5
MDA-MB-231/ATCC	0.524	1.204	1.216	1.168	1.202	0.209	0.072	102	95	100	-60	-86	2.05E-6	4.21E-6	8.64E-6
HS 578T	1.160	2.355	2.385	2.381	2.373	0.242	0.254	103	102	102	-79	-78	1.93E-6	3.65E-6	6.90E-6
BT-549	1.006	1.917	1.845	1.870	1.922	0.759	0.582	92	95	101	-25	-42	2.53E-6	6.36E-6	> 1.00E-4
T-47D	0.718	1.868	1.774	1.761	1.726	0.942	0.324	92	91	88	19	-55	3.57E-6	1.83E-5	8.60E-5
MDA-MB-468	0.720	1.129	1.100	1.078	1.143	0.667	0.089	93	87	103	-7	-88	3.03E-6	8.58E-6	3.39E-5

Log10 Concentration

Figure S82. Values of log molar concentration of response parameters ($\log_{10}$ GI_{50}, $\log_{10}$ TGI & $\log_{10}$ LC_{50}) for compound **8f**

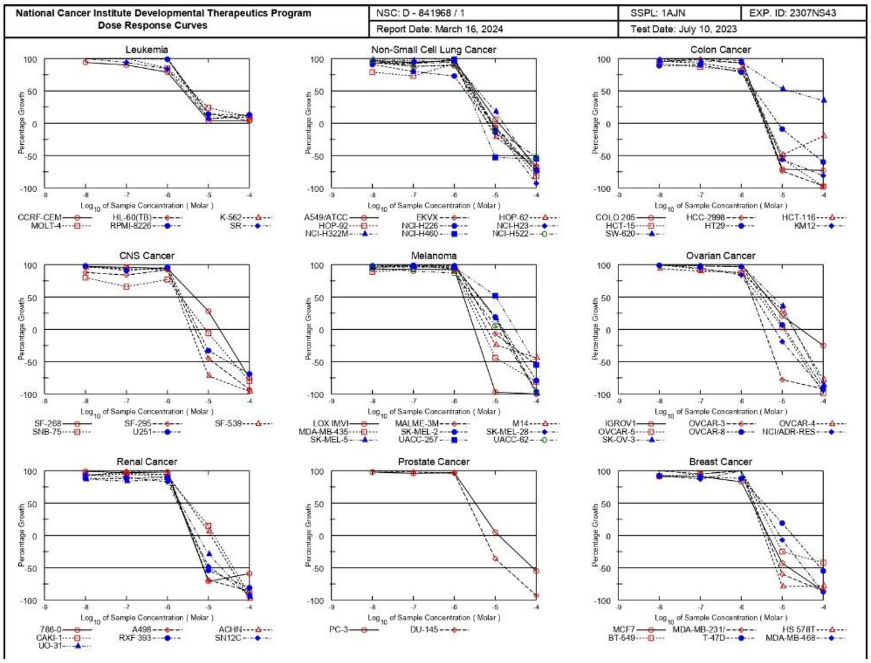

Figure S83. Dose-response curves (% growth versus sample concentration) for all cell lines with different subpanel obtained from the NCI's in vitro disease-oriented human cancer cells line for compound **8f** on nine types of cancer

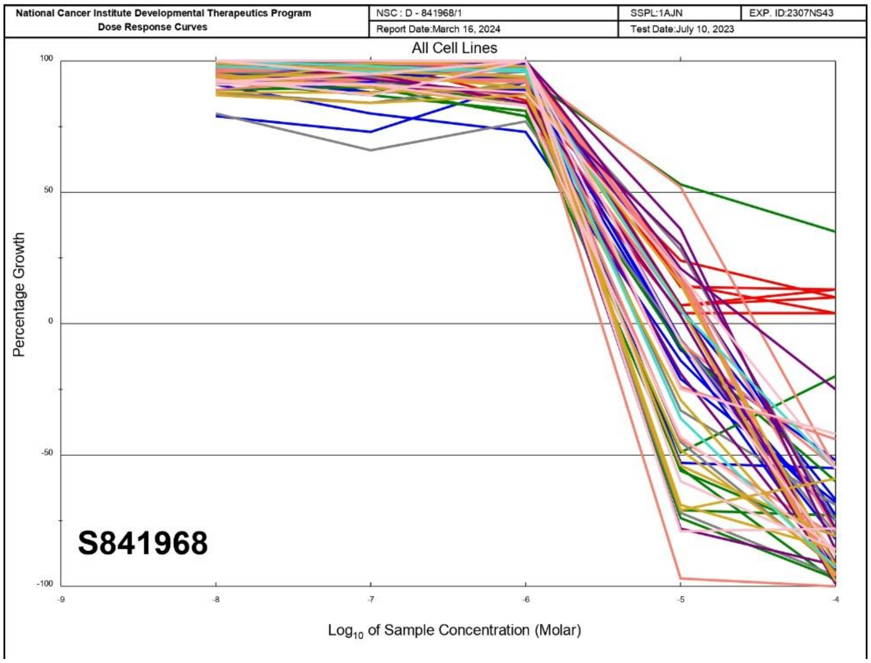

Figure S84. Dose-response curves for all cell lines in the NCI60 panel exposed compound **8f** with tissue originated colors and shapes.

Developmental Therapeutics Program One Dose Mean Graph	NSC: D-841969 / 1	Conc: 1.00E-5 Molar	Test Date: Feb 06, 2023
	Experiment ID: 2302OS94		Report Date: Mar 13, 2023

Panel/Cell Line	Growth Percent
Leukemia	
CCRF-CEM	97.34
HL-60(TB)	99.26
K-562	89.97
MOLT-4	93.95
RPMI-8226	91.37
Non-Small Cell Lung Cancer	
A549/ATCC	98.07
EKVX	71.81
HOP-62	88.25
HOP-92	110.54
NCI-H226	80.64
NCI-H23	80.58
NCI-H322M	93.43
NCI-H460	113.37
NCI-H522	95.39
Colon Cancer	
COLO 205	122.53
HCC-2998	102.16
HCT-116	93.17
HCT-15	93.22
HT29	118.74
KM12	103.49
SW-620	109.42
CNS Cancer	
SF-268	113.29
SF-295	90.99
SF-539	88.09
SNB-19	89.64
SNB-75	113.55
U251	93.22
Melanoma	
LOX IMVI	89.83
MALME-3M	90.55
M14	109.34
MDA-MB-435	105.93
SK-MEL-2	111.15
SK-MEL-28	107.04
SK-MEL-5	86.52
UACC-257	108.74
UACC-62	78.77
Ovarian Cancer	
IGROV1	99.03
OVCAR-3	111.25
OVCAR-4	110.21
OVCAR-5	99.15
OVCAR-8	92.95
NCI/ADR-RES	92.71
SK-OV-3	110.61
Renal Cancer	
786-0	100.17
A498	123.79
ACHN	90.90
CAKI-1	83.94
RXF 393	69.51
SN12C	90.83
TK-10	114.82
UO-31	71.70
Prostate Cancer	
PC-3	90.76
DU-145	117.62
Breast Cancer	
MCF7	84.90
MDA-MB-231/ATCC	78.24
HS 578T	90.61
BT-549	89.70
T-47D	92.44
MDA-MB-468	101.05
Mean	97.12
Delta	27.61
Range	54.28

Mean Growth Percent - Growth Percent

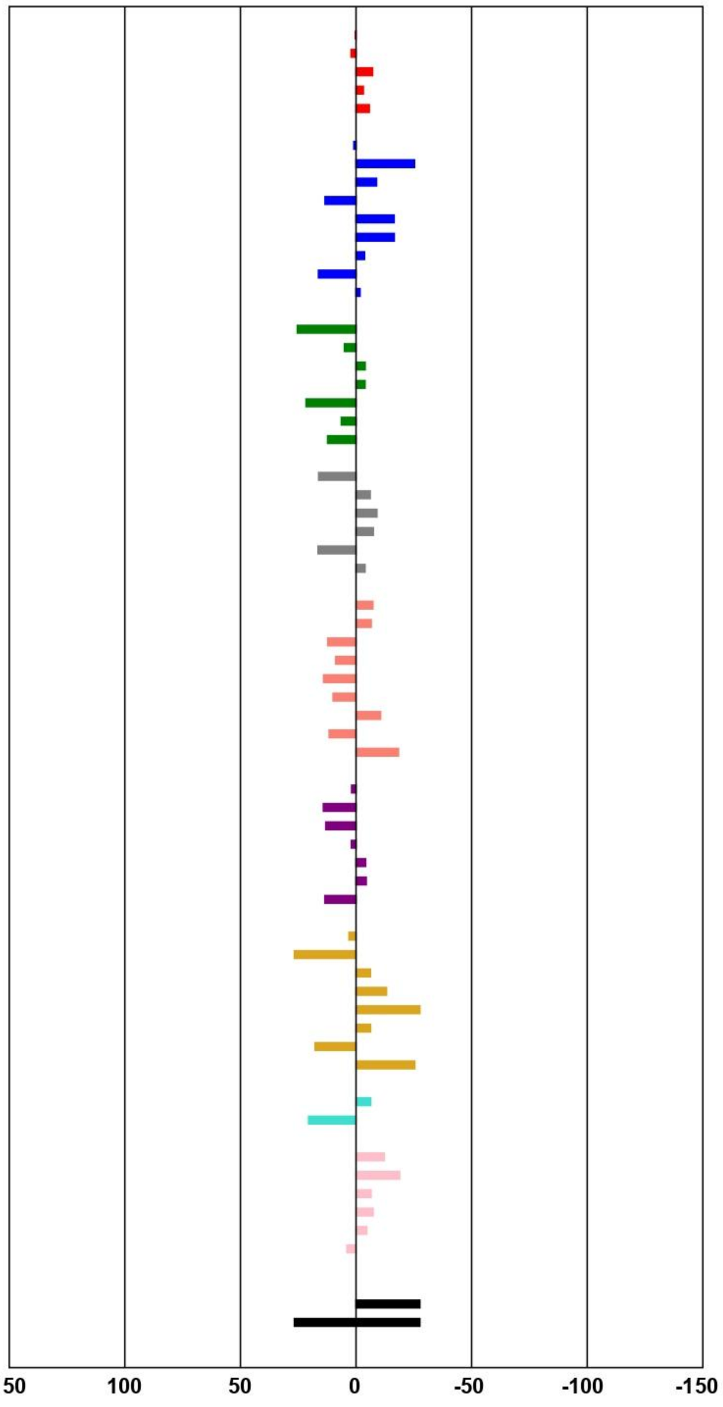

Figure S85. One dose mean graph for compound **8g** at 10 µM

Developmental Therapeutics Program One Dose Mean Graph	NSC: D-844211 / 1	Conc: 1.00E-5 Molar	Test Date: Apr 10, 2023
	Experiment ID: 2304OS17		Report Date: Mar 16, 2024

Panel/Cell Line	Growth Percent
**Leukemia**	
CCRF-CEM	37.33
HL-60(TB)	82.19
K-562	27.00
MOLT-4	33.17
RPMI-8226	56.74
SR	31.54
**Non-Small Cell Lung Cancer**	
A549/ATCC	61.69
EKVX	30.63
HOP-62	78.80
NCI-H226	58.17
NCI-H23	48.09
NCI-H322M	81.42
NCI-H460	25.71
NCI-H522	-32.71
**Colon Cancer**	
COLO 205	63.29
HCC-2998	30.93
HCT-116	56.17
HCT-15	44.35
HT29	59.62
KM12	31.82
SW-620	63.08
**CNS Cancer**	
SF-268	59.41
SF-295	65.60
SF-539	74.75
SNB-19	60.02
SNB-75	75.18
U251	62.05
**Melanoma**	
LOX IMVI	51.71
MALME-3M	42.07
M14	69.90
MDA-MB-435	56.72
SK-MEL-2	60.45
SK-MEL-28	43.18
SK-MEL-5	70.97
UACC-257	76.75
UACC-62	81.89
**Ovarian Cancer**	
IGROV1	73.65
OVCAR-3	91.02
OVCAR-4	75.37
OVCAR-5	85.94
OVCAR-8	72.76
NCI/ADR-RES	71.39
SK-OV-3	75.80
**Renal Cancer**	
786-0	59.88
A498	46.90
ACHN	64.92
CAKI-1	46.42
RXF 393	29.30
SN12C	52.49
TK-10	131.04
UO-31	67.27
**Prostate Cancer**	
DU-145	58.45
**Breast Cancer**	
MCF7	44.13
MDA-MB-231/ATCC	61.06
HS 578T	48.08
BT-549	59.92
T-47D	34.79
MDA-MB-468	65.92
Mean	57.52
Delta	90.23
Range	163.75

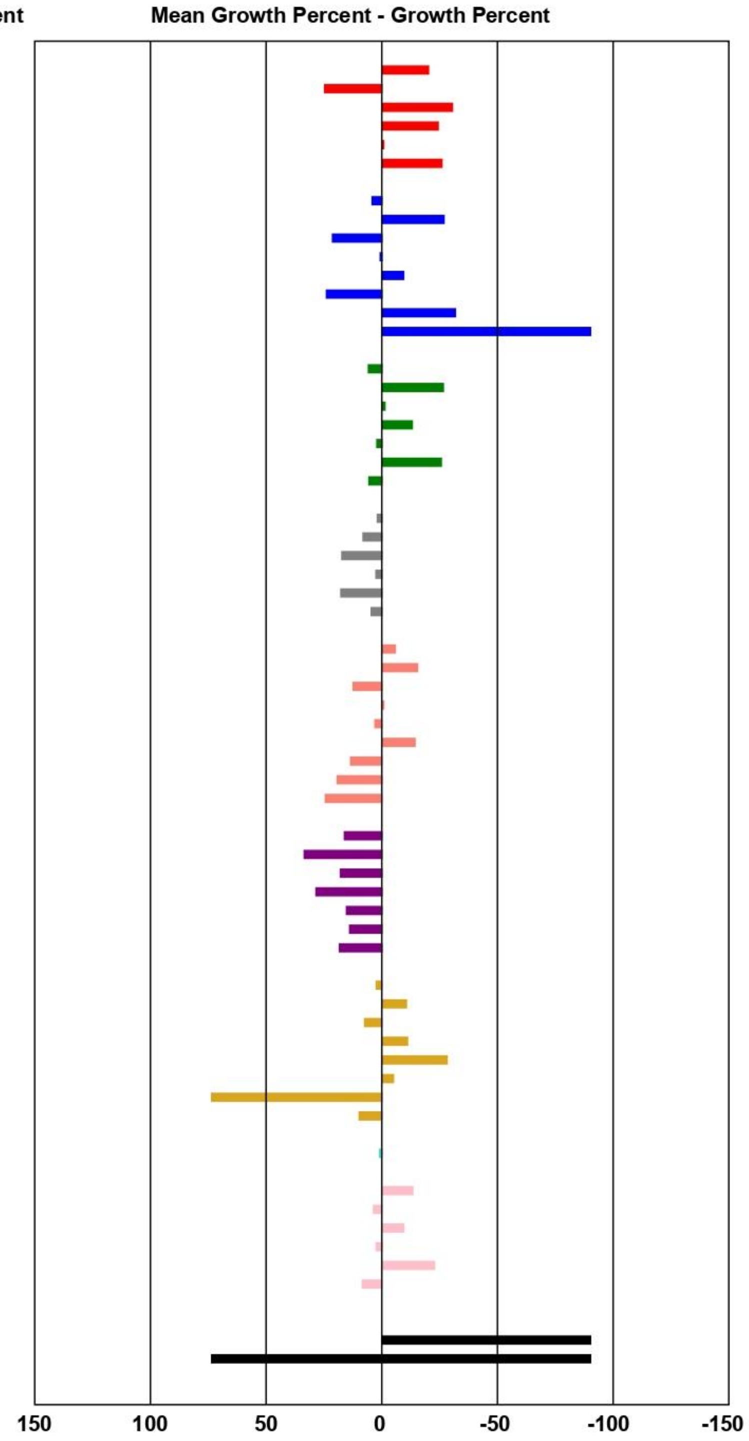

Figure S86. One dose mean graph for compound **10a** at 10 μM

Developmental Therapeutics Program One Dose Mean Graph	NSC: D-844212 / 1	Conc: 1.00E-5 Molar	Test Date: Apr 10, 2023
	Experiment ID: 2304OS17		Report Date: Apr 02, 2025

Panel/Cell Line	Growth Percent
**Leukemia**	
CCRF-CEM	-34.39
HL-60(TB)	-11.62
K-562	3.03
MOLT-4	-10.81
RPMI-8226	-21.55
SR	-15.37
**Non-Small Cell Lung Cancer**	
A549/ATCC	12.86
EKVX	-24.62
HOP-62	-32.36
NCI-H226	-34.60
NCI-H23	-74.99
NCI-H322M	27.83
NCI-H460	-62.49
NCI-H522	-55.13
**Colon Cancer**	
COLO 205	-75.78
HCC-2998	-90.92
HCT-116	2.12
HCT-15	-84.54
HT29	-52.12
KM12	-79.01
SW-620	-72.40
**CNS Cancer**	
SF-268	12.41
SF-295	-66.23
SF-539	-41.81
SNB-19	-19.24
SNB-75	30.56
U251	-64.06
**Melanoma**	
LOX IMVI	-92.78
MALME-3M	-61.54
M14	2.61
MDA-MB-435	-19.95
SK-MEL-2	-43.54
SK-MEL-28	-24.32
SK-MEL-5	13.26
UACC-257	40.57
UACC-62	34.08
**Ovarian Cancer**	
IGROV1	-1.46
OVCAR-3	-58.11
OVCAR-4	18.09
OVCAR-5	-18.72
OVCAR-8	-1.89
NCI/ADR-RES	-35.90
SK-OV-3	24.74
**Renal Cancer**	
786-0	11.48
A498	-23.82
ACHN	-13.73
CAKI-1	-24.52
RXF 393	-47.51
SN12C	-96.49
TK-10	50.39
UO-31	-54.53
**Prostate Cancer**	
DU-145	-21.11
**Breast Cancer**	
MCF7	-33.26
MDA-MB-231/ATCC	-83.22
HS 578T	-24.34
BT-549	17.97
T-47D	-4.98
MDA-MB-468	-14.58
Mean	-26.25
Delta	70.24
Range	146.88

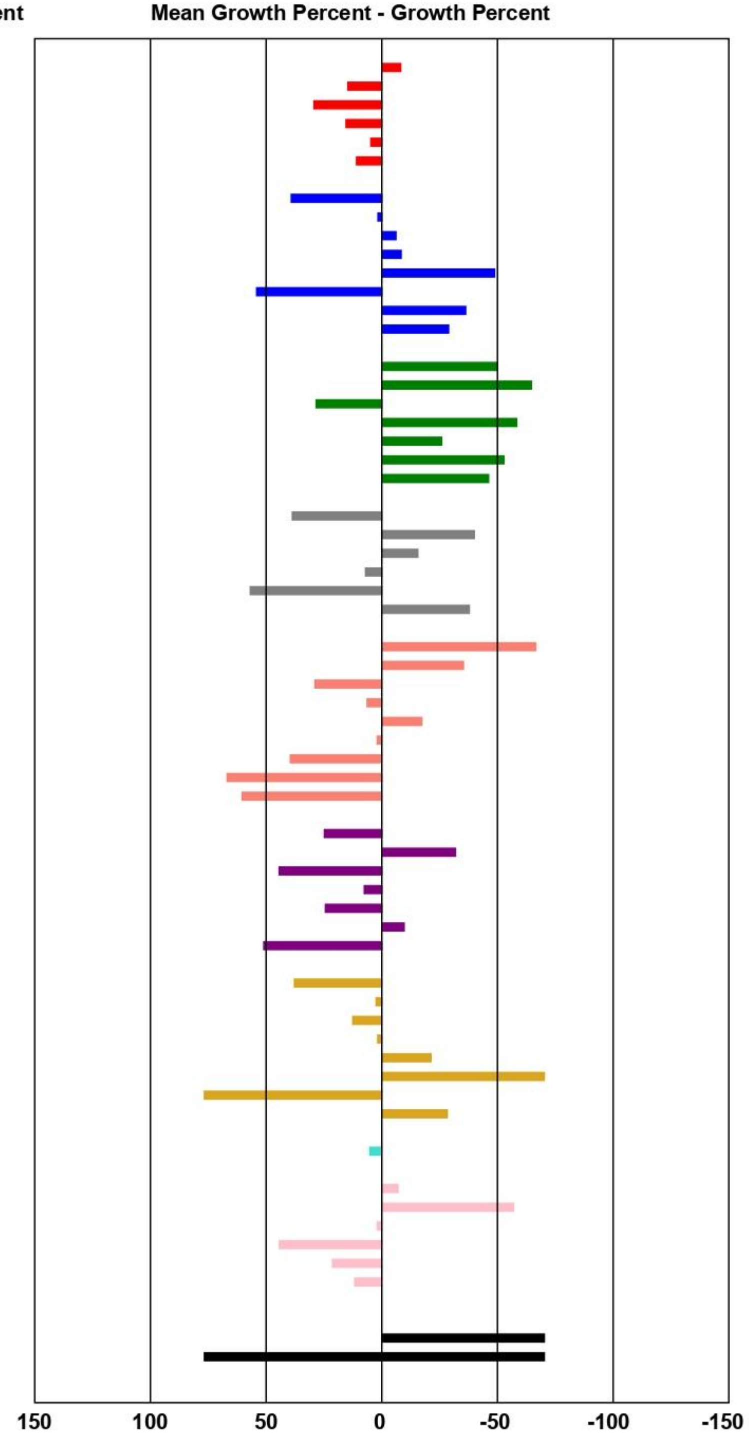

Figure S87. One dose mean graph for compound **10b** at 10 μM

National Cancer Institute Developmental Therapeutics Program
In-Vitro Testing Results

NSC : D - 844212 / 1	Experiment ID : 2309NS73	Test Type : 08	Units : Molar
Report Date : March 16, 2024	Test Date : September 11, 2023	QNS :	MC :
COMI : H1-10b	Stain Reagent : SRB Dual-Pass Related	SSPL : 1AJN	

			Log10 Concentration												
	Time		Mean Optical Densities					Percent Growth							
Panel/Cell Line	Zero	Ctrl	-8.0	-7.0	-6.0	-5.0	-4.0	-8.0	-7.0	-6.0	-5.0	-4.0	GI50	TGI	LC50
Leukemia															
CCRF-CEM	0.517	2.849	2.824	2.784	2.806	1.028	1.052	99	97	98	22	23	4.28E-6	> 1.00E-4	> 1.00E-4
HL-60(TB)	0.600	2.790	2.687	2.660	2.666	0.907	0.577	95	94	94	14	-4	3.56E-6	6.10E-5	> 1.00E-4
K-562	0.212	2.210	2.206	2.077	1.970	0.470	0.351	100	93	88	13	7	3.21E-6	> 1.00E-4	> 1.00E-4
MOLT-4	0.451	2.361	2.339	2.380	2.098	0.767	0.604	99	101	86	17	8	3.31E-6	> 1.00E-4	> 1.00E-4
RPMI-8226	0.732	2.678	2.673	2.633	2.645	1.155	0.692	100	98	98	22	-6	4.27E-6	6.27E-5	> 1.00E-4
SR	0.605	2.373	2.417	2.281	2.439	0.719	0.572	102	95	104	6	-6	3.57E-6	3.45E-5	> 1.00E-4
Non-Small Cell Lung Cancer															
A549/ATCC	0.313	2.194	2.106	2.225	2.149	0.475	0.119	95	102	98	9	-62	3.43E-6	1.32E-5	6.74E-5
EKVX	0.849	2.273	2.283	2.196	2.208	0.930	0.037	101	95	95	6	-96	3.21E-6	1.14E-5	3.54E-5
HOP-62	0.895	2.392	2.340	2.233	2.395	0.408	0.226	97	89	100	-54	-75	2.11E-6	4.45E-6	9.36E-6
HOP-92	1.140	1.646	1.671	1.510	1.609	1.145	0.331	105	73	93	1	-71	2.92E-6	1.03E-5	5.11E-5
NCI-H226	0.899	1.828	1.801	1.768	1.748	1.335	0.237	97	94	91	47	-74	8.52E-6	2.45E-5	6.36E-5
NCI-H23	0.639	2.105	2.011	2.018	1.989	0.228	0.125	94	94	92	-64	-80	1.86E-6	3.88E-6	8.10E-6
NCI-H322M	0.785	2.247	2.201	2.226	2.113	1.122	0.096	97	99	91	23	-88	4.00E-6	1.61E-5	4.56E-5
NCI-H460	0.302	2.716	2.970	2.808	2.704	0.252	0.214	111	104	99	-17	-29	2.67E-6	7.18E-6	> 1.00E-4
NCI-H522	1.442	3.298	3.245	3.261	3.260	0.661	0.249	97	98	98	-54	-83	2.07E-6	4.40E-6	9.39E-6
Colon Cancer															
COLO 205	0.714	2.567	2.455	2.563	2.531	0.201	0.150	94	100	98	-72	-79	1.92E-6	3.78E-6	7.44E-6
HCC-2998	0.978	3.223	3.199	3.166	3.141	0.104	0.051	99	97	96	-89	-95	1.78E-6	3.30E-6	6.14E-6
HCT-116	0.341	2.777	2.690	2.636	2.614	0.132	0.156	96	94	93	-61	-54	1.91E-6	4.01E-6	8.44E-6
HCT-15	0.333	2.599	2.523	2.431	2.408	0.061	0.017	97	93	92	-82	-95	1.74E-6	3.38E-6	6.56E-6
HT29	0.260	2.058	2.097	2.175	1.966	0.299	0.058	102	106	95	2	-78	3.05E-6	1.06E-5	4.48E-5
KM12	0.590	2.367	2.457	2.467	2.343	0.208	0.077	105	106	99	-65	-87	1.98E-6	4.01E-6	8.12E-6
SW-620	0.342	2.162	2.280	2.138	2.239	0.297	0.203	106	99	104	-13	-41	2.89E-6	7.71E-6	> 1.00E-4
CNS Cancer															
SF-268	0.855	2.356	2.274	2.236	2.113	0.999	0.278	95	92	84	10	-67	2.85E-6	1.33E-5	5.93E-5
SF-295	0.900	2.820	2.646	2.607	2.764	0.758	0.044	91	89	97	-16	-95	2.61E-6	7.25E-6	2.70E-5
SF-539	0.649	2.151	2.183	2.224	2.296	0.725	0.199	102	105	110	5	-69	3.72E-6	1.17E-5	5.49E-5
SNB-19	0.664	2.706	2.493	2.631	2.547	1.452	0.395	90	96	92	39	-41	6.12E-6	3.07E-5	> 1.00E-4
SNB-75	0.911	1.391	1.320	1.307	1.300	0.735	0.238	85	83	81	-19	-74	2.04E-6	6.42E-6	3.65E-5
U251	0.243	1.325	1.341	1.327	1.338	0.124	0.050	101	100	101	-49	-80	2.19E-6	4.72E-6	1.08E-5
Melanoma															
LOX IMVI	0.506	2.768	2.690	2.639	2.568	0.018	0.044	97	94	91	-97	-91	1.66E-6	3.06E-6	5.65E-6
MALME-3M	0.576	1.117	1.118	1.097	1.061	0.568	0.027	100	96	90	-1	-95	2.72E-6	9.63E-6	3.29E-5
M14	0.541	2.357	2.194	2.106	2.114	0.700	0.101	91	86	87	9	-81	2.95E-6	1.25E-5	4.49E-5
MDA-MB-435	0.630	2.185	2.200	2.096	2.109	0.485	0.093	101	94	95	-23	-85	2.41E-6	6.38E-6	2.71E-5
SK-MEL-2	1.280	2.527	2.460	2.333	2.375	1.854	0.101	95	84	88	46	-92	8.01E-6	2.15E-5	4.96E-5
SK-MEL-28	0.641	2.158	2.087	2.179	2.254	1.090	0.147	95	101	106	30	-77	5.42E-6	1.89E-5	5.57E-5
SK-MEL-5	0.933	3.143	2.767	2.921	2.972	2.177	0.043	83	90	92	56	-95	1.10E-5	2.35E-5	5.02E-5
UACC-257	1.011	2.625	2.568	2.487	2.597	1.819	0.149	96	91	98	50	-85	1.00E-5	2.34E-5	5.49E-5
UACC-62	0.846	3.066	3.027	2.925	2.941	2.103	0.296	98	94	94	57	-65	1.13E-5	2.92E-5	7.53E-5
Ovarian Cancer															
IGROV1	0.511	2.289	2.230	2.315	2.106	0.862	0.168	97	101	90	20	-67	3.69E-6	1.69E-5	6.34E-5
OVCAR-3	0.495	1.545	1.558	1.335	1.324	0.161	0.036	101	80	79	-68	-93	1.58E-6	3.46E-6	7.59E-6
OVCAR-4	0.875	2.002	1.978	1.873	1.910	1.075	0.134	98	89	92	18	-85	3.67E-6	1.49E-5	4.58E-5
OVCAR-5	0.691	2.423	2.456	2.573	2.648	1.267	0.137	102	109	113	33	-80	6.16E-6	1.96E-5	5.42E-5
OVCAR-8	0.419	2.300	2.267	2.239	2.220	0.690	0.147	98	97	96	14	-65	3.65E-6	1.52E-5	6.47E-5
NCI/ADR-RES	0.527	1.941	1.956	1.894	1.878	0.480	0.225	101	97	96	-9	-57	2.73E-6	8.21E-6	7.06E-5
SK-OV-3	0.803	1.948	1.912	1.825	1.821	1.014	0.188	97	89	89	18	-77	3.56E-6	1.56E-5	5.24E-5
Renal Cancer															
786-0	0.761	2.921	2.909	2.881	2.973	0.341	0.183	99	98	102	-55	-76	2.15E-6	4.46E-6	9.26E-6
A498	1.228	2.222	2.186	2.162	2.225	1.499	0.112	96	94	100	27	-91	4.88E-6	1.70E-5	4.51E-5
ACHN	0.295	1.427	1.447	1.370	1.422	0.384	0.051	102	95	100	8	-83	3.47E-6	1.22E-5	4.34E-5
CAKI-1	0.802	2.367	2.186	2.141	2.145	0.925	0.088	88	86	86	8	-89	2.88E-6	1.21E-5	3.96E-5
RXF 393	0.864	1.692	1.664	1.631	1.636	0.953	0.255	97	93	93	11	-70	3.34E-6	1.35E-5	5.59E-5
SN12C	0.613	2.692	2.805	2.578	2.648	0.354	0.365	105	95	98	-42	-40	2.20E-6	4.99E-6	> 1.00E-4
TK-10	1.357	2.314	2.219	2.156	2.392	2.170	0.129	90	83	108	85	-91	1.58E-5	3.05E-5	5.87E-5
UO-31	0.493	1.894	1.746	1.594	1.587	0.585	0.111	89	79	78	7	-77	2.47E-6	1.20E-5	4.71E-5
Prostate Cancer															
PC-3	0.613	2.021	2.055	1.799	1.779	0.731	0.219	102	84	83	8	-64	2.76E-6	1.30E-5	6.36E-5
DU-145	0.406	1.698	1.770	1.670	1.535	0.314	0.030	106	98	87	-23	-93	2.19E-6	6.22E-6	2.46E-5
Breast Cancer															
MCF7	0.509	2.471	2.359	2.384	2.359	0.528	0.191	94	96	94	1	-63	2.98E-6	1.04E-5	6.34E-5
MDA-MB-231/ATCC	0.522	1.318	1.442	1.305	1.394	0.189	0.061	116	98	110	-64	-88	2.21E-6	4.28E-6	8.33E-6
HS 578T	0.945	2.089	2.024	2.191	1.944	1.051	0.758	94	109	87	9	-20	3.01E-6	2.08E-5	> 1.00E-4
BT-549	1.295	2.600	2.577	2.504	2.342	1.567	0.226	98	93	80	21	-83	3.23E-6	1.59E-5	4.84E-5
T-47D	0.533	1.122	1.024	1.058	1.002	0.469	0.184	83	89	80	-12	-66	2.10E-6	7.38E-6	5.11E-5
MDA-MB-468	0.726	1.488	1.483	1.463	1.444	0.765	0.125	99	97	94	5	-83	3.14E-6	1.14E-5	4.23E-5

Figure S88. Values of log molar concentration of response parameters ($\log_{10}$ GI_{50}, $\log_{10}$ TGI & $\log_{10}$ LC_{50}) for compound **10b**

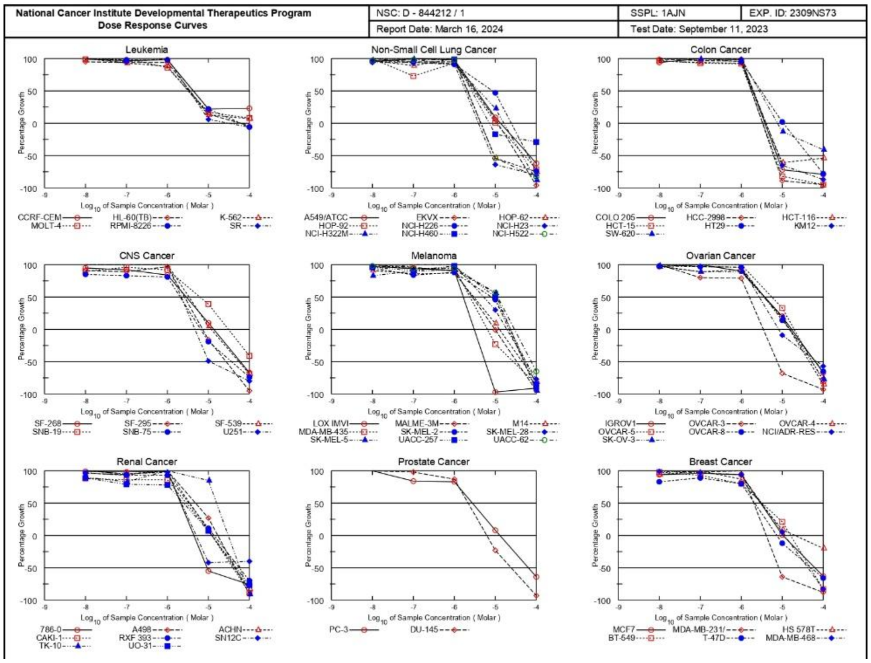

Figure S89. Dose-response curves (% growth versus sample concentration) for all cell lines with different subpanel obtained from the NCI's in vitro disease-oriented human cancer cells line for compound **10b** on nine types of cancer

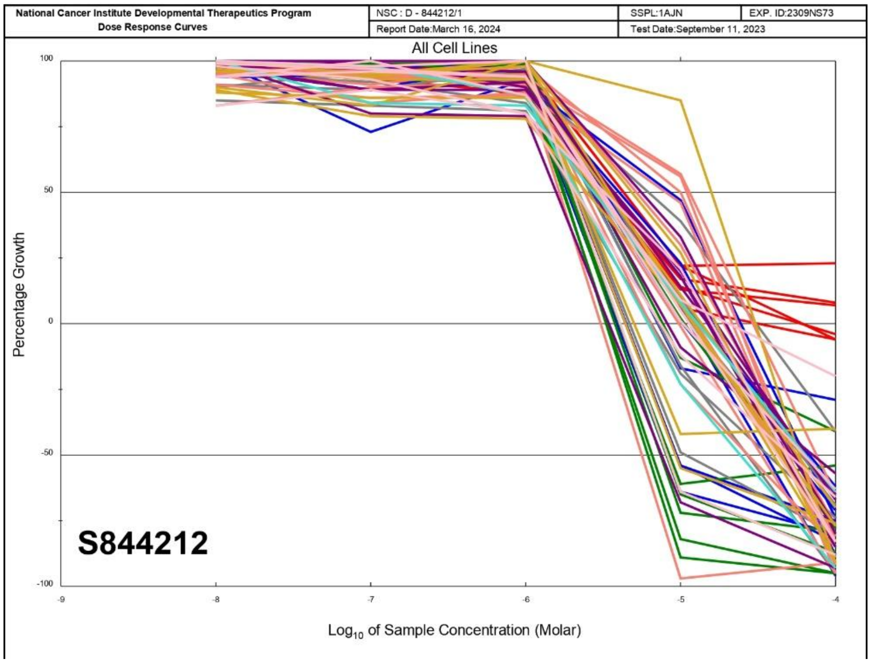

Figure S90. Dose-response curves for all cell lines in the NCI60 panel exposed compound **10b** with tissue originated colors and shapes.

Developmental Therapeutics Program One Dose Mean Graph	NSC: D-841970 / 1	Conc: 1.00E-5 Molar	Test Date: Feb 06, 2023
	Experiment ID: 2302OS94		Report Date: Mar 13, 2023

Panel/Cell Line	Growth Percent
**Leukemia**	
CCRF-CEM	8.50
HL-60(TB)	-16.74
K-562	5.46
MOLT-4	0.49
RPMI-8226	8.27
**Non-Small Cell Lung Cancer**	
A549/ATCC	7.90
EKVX	-45.42
HOP-62	-18.28
HOP-92	11.11
NCI-H226	-38.63
NCI-H23	-76.03
NCI-H322M	2.56
NCI-H460	-3.70
NCI-H522	-66.18
**Colon Cancer**	
COLO 205	-94.20
HCC-2998	-91.67
HCT-116	-24.71
HCT-15	-74.46
HT29	-47.26
KM12	-69.27
SW-620	-78.42
**CNS Cancer**	
SF-268	44.54
SF-295	-61.72
SF-539	-65.05
SNB-19	13.42
SNB-75	53.39
U251	-45.14
**Melanoma**	
LOX IMVI	-99.60
MALME-3M	-56.09
M14	-28.35
MDA-MB-435	-40.09
SK-MEL-2	71.75
SK-MEL-28	7.34
SK-MEL-5	-33.55
UACC-257	49.25
UACC-62	36.72
**Ovarian Cancer**	
IGROV1	2.62
OVCAR-3	-50.73
OVCAR-4	30.93
OVCAR-5	2.98
OVCAR-8	1.21
NCI/ADR-RES	-31.44
SK-OV-3	66.96
**Renal Cancer**	
786-0	-6.39
A498	-7.81
ACHN	0.76
CAKI-1	-8.29
RXF 393	-33.17
SN12C	-61.51
TK-10	55.82
UO-31	-27.41
**Prostate Cancer**	
PC-3	2.09
DU-145	-47.72
**Breast Cancer**	
MCF7	-29.87
MDA-MB-231/ATCC	-43.31
HS 578T	-1.03
BT-549	-47.85
T-47D	26.16
MDA-MB-468	-38.77
Mean	-18.64
Delta	80.96
Range	171.35

Mean Growth Percent - Growth Percent

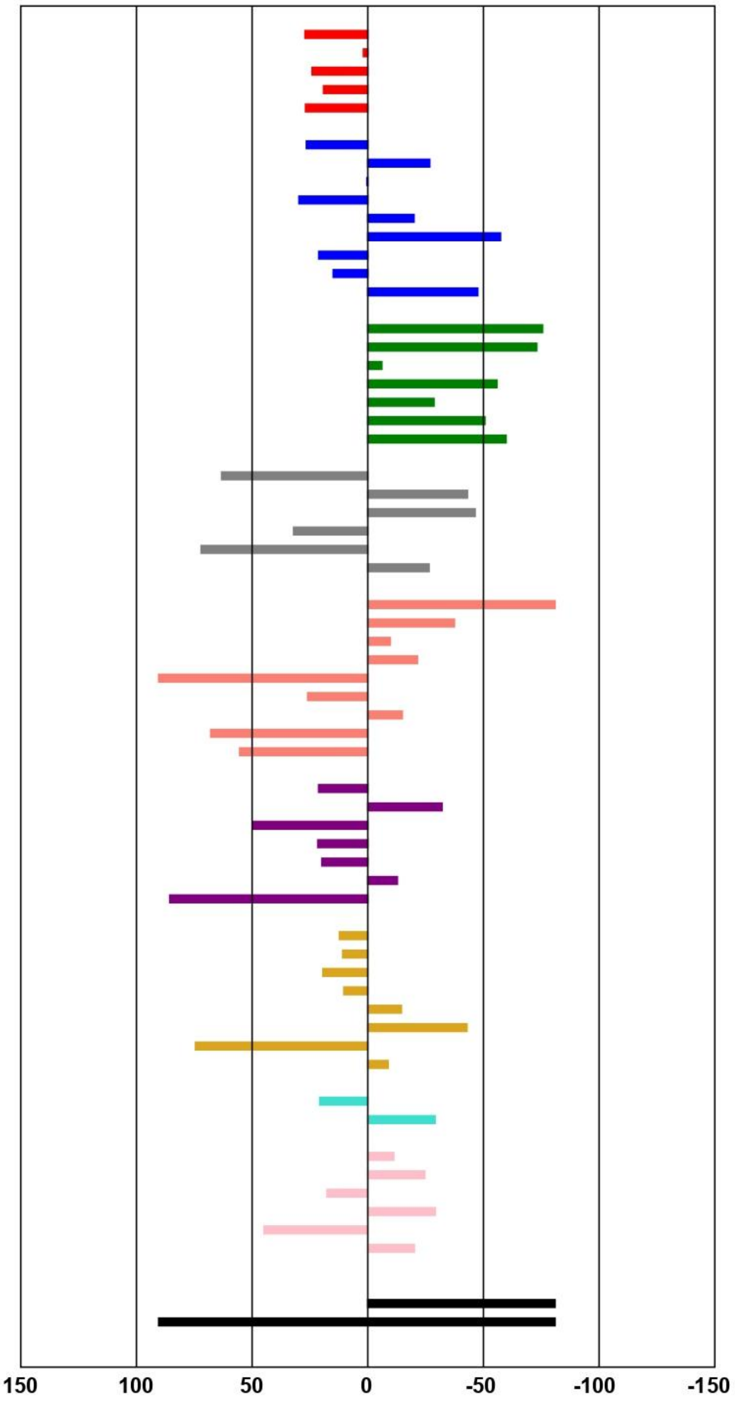

Figure S91. One dose mean graph for compound **10c** at 10 μM

National Cancer Institute Developmental Therapeutics Program In-Vitro Testing Results

NSC : D - 841970 / 1	Experiment ID : 2307NS43	Test Type : 08	Units : Molar
Report Date : March 16, 2024	Test Date : July 10, 2023	QNS :	MC :
COMI : H1-10c	Stain Reagent : SRB Dual-Pass Related	SSPL : 1AJN	

			Log10 Concentration												
	Time		Mean Optical Densities					Percent Growth							
Panel/Cell Line	Zero	Ctrl	-8.0	-7.0	-6.0	-5.0	-4.0	-8.0	-7.0	-6.0	-5.0	-4.0	GI50	TGI	LC50
**Leukemia**															
CCRF-CEM	0.418	2.318	1.956	1.971	1.840	0.804	0.696	81	82	75	20	15	2.85E-6	> 1.00E-4	> 1.00E-4
HL-60(TB)	0.393	1.363	1.268	1.333	1.409	0.359	0.321	90	97	105	-9	-18	3.04E-6	8.39E-6	> 1.00E-4
K-562	0.218	1.890	1.851	1.846	1.697	0.226	0.176	98	97	88	0	-19	2.74E-6	1.06E-5	> 1.00E-4
MOLT-4	0.497	1.835	1.790	1.654	1.549	0.416	0.308	97	86	79	-16	-38	2.00E-6	6.72E-6	> 1.00E-4
RPMI-8226	0.736	2.729	2.839	2.867	2.786	1.428	0.779	106	107	103	35	2	5.96E-6	> 1.00E-4	> 1.00E-4
SR	0.438	2.309	2.192	2.166	2.020	0.464	0.402	94	92	85	1	-8	2.60E-6	1.40E-5	> 1.00E-4
**Non-Small Cell Lung Cancer**															
A549/ATCC	0.509	2.503	2.410	2.329	2.373	0.952	0.205	95	91	93	22	-60	4.07E-6	1.86E-5	7.59E-5
EKVX	0.807	1.898	1.712	1.771	1.774	0.686	0.319	83	88	89	-15	-61	2.36E-6	7.16E-6	5.87E-5
HOP-62	0.734	2.411	2.751	2.819	2.738	0.598	0.510	120	124	119	-19	-31	3.19E-6	7.34E-6	> 1.00E-4
HOP-92	1.384	1.846	1.758	1.731	1.825	1.398	0.369	81	75	95	3	-73	3.10E-6	1.10E-5	4.94E-5
NCI-H226	0.974	1.556	1.476	1.420	1.518	1.185	0.254	86	77	93	36	-74	5.73E-6	2.13E-5	6.06E-5
NCI-H23	0.837	1.968	1.847	1.825	1.839	0.519	0.118	89	87	89	-38	-86	2.02E-6	5.01E-6	1.78E-5
NCI-H322M	0.529	1.932	1.850	1.767	1.859	1.086	0.028	94	88	95	40	-95	6.49E-6	1.97E-5	4.65E-5
NCI-H460	0.284	2.861	2.877	2.726	2.763	0.332	0.247	101	95	96	2	-13	3.09E-6	1.33E-5	> 1.00E-4
NCI-H522	1.097	2.638	2.513	2.482	2.498	0.384	0.242	92	90	91	-65	-78	1.83E-6	3.83E-6	8.01E-6
**Colon Cancer**															
COLO 205	0.517	2.510	2.354	2.420	2.494	0.459	0.396	92	96	99	-11	-24	2.79E-6	7.91E-6	> 1.00E-4
HCC-2998	0.628	1.671	1.616	1.534	1.648	0.308	0.021	95	87	98	-51	-97	2.09E-6	4.54E-6	9.85E-6
HCT-116	0.186	1.715	1.614	1.601	1.670	0.129	0.131	93	93	97	-31	-30	2.33E-6	5.73E-6	> 1.00E-4
HCT-15	0.301	1.809	1.699	1.634	1.679	0.509	0.010	93	88	91	14	-97	3.41E-6	1.33E-5	3.77E-5
HT29	0.324	2.015	1.975	1.894	1.956	0.375	0.081	98	93	96	3	-75	3.14E-6	1.09E-5	4.77E-5
KM12	0.481	2.367	2.349	2.228	2.326	0.488	0.217	99	93	98	0	-55	3.09E-6	1.02E-5	8.13E-5
SW-620	0.294	2.397	2.326	2.278	2.302	1.697	0.990	97	94	95	67	33	3.14E-5	> 1.00E-4	> 1.00E-4
**CNS Cancer**															
SF-268	1.122	2.933	2.796	2.744	2.711	1.791	0.534	92	90	88	37	-52	5.53E-6	2.59E-5	9.39E-5
SF-295	0.657	2.184	2.036	1.962	2.047	1.216	0.165	90	85	91	37	-75	5.67E-6	2.13E-5	5.98E-5
SF-539	0.796	2.386	2.375	2.258	2.376	1.496	0.050	99	92	99	44	-94	7.79E-6	2.09E-5	4.81E-5
SNB-75	1.106	1.962	1.700	1.619	1.782	1.319	0.139	69	60	79	25	-87	3.43E-6	1.66E-5	4.64E-5
U251	0.366	1.974	1.982	1.954	1.875	0.473	0.085	100	99	94	7	-77	3.18E-6	1.20E-5	4.76E-5
**Melanoma**															
LOX IMVI	0.434	2.322	2.145	2.131	2.263	0.124	0.039	91	90	97	-71	-91	1.90E-6	3.76E-6	7.46E-6
MALME-3M	0.419	1.061	1.052	1.043	1.008	0.470	0.094	98	97	92	8	-78	3.14E-6	1.24E-5	4.75E-5
M14	0.546	2.390	2.452	2.299	2.335	1.194	0.265	103	95	97	35	-52	5.75E-6	2.54E-5	9.60E-5
MDA-MB-435	0.524	2.227	2.061	1.967	2.086	0.648	0.085	90	85	92	7	-84	3.12E-6	1.20E-5	4.25E-5
SK-MEL-2	1.307	3.065	3.021	3.003	3.016	2.375	0.110	97	96	97	61	-92	1.18E-5	2.50E-5	5.33E-5
SK-MEL-28	0.650	1.896	1.839	1.854	1.846	1.026	0.030	95	97	96	30	-95	5.00E-6	1.74E-5	4.35E-5
SK-MEL-5	0.961	2.891	2.850	2.888	2.888	2.300	0.018	98	100	100	69	-98	1.31E-5	2.60E-5	5.16E-5
UACC-257	1.082	2.621	2.477	2.419	2.530	1.840	0.254	91	87	94	49	-77	9.63E-6	2.46E-5	6.15E-5
UACC-62	0.762	2.321	2.131	2.226	2.160	1.365	0.034	88	94	90	39	-96	6.00E-6	1.94E-5	4.57E-5
**Ovarian Cancer**															
IGROV1	0.228	1.321	1.451	1.576	1.639	0.531	0.079	112	123	129	28	-65	6.03E-6	1.99E-5	6.84E-5
OVCAR-3	0.589	1.989	1.935	1.881	1.930	0.640	0.163	96	92	96	4	-72	3.14E-6	1.12E-5	5.08E-5
OVCAR-4	0.909	2.063	1.984	1.953	1.946	1.330	0.202	93	90	90	36	-78	5.57E-6	2.08E-5	5.71E-5
OVCAR-5	0.511	1.559	1.474	1.458	1.526	0.874	0.012	92	90	97	35	-98	5.66E-6	1.83E-5	4.36E-5
OVCAR-8	0.699	2.819	2.721	2.741	2.771	1.258	0.157	95	96	98	26	-78	4.67E-6	1.79E-5	5.43E-5
NCI/ADR-RES	0.445	1.295	1.261	1.250	1.276	0.702	0.068	96	95	98	30	-85	5.10E-6	1.83E-5	4.98E-5
SK-OV-3	1.032	2.497	2.365	2.342	2.327	1.976	0.377	91	89	88	64	-63	1.30E-5	3.19E-5	7.85E-5
**Renal Cancer**															
786-0	0.909	3.080	2.949	2.950	2.984	1.438	0.374	94	94	96	24	-59	4.37E-6	1.96E-5	7.83E-5
A498	1.400	2.604	2.617	2.582	2.497	2.296	0.511	101	98	91	74	-64	1.50E-5	3.46E-5	7.98E-5
ACHN	0.301	1.377	1.381	1.361	1.364	0.419	0.007	100	99	99	11	-98	3.59E-6	1.26E-5	3.64E-5
CAKI-1	0.799	2.630	2.466	2.392	2.416	1.071	0.070	91	87	88	15	-91	3.32E-6	1.38E-5	4.09E-5
RXF 393	0.921	1.499	1.422	1.412	1.444	1.090	0.183	87	85	90	29	-80	4.57E-6	1.85E-5	5.30E-5
SN12C	0.640	2.132	2.006	2.044	2.012	0.543	0.074	92	94	92	-15	-89	2.46E-6	7.21E-6	2.98E-5
UO-31	0.571	1.921	1.704	1.769	1.812	1.160	0.010	84	89	92	44	-98	7.38E-6	2.03E-5	4.57E-5
**Prostate Cancer**															
PC-3	0.621	2.165	2.203	2.083	2.106	0.705	0.215	102	95	96	5	-65	3.23E-6	1.19E-5	6.07E-5
DU-145	0.467	2.154	2.116	1.931	1.813	0.652	0.211	98	87	80	11	-55	2.71E-6	1.47E-5	8.45E-5
**Breast Cancer**															
MCF7	0.336	1.678	1.568	1.585	1.560	0.354	0.058	92	93	91	1	-83	2.87E-6	1.04E-5	4.07E-5
MDA-MB-231/ATCC	0.524	1.150	1.127	1.100	1.138	0.321	0.088	96	92	98	-39	-83	2.25E-6	5.21E-6	1.79E-5
HS 578T	1.160	2.271	2.291	2.283	2.279	0.387	0.235	102	101	101	-67	-80	2.01E-6	4.00E-6	7.95E-6
BT-549	1.006	2.034	1.923	1.872	1.943	1.321	0.441	89	84	91	31	-56	4.78E-6	2.25E-5	8.48E-5
T-47D	0.718	2.098	2.019	1.983	1.820	1.089	0.450	94	92	80	27	-37	3.66E-6	2.62E-5	> 1.00E-4
MDA-MB-468	0.720	1.045	1.003	0.986	1.067	0.805	0.126	87	82	107	26	-83	5.04E-6	1.74E-5	5.02E-5

Figure S92. Values of log molar concentration of response parameters ($\log_{10}$ GI_{50}, $\log_{10}$ TGI & $\log_{10}$ LC_{50}) for compound **10c**

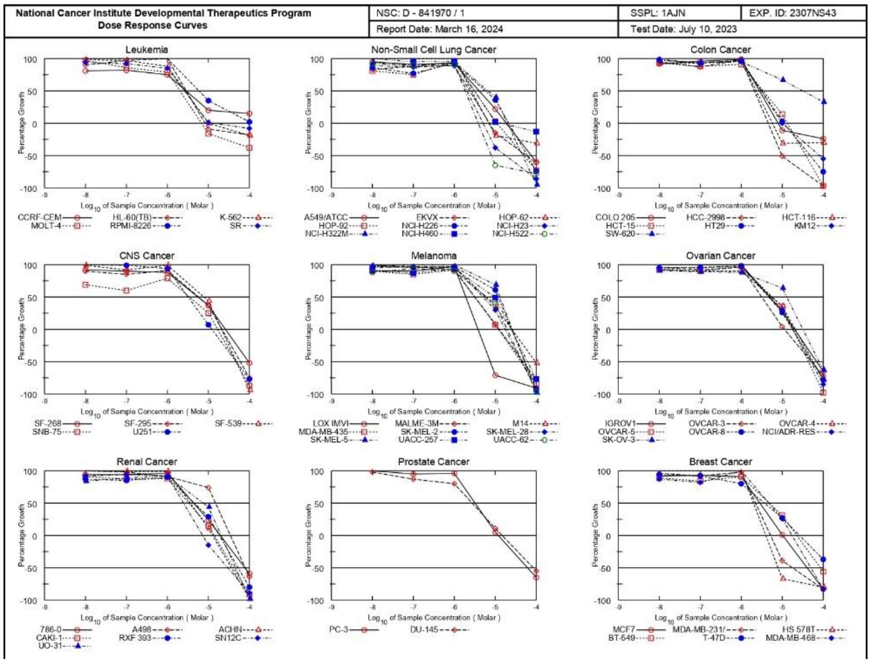

Figure S93. Dose-response curves (% growth versus sample concentration) for all cell lines with different subpanel obtained from the NCI's in vitro disease-oriented human cancer cells line for compound **10c** on nine types of cancer

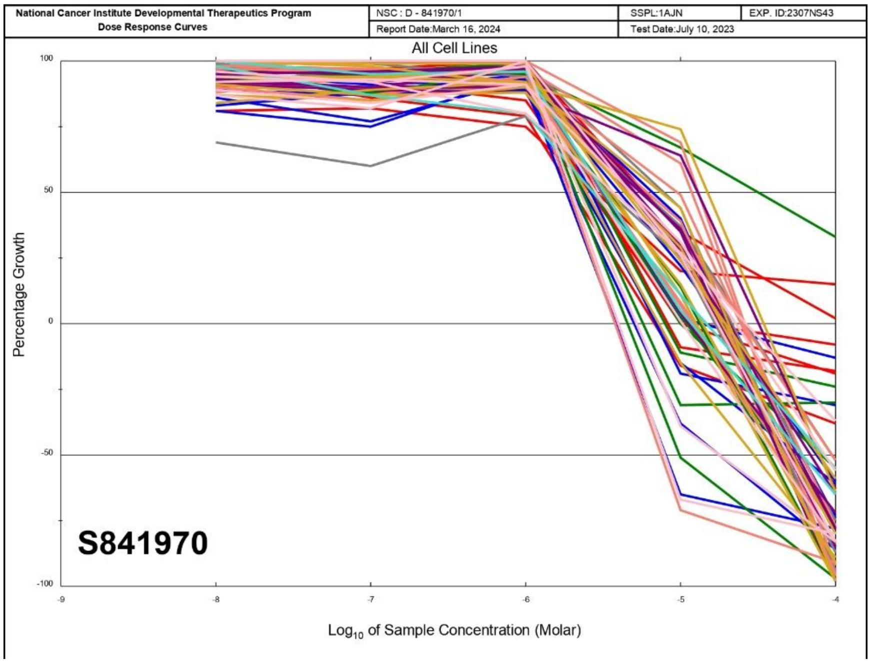

Figure S94. Dose-response curves for all cell lines in the NCI60 panel exposed compound **10c** with tissue originated colors and shapes.

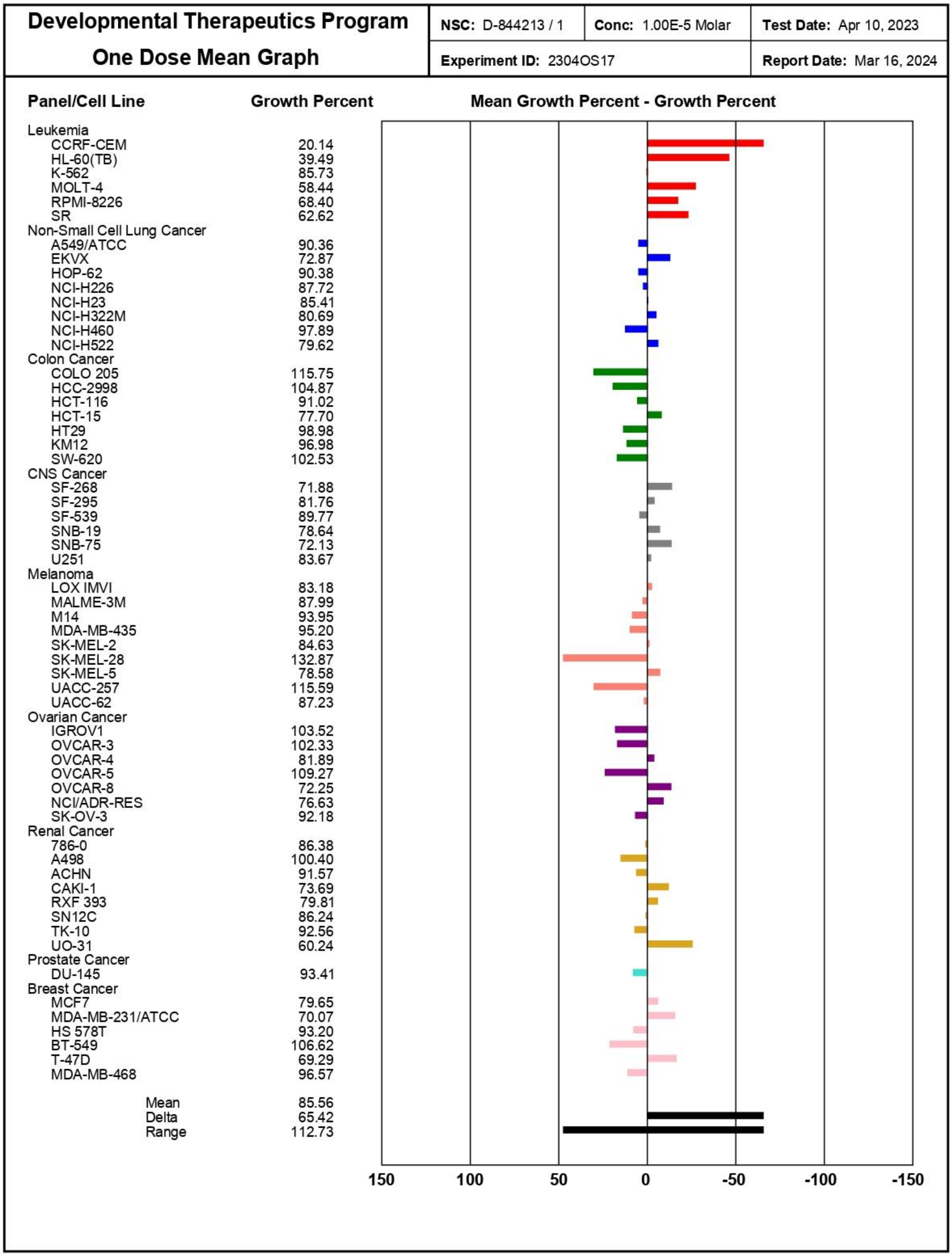

Developmental Therapeutics Program	NSC: D-844213 / 1	Conc: 1.00E-5 Molar	Test Date: Apr 10, 2023
One Dose Mean Graph	Experiment ID: 2304OS17		Report Date: Mar 16, 2024

Panel/Cell Line	Growth Percent
Leukemia	
CCRF-CEM	20.14
HL-60(TB)	39.49
K-562	85.73
MOLT-4	58.44
RPMI-8226	68.40
SR	62.62
Non-Small Cell Lung Cancer	
A549/ATCC	90.36
EKVX	72.87
HOP-62	90.38
NCI-H226	87.72
NCI-H23	85.41
NCI-H322M	80.69
NCI-H460	97.89
NCI-H522	79.62
Colon Cancer	
COLO 205	115.75
HCC-2998	104.87
HCT-116	91.02
HCT-15	77.70
HT29	98.98
KM12	96.98
SW-620	102.53
CNS Cancer	
SF-268	71.88
SF-295	81.76
SF-539	89.77
SNB-19	78.64
SNB-75	72.13
U251	83.67
Melanoma	
LOX IMVI	83.18
MALME-3M	87.99
M14	93.95
MDA-MB-435	95.20
SK-MEL-2	84.63
SK-MEL-28	132.87
SK-MEL-5	78.58
UACC-257	115.59
UACC-62	87.23
Ovarian Cancer	
IGROV1	103.52
OVCAR-3	102.33
OVCAR-4	81.89
OVCAR-5	109.27
OVCAR-8	72.25
NCI/ADR-RES	76.63
SK-OV-3	92.18
Renal Cancer	
786-0	86.38
A498	100.40
ACHN	91.57
CAKI-1	73.69
RXF 393	79.81
SN12C	86.24
TK-10	92.56
UO-31	60.24
Prostate Cancer	
DU-145	93.41
Breast Cancer	
MCF7	79.65
MDA-MB-231/ATCC	70.07
HS 578T	93.20
BT-549	106.62
T-47D	69.29
MDA-MB-468	96.57
Mean	85.56
Delta	65.42
Range	112.73

Figure S95. One dose mean graph for compound **10d** at 10 μM

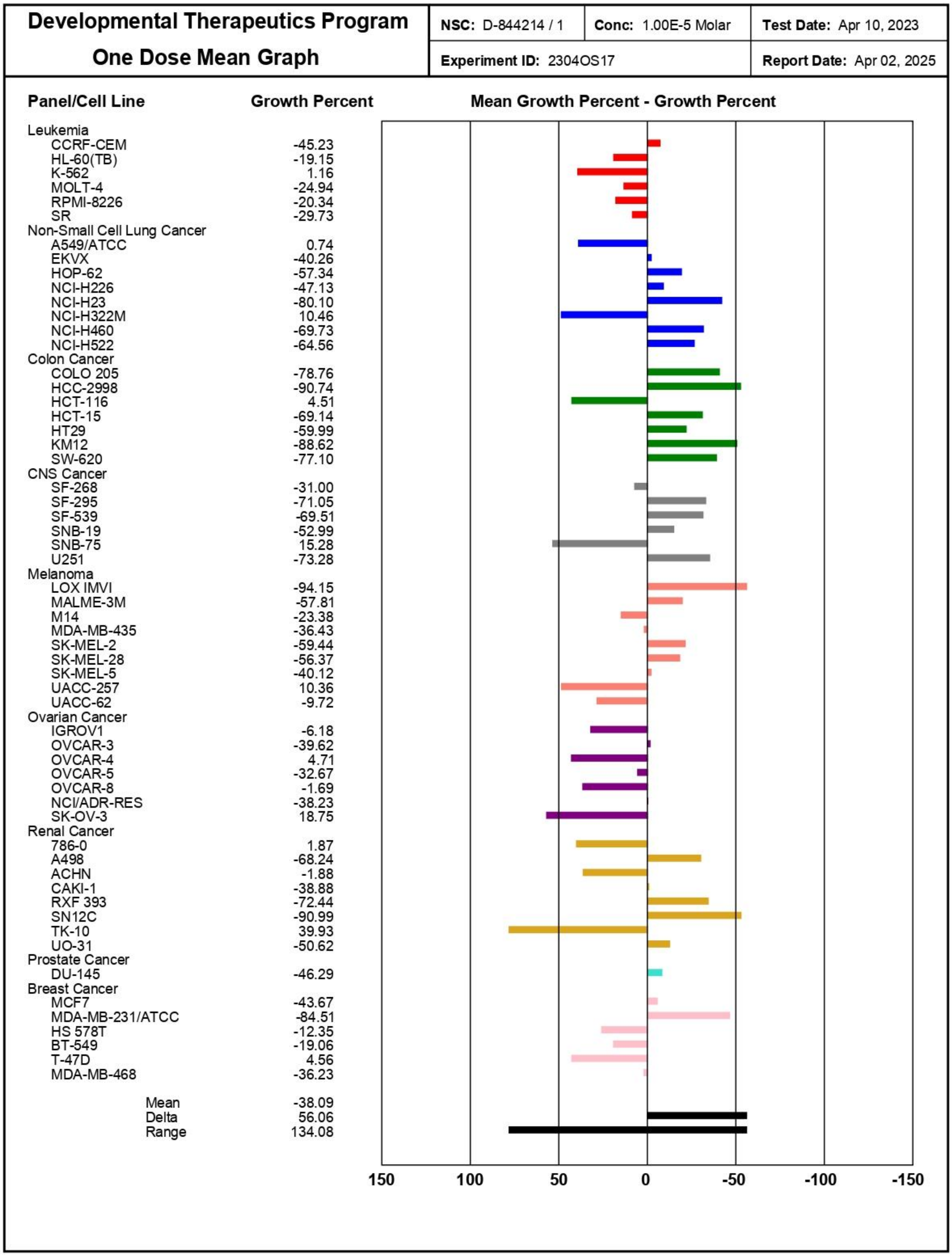

Figure S96. One dose mean graph for compound **10e** at 10 μM

National Cancer Institute Developmental Therapeutics Program
In-Vitro Testing Results

NSC : D - 844214 / 1	Experiment ID : 2309NS73	Test Type : 08	Units : Molar
Report Date : March 16, 2024	Test Date : September 11, 2023	QNS :	MC :
COMI : H1-10e	Stain Reagent : SRB Dual-Pass Related	SSPL : 1AJN	

			Log10 Concentration												
	Time		Mean Optical Densities					Percent Growth							
Panel/Cell Line	Zero	Ctrl	-8.0	-7.0	-6.0	-5.0	-4.0	-8.0	-7.0	-6.0	-5.0	-4.0	GI50	TGI	LC50
Leukemia															
CCRF-CEM	0.517	2.849	2.756	2.745	2.648	0.576	0.586	96	96	91	3	3	2.92E-6	> 1.00E-4	> 1.00E-4
HL-60(TB)	0.600	2.790	2.736	2.711	2.543	0.719	0.605	98	96	89	5	0	2.92E-6	> 1.00E-4	> 1.00E-4
K-562	0.212	2.210	2.213	2.206	1.987	0.284	0.337	100	100	89	4	6	2.85E-6	> 1.00E-4	> 1.00E-4
MOLT-4	0.451	2.361	2.421	2.252	1.942	0.506	0.477	103	94	78	3	1	2.36E-6	> 1.00E-4	> 1.00E-4
RPMI-8226	0.732	2.678	2.549	2.508	2.429	0.692	0.686	93	91	87	-5	-6	2.52E-6	8.73E-6	> 1.00E-4
SR	0.605	2.373	2.399	2.346	2.189	0.554	0.555	101	98	90	-9	-8	2.53E-6	8.19E-6	> 1.00E-4
Non-Small Cell Lung Cancer															
A549/ATCC	0.313	2.194	2.089	2.084	1.848	0.449	0.061	94	94	82	7	-81	2.66E-6	1.21E-5	4.49E-5
EKVX	0.849	2.273	2.206	2.273	2.145	0.830	0.035	95	100	91	-2	-96	2.75E-6	9.45E-6	3.23E-5
HOP-62	0.895	2.392	2.369	2.264	2.118	0.430	0.429	98	91	82	-52	-52	1.73E-6	4.08E-6	9.66E-6
HOP-92	1.140	1.646	1.510	1.618	1.625	1.094	0.541	73	95	96	-4	-53	2.87E-6	9.10E-6	8.86E-5
NCI-H226	0.899	1.828	1.779	1.778	1.719	1.253	0.314	95	95	88	38	-65	5.80E-6	2.34E-5	7.14E-5
NCI-H23	0.639	2.105	2.034	2.042	1.960	0.283	0.128	95	96	90	-56	-80	1.88E-6	4.15E-6	9.14E-6
NCI-H322M	0.785	2.247	2.243	2.335	2.012	1.041	0.057	100	106	84	18	-93	3.24E-6	1.44E-5	4.09E-5
NCI-H460	0.302	2.716	2.565	2.592	2.441	0.092	0.124	94	95	89	-70	-59	1.75E-6	3.63E-6	7.52E-6
NCI-H522	1.442	3.298	3.163	3.217	3.144	0.404	0.274	93	96	92	-72	-81	1.80E-6	3.63E-6	7.34E-6
Colon Cancer															
COLO 205	0.714	2.567	2.442	2.447	2.330	0.121	0.061	93	94	87	-83	-91	1.65E-6	3.25E-6	6.40E-6
HCC-2998	0.978	3.223	3.132	3.225	3.222	0.129	0.014	96	100	100	-87	-99	1.85E-6	3.43E-6	6.35E-6
HCT-116	0.341	2.777	2.759	2.746	2.453	0.158	0.149	99	99	87	-54	-56	1.83E-6	4.14E-6	9.39E-6
HCT-15	0.333	2.599	2.492	2.540	2.422	0.268	0.011	95	97	92	-20	-97	2.39E-6	6.69E-6	2.48E-5
HT29	0.260	2.058	2.006	1.966	1.794	0.135	0.006	97	95	85	-48	-98	1.84E-6	4.35E-6	1.08E-5
KM12	0.590	2.367	2.355	2.420	2.192	0.281	0.032	99	103	90	-52	-95	1.91E-6	4.29E-6	9.62E-6
SW-620	0.342	2.162	1.809	1.954	1.730	0.095	0.106	81	89	76	-72	-69	1.50E-6	3.26E-6	7.07E-6
CNS Cancer															
SF-268	0.855	2.356	2.301	2.329	2.206	0.942	0.229	96	98	90	6	-73	2.98E-6	1.18E-5	5.08E-5
SF-295	0.900	2.820	2.578	2.669	2.686	0.733	0.027	87	92	93	-19	-97	2.43E-6	6.82E-6	2.52E-5
SF-539	0.649	2.151	2.113	2.154	1.909	0.530	0.061	98	100	84	-18	-91	2.15E-6	6.62E-6	2.74E-5
SNB-19	0.664	2.706	2.630	2.592	2.578	1.160	0.077	96	94	94	24	-88	4.26E-6	1.64E-5	4.56E-5
SNB-75	0.911	1.391	1.299	1.370	1.334	0.684	0.398	81	96	88	-25	-56	2.17E-6	6.02E-6	6.29E-5
U251	0.243	1.325	1.297	1.277	1.249	0.079	0.032	97	96	93	-67	-87	1.85E-6	3.80E-6	7.78E-6
Melanoma															
LOX IMVI	0.506	2.768	2.690	2.716	2.644	0.008	0.021	97	98	95	-99	-96	1.70E-6	3.09E-6	5.61E-6
MALME-3M	0.576	1.117	1.059	1.102	0.997	0.307	0.005	89	97	78	-47	-99	1.67E-6	4.21E-6	1.15E-5
M14	0.541	2.357	2.146	2.123	2.005	0.551	0.116	88	87	81	1	-79	2.41E-6	1.02E-5	4.35E-5
MDA-MB-435	0.630	2.185	2.110	2.083	2.026	0.460	0.038	95	93	90	-27	-94	2.19E-6	5.87E-6	2.20E-5
SK-MEL-2	1.280	2.527	2.420	2.473	2.339	1.615	0.020	91	96	85	27	-98	3.99E-6	1.64E-5	4.10E-5
SK-MEL-28	0.641	2.158	2.082	2.215	1.916	0.736	0.081	95	104	84	6	-87	2.74E-6	1.17E-5	3.99E-5
SK-MEL-5	0.933	3.143	2.953	3.030	2.900	1.618	0.009	91	95	89	31	-99	4.70E-6	1.73E-5	4.19E-5
UACC-257	1.011	2.625	2.444	2.540	2.532	1.470	0.224	89	95	94	28	-78	4.70E-6	1.85E-5	5.47E-5
UACC-62	0.846	3.066	3.087	3.019	2.872	1.879	0.111	101	98	91	47	-87	8.37E-6	2.23E-5	5.29E-5
Ovarian Cancer															
IGROV1	0.511	2.289	2.274	2.242	1.978	0.693	0.137	99	97	82	10	-73	2.81E-6	1.33E-5	5.26E-5
OVCAR-3	0.495	1.545	1.585	1.695	1.450	0.207	0.037	104	114	91	-58	-93	1.88E-6	4.07E-6	8.81E-6
OVCAR-4	0.875	2.002	1.955	1.973	1.900	0.914	0.248	96	97	91	3	-72	2.94E-6	1.11E-5	5.14E-5
OVCAR-5	0.691	2.423	2.387	2.421	2.186	0.886	0.063	98	100	86	11	-91	3.05E-6	1.29E-5	3.97E-5
OVCAR-8	0.419	2.300	2.188	2.271	2.161	0.518	0.131	94	98	93	5	-69	3.08E-6	1.18E-5	5.57E-5
NCI/ADR-RES	0.527	1.941	1.970	1.956	1.912	0.542	0.263	102	101	98	1	-50	3.12E-6	1.05E-5	9.92E-5
SK-OV-3	0.803	1.948	1.822	1.756	1.664	0.845	0.219	89	83	75	4	-73	2.25E-6	1.12E-5	5.04E-5
Renal Cancer															
786-0	0.761	2.921	2.899	2.911	2.774	0.265	0.310	99	100	93	-65	-59	1.87E-6	3.88E-6	8.02E-6
A498	1.228	2.222	2.141	2.123	2.146	1.201	0.215	92	90	92	-2	-82	2.81E-6	9.47E-6	3.94E-5
ACHN	0.295	1.427	1.386	1.458	1.351	0.374	0.016	96	103	93	7	-95	3.17E-6	1.17E-5	3.64E-5
CAKI-1	0.802	2.367	2.192	2.205	2.113	0.838	0.078	89	90	84	2	-90	2.60E-6	1.06E-5	3.67E-5
RXF 393	0.864	1.692	1.643	1.654	1.654	0.781	0.414	94	95	95	-10	-52	2.71E-6	8.09E-6	8.90E-5
SN12C	0.613	2.692	2.651	2.671	2.522	0.285	0.159	98	99	92	-54	-74	1.94E-6	4.28E-6	9.46E-6
TK-10	1.357	2.314	2.182	2.216	2.194	1.614	0.069	86	90	87	27	-95	4.14E-6	1.66E-5	4.28E-5
UO-31	0.493	1.894	1.714	1.733	1.690	0.373	0.064	87	88	85	-24	-87	2.10E-6	6.00E-6	2.57E-5
Prostate Cancer															
PC-3	0.613	2.021	1.861	1.860	1.829	0.666	0.389	89	89	86	4	-37	2.76E-6	1.24E-5	> 1.00E-4
DU-145	0.406	1.698	1.704	1.677	1.500	0.317	0.019	100	98	85	-22	-95	2.11E-6	6.22E-6	2.40E-5
Breast Cancer															
MCF7	0.509	2.471	2.311	2.320	2.187	0.538	0.148	92	92	85	1	-71	2.64E-6	1.05E-5	5.13E-5
MDA-MB-231/ATCC	0.522	1.318	1.333	1.350	1.323	0.113	0.103	102	104	101	-78	-80	1.92E-6	3.65E-6	6.94E-6
HS 578T	0.945	2.089	1.797	1.932	1.747	0.834	0.816	74	86	70	-12	-14	1.76E-6	7.18E-6	> 1.00E-4
BT-549	1.295	2.600	2.460	2.529	2.622	1.449	0.818	89	95	102	12	-37	3.76E-6	1.75E-5	> 1.00E-4
T-47D	0.533	1.122	1.020	1.092	1.030	0.522	0.396	83	95	84	-2	-26	2.50E-6	9.46E-6	> 1.00E-4
MDA-MB-468	0.726	1.488	1.458	1.499	1.446	0.686	0.034	96	102	95	-6	-95	2.79E-6	8.81E-6	3.13E-5

Figure S97. Values of log molar concentration of response parameters ($\log_{10}$ GI_{50}, $\log_{10}$ TGI & $\log_{10}$ LC_{50}) for compound **10e**

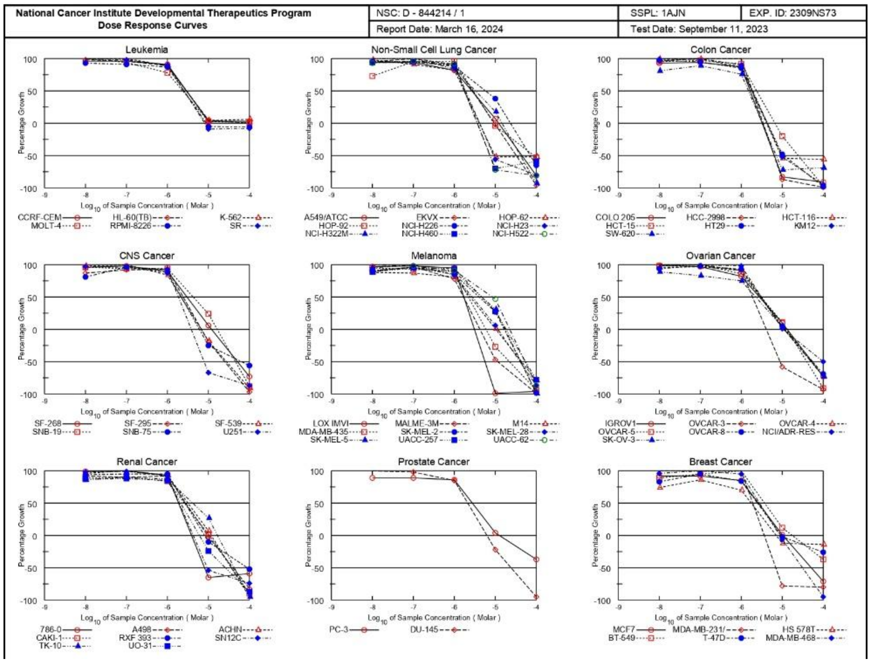

Figure S98. Dose-response curves (% growth versus sample concentration) for all cell lines with different subpanel obtained from the NCI's in vitro disease-oriented human cancer cells line for compound **10e** on nine types of cancer

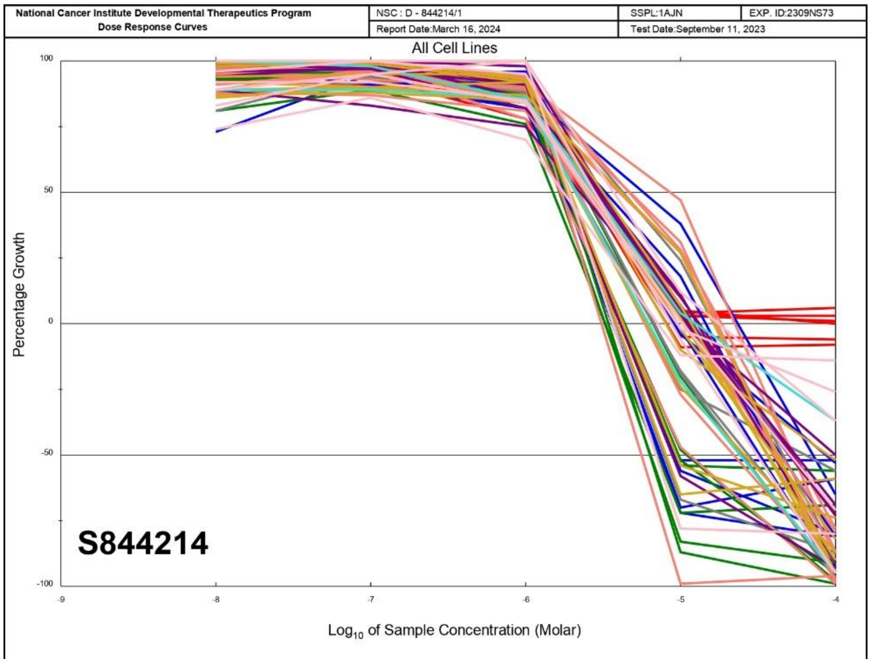

Figure S99. Dose-response curves for all cell lines in the NCI60 panel exposed compound **10e** with tissue originated colors and shapes.

Developmental Therapeutics Program<br>One Dose Mean Graph	NSC: D-844215 / 1	Conc: 1.00E-5 Molar	Test Date: Apr 10, 2023
	Experiment ID: 2304OS17		Report Date: Apr 02, 2025

Panel/Cell Line	Growth Percent
**Leukemia**	
CCRF-CEM	11.79
HL-60(TB)	7.59
K-562	5.34
MOLT-4	10.33
RPMI-8226	9.72
SR	1.64
**Non-Small Cell Lung Cancer**	
A549/ATCC	-22.89
EKVX	-26.01
HOP-62	-60.65
NCI-H226	-29.73
NCI-H23	-62.95
NCI-H322M	18.89
NCI-H460	-52.50
NCI-H522	-68.66
**Colon Cancer**	
COLO 205	-71.07
HCC-2998	-85.44
HCT-116	-32.35
HCT-15	-71.62
HT29	-56.26
KM12	-85.30
SW-620	-78.87
**CNS Cancer**	
SF-268	7.21
SF-295	-56.89
SF-539	-32.93
SNB-19	10.71
SNB-75	28.33
U251	-18.13
**Melanoma**	
LOX IMVI	-97.64
MALME-3M	-57.07
M14	8.20
MDA-MB-435	-45.31
SK-MEL-2	-20.58
SK-MEL-28	-42.45
SK-MEL-5	47.35
UACC-257	4.64
UACC-62	45.19
**Ovarian Cancer**	
IGROV1	-26.16
OVCAR-3	-28.65
OVCAR-4	9.47
OVCAR-5	8.92
OVCAR-8	1.37
NCI/ADR-RES	-31.70
SK-OV-3	22.23
**Renal Cancer**	
786-0	19.66
A498	-59.75
ACHN	14.79
CAKI-1	-5.88
RXF 393	-49.24
SN12C	-93.73
TK-10	42.32
UO-31	-18.27
**Prostate Cancer**	
DU-145	-36.82
**Breast Cancer**	
MCF7	-34.42
MDA-MB-231/ATCC	-76.51
HS 578T	-4.69
BT-549	-1.35
T-47D	0.17
MDA-MB-468	2.65
Mean	-22.48
Delta	75.16
Range	144.99

Mean Growth Percent - Growth Percent

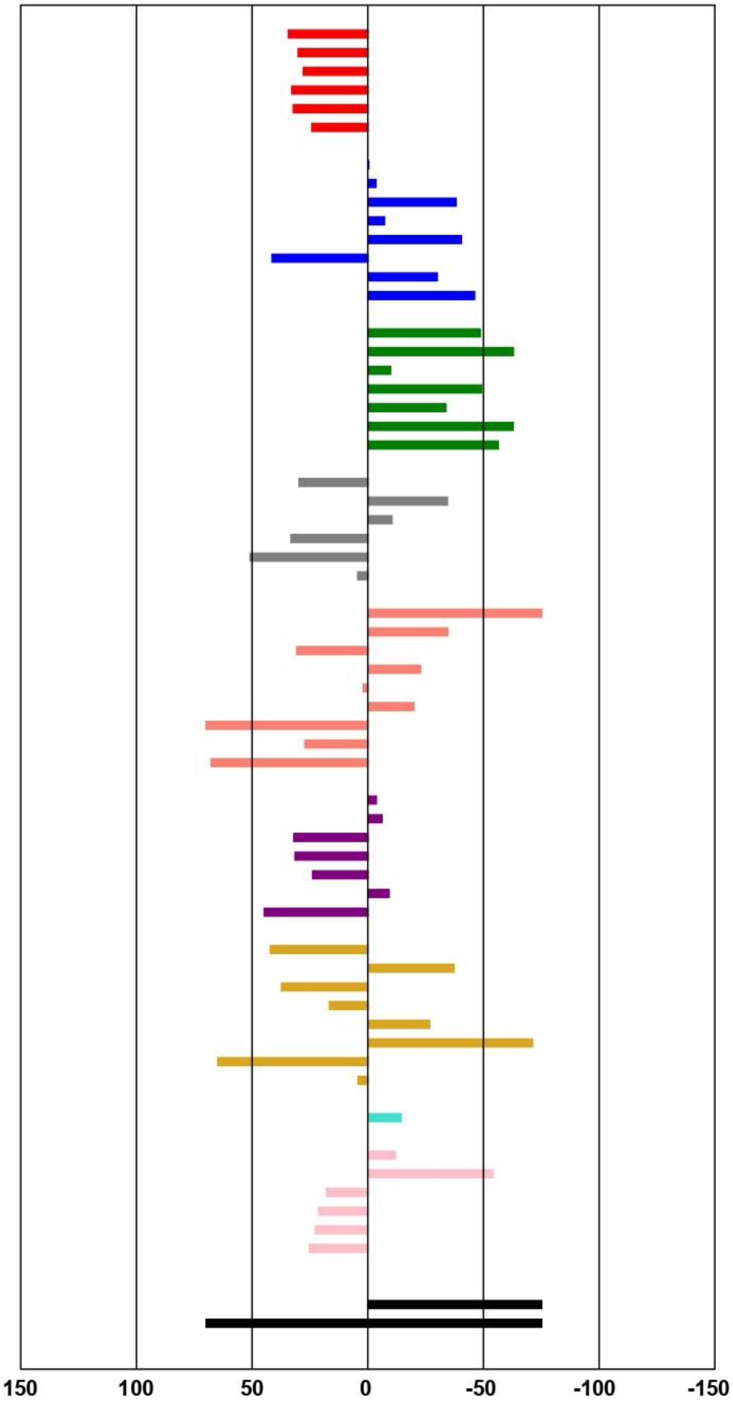

Figure S100. One dose mean graph for compound **10f** at 10 μM

National Cancer Institute Developmental Therapeutics Program
In-Vitro Testing Results

NSC : D - 844215 / 1	Experiment ID : 2309NS73	Test Type : 08	Units : Molar
Report Date : March 16, 2024	Test Date : September 11, 2023	QNS :	MC :
COMI : H1-10f	Stain Reagent : SRB Dual-Pass Related	SSPL : 1AJN	

			Log10 Concentration												
	Time		Mean Optical Densities					Percent Growth							
Panel/Cell Line	Zero	Ctrl	-8.0	-7.0	-6.0	-5.0	-4.0	-8.0	-7.0	-6.0	-5.0	-4.0	GI50	TGI	LC50
**Leukemia**															
CCRF-CEM	0.517	2.872	2.830	2.806	2.627	0.642	0.487	98	97	90	5	-6	2.95E-6	3.00E-5	> 1.00E-4
HL-60(TB)	0.600	2.958	2.798	2.827	2.653	0.556	0.539	93	94	87	-7	-10	2.47E-6	8.36E-6	> 1.00E-4
K-562	0.212	2.397	2.352	2.134	1.804	0.228	0.270	98	88	73	1	3	2.07E-6	> 1.00E-4	> 1.00E-4
MOLT-4	0.451	2.544	2.449	2.372	2.000	0.460	0.459	95	92	74	0	0	2.12E-6	> 1.00E-4	> 1.00E-4
RPMI-8226	0.732	2.631	2.498	2.524	2.336	0.685	0.620	93	94	84	-6	-15	2.39E-6	8.50E-6	> 1.00E-4
SR	0.605	2.629	2.500	2.412	2.395	0.465	0.478	94	89	88	-23	-21	2.21E-6	6.20E-6	> 1.00E-4
**Non-Small Cell Lung Cancer**															
A549/ATCC	0.313	2.329	2.109	2.110	2.067	0.396	0.147	89	89	87	4	-53	2.80E-6	1.18E-5	8.79E-5
EKVX	0.849	2.324	2.174	2.278	2.167	0.715	0.090	90	97	89	-16	-89	2.37E-6	7.07E-6	2.91E-5
HOP-62	0.895	2.472	2.237	2.410	2.405	0.409	0.487	85	96	96	-54	-46	2.02E-6	4.34E-6	.
HOP-92	1.140	1.806	1.756	1.788	1.731	1.108	0.484	92	97	89	-3	-58	2.65E-6	9.32E-6	7.28E-5
NCI-H226	0.899	1.813	1.730	1.757	1.734	1.110	0.305	91	94	91	23	-66	4.03E-6	1.81E-5	6.59E-5
NCI-H23	0.639	2.082	2.022	2.071	1.951	0.249	0.182	96	99	91	-61	-72	1.86E-6	3.96E-6	8.45E-6
NCI-H322M	0.785	2.249	2.138	2.234	2.040	0.931	0.065	92	99	86	10	-92	2.96E-6	1.25E-5	3.89E-5
NCI-H460	0.302	2.695	2.701	2.596	2.533	0.117	0.134	100	96	93	-61	-56	1.90E-6	4.01E-6	8.46E-6
NCI-H522	1.442	3.255	3.147	3.213	3.135	0.750	0.321	94	98	93	-48	-78	2.03E-6	4.58E-6	1.17E-5
**Colon Cancer**															
COLO 205	0.714	2.441	2.430	2.436	2.413	0.128	0.121	99	100	98	-82	-83	1.85E-6	3.51E-6	6.64E-6
HCC-2998	0.978	3.214	3.079	3.098	3.250	0.156	0.128	94	95	102	-84	-87	1.90E-6	3.52E-6	6.55E-6
HCT-116	0.341	2.806	2.723	2.739	2.537	0.095	0.161	97	97	89	-72	-53	1.75E-6	3.57E-6	7.29E-6
HCT-15	0.333	2.569	2.451	2.459	2.433	0.121	0.010	95	95	94	-64	-97	1.90E-6	3.94E-6	8.17E-6
HT29	0.260	1.918	1.782	1.902	1.863	0.044	0.025	92	99	97	-83	-91	1.82E-6	3.45E-6	6.55E-6
KM12	0.590	2.646	2.456	2.546	2.453	0.116	0.112	91	95	91	-80	-81	1.73E-6	3.39E-6	6.65E-6
SW-620	0.342	1.949	2.002	2.188	2.204	0.283	0.389	103	115	116	-17	3	3.12E-6	.	> 1.00E-4
**CNS Cancer**															
SF-268	0.855	2.408	2.315	2.338	2.216	0.833	0.400	94	96	88	-3	-53	2.61E-6	9.36E-6	8.62E-5
SF-295	0.900	2.861	2.617	2.640	2.677	0.612	0.093	88	89	91	-32	-90	2.14E-6	5.48E-6	2.05E-5
SF-539	0.649	2.343	2.253	2.285	2.165	0.245	0.246	95	97	89	-62	-62	1.82E-6	3.89E-6	8.29E-6
SNB-19	0.664	2.619	2.445	2.502	2.376	0.962	0.303	91	94	88	15	-54	3.31E-6	1.66E-5	8.65E-5
SNB-75	0.911	1.421	1.359	1.432	1.404	0.842	0.380	88	102	97	-8	-58	2.81E-6	8.46E-6	6.86E-5
U251	0.243	1.560	1.452	1.441	1.404	0.042	0.055	92	91	88	-83	-77	1.67E-6	3.28E-6	6.42E-6
**Melanoma**															
LOX IMVI	0.506	2.793	2.613	2.661	2.591	0.005	0.128	92	94	91	-99	-75	1.65E-6	3.01E-6	5.52E-6
MALME-3M	0.576	1.123	1.026	1.047	1.031	0.211	0.005	82	86	83	-63	-99	1.68E-6	3.69E-6	8.10E-6
M14	0.541	2.250	2.192	2.187	2.089	0.389	0.091	97	96	91	-28	-83	2.20E-6	5.79E-6	2.49E-5
MDA-MB-435	0.630	2.260	2.165	2.138	2.084	0.454	0.216	94	93	89	-28	-66	2.16E-6	5.77E-6	3.83E-5
SK-MEL-2	1.280	2.528	2.418	2.515	2.438	1.668	0.044	91	99	93	31	-97	4.93E-6	1.75E-5	4.32E-5
SK-MEL-28	0.641	2.162	2.159	2.109	1.980	0.732	0.262	100	96	88	6	-59	2.91E-6	1.24E-5	7.24E-5
SK-MEL-5	0.933	3.096	2.803	2.874	2.894	1.120	0.044	86	90	91	9	-95	3.13E-6	1.21E-5	3.66E-5
UACC-257	1.011	2.688	2.495	2.564	2.483	1.593	0.264	88	93	88	35	-74	5.15E-6	2.09E-5	6.02E-5
UACC-62	0.846	3.075	2.993	2.972	2.732	1.750	0.263	96	95	85	41	-69	6.10E-6	2.34E-5	6.71E-5
**Ovarian Cancer**															
IGROV1	0.511	2.211	2.242	2.210	1.994	0.556	0.121	102	100	87	3	-76	2.75E-6	1.08E-5	4.63E-5
OVCAR-3	0.495	1.577	1.583	1.574	1.522	0.205	0.070	100	100	95	-59	-86	1.96E-6	4.15E-6	8.79E-6
OVCAR-4	0.875	2.017	2.001	2.032	1.939	0.813	0.262	99	101	93	-7	-70	2.69E-6	8.50E-6	4.80E-5
OVCAR-5	0.691	2.506	2.505	2.565	2.410	0.927	0.222	100	103	95	13	-68	3.53E-6	1.45E-5	6.00E-5
OVCAR-8	0.419	2.312	2.240	2.311	2.240	0.379	0.086	96	100	96	-10	-79	2.73E-6	8.10E-6	3.78E-5
NCI/ADR-RES	0.527	1.957	1.930	1.982	1.899	0.400	0.279	98	102	96	-24	-47	2.41E-6	6.30E-6	> 1.00E-4
SK-OV-3	0.803	1.718	1.663	1.735	1.812	0.852	0.254	94	102	110	5	-68	3.75E-6	1.18E-5	5.63E-5
**Renal Cancer**															
786-0	0.761	2.988	2.991	3.027	2.965	0.275	0.318	100	102	99	-64	-58	2.00E-6	4.05E-6	8.21E-6
A498	1.228	2.325	2.130	2.192	2.138	0.971	0.307	82	88	83	-21	-75	2.08E-6	6.29E-6	3.45E-5
ACHN	0.295	1.712	1.598	1.631	1.407	0.353	0.107	92	94	78	4	-64	2.41E-6	1.15E-5	6.27E-5
CAKI-1	0.802	2.484	2.335	2.415	2.224	0.784	0.208	91	96	85	-2	-74	2.50E-6	9.41E-6	4.61E-5
RXF 393	0.864	1.686	1.645	1.688	1.655	0.608	0.278	95	100	96	-30	-68	2.33E-6	5.81E-6	3.41E-5
SN12C	0.613	2.945	2.675	2.672	2.521	0.139	0.202	88	88	82	-77	-67	1.58E-6	3.26E-6	6.73E-6
TK-10	1.357	2.315	2.148	2.232	2.272	1.587	0.142	83	91	96	24	-90	4.33E-6	1.63E-5	4.48E-5
UO-31	0.493	1.946	1.731	1.629	1.691	0.278	0.177	85	78	82	-44	-64	1.81E-6	4.50E-6	2.03E-5
**Prostate Cancer**															
PC-3	0.613	2.097	1.978	1.995	1.852	0.636	0.364	92	93	83	2	-41	2.56E-6	1.09E-5	> 1.00E-4
DU-145	0.406	1.888	1.752	1.789	1.617	0.209	0.054	91	93	82	-49	-87	1.75E-6	4.23E-6	1.09E-5
**Breast Cancer**															
MCF7	0.509	2.465	2.288	2.266	2.279	0.375	0.207	91	90	90	-26	-59	2.22E-6	5.95E-6	5.19E-5
MDA-MB-231/ATCC	0.522	1.542	1.440	1.424	1.300	0.108	0.125	90	88	76	-79	-76	1.48E-6	3.09E-6	6.48E-6
HS 578T	0.945	2.132	1.912	2.089	1.962	1.121	1.021	81	96	86	15	6	3.19E-6	> 1.00E-4	> 1.00E-4
BT-549	1.295	2.614	2.496	2.613	2.585	0.747	0.310	91	100	98	-42	-76	2.19E-6	4.99E-6	1.69E-5
T-47D	0.533	1.150	1.138	1.142	1.133	0.635	0.590	98	99	97	16	9	3.85E-6	> 1.00E-4	> 1.00E-4
MDA-MB-468	0.726	1.474	1.451	1.502	1.401	0.556	0.087	97	104	90	-23	-88	2.26E-6	6.21E-6	2.57E-5

Figure S101. Values of log molar concentration of response parameters ($\log_{10}$ GI_{50}, $\log_{10}$ TGI & $\log_{10}$ LC_{50}) for compound **10f**

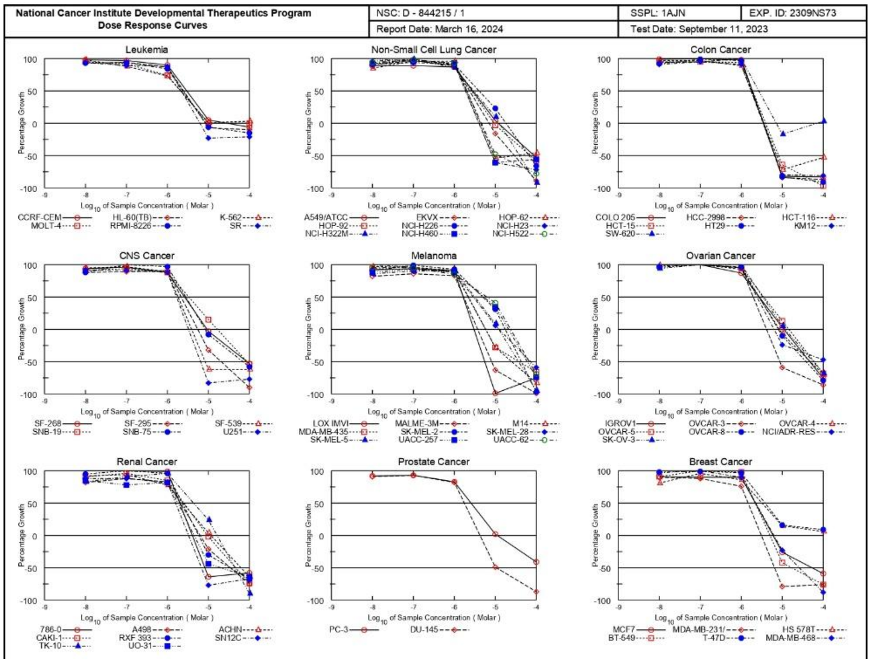

Figure S102. Dose-response curves (% growth versus sample concentration) for all cell lines with different subpanel obtained from the NCI's in vitro disease-oriented human cancer cells line for compound **10f** on nine types of cancer

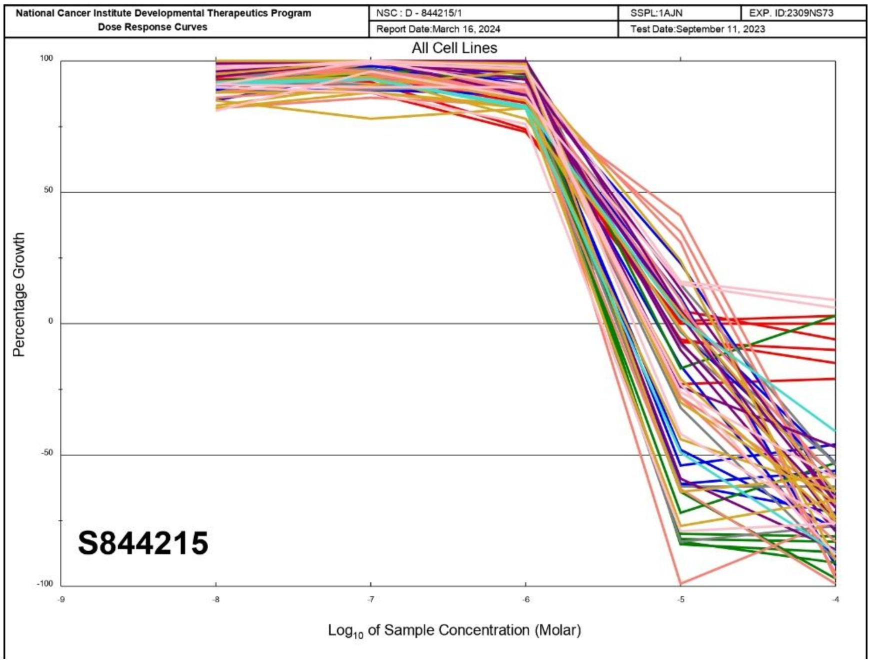

Figure S103. Dose-response curves for all cell lines in the NCI60 panel exposed compound **10f** with tissue originated colors and shapes.

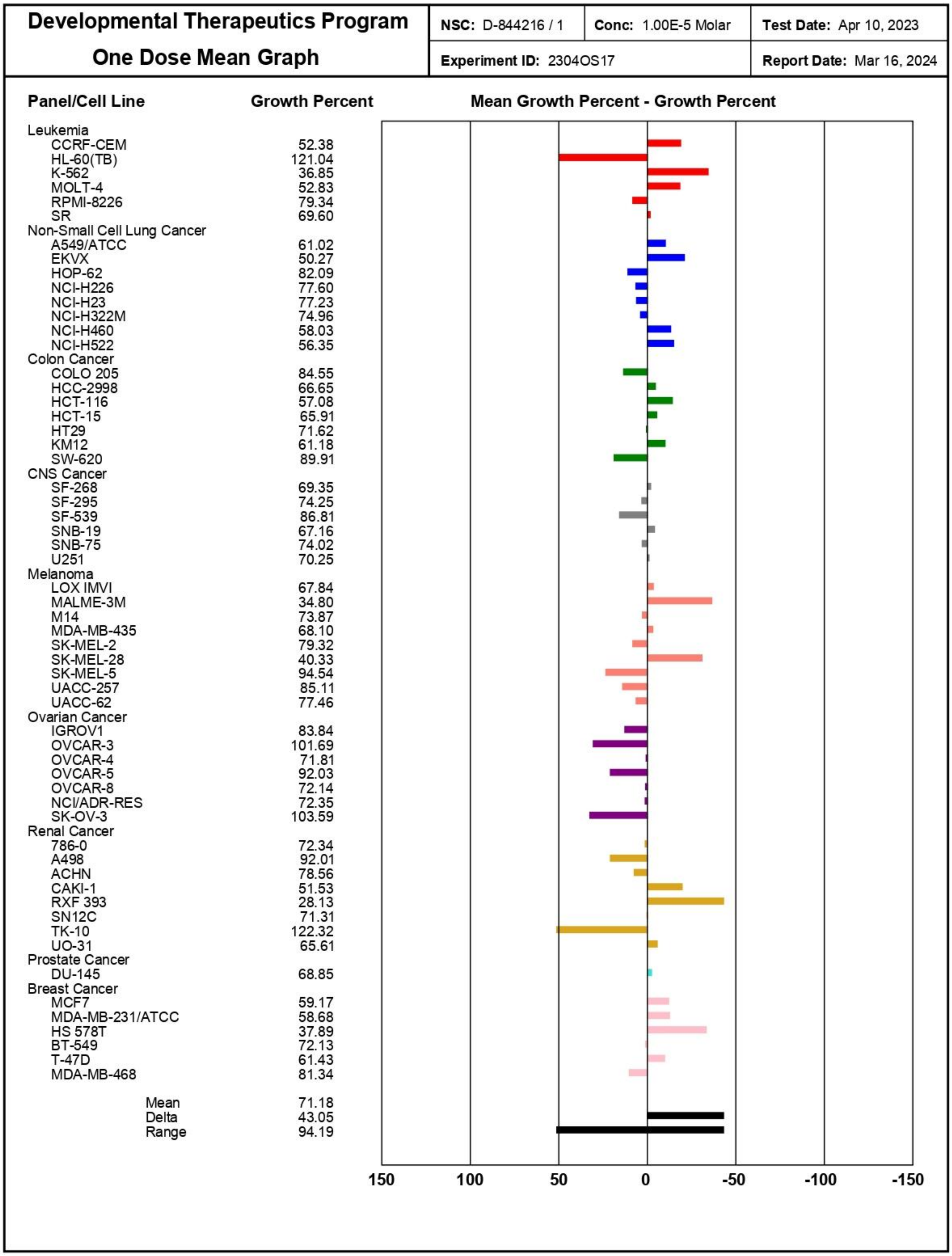

Developmental Therapeutics Program
One Dose Mean Graph

NSC: D-844216 / 1	Conc: 1.00E-5 Molar	Test Date: Apr 10, 2023
Experiment ID: 2304OS17		Report Date: Mar 16, 2024

Panel/Cell Line	Growth Percent
**Leukemia**	
CCRF-CEM	52.38
HL-60(TB)	121.04
K-562	36.85
MOLT-4	52.83
RPMI-8226	79.34
SR	69.60
**Non-Small Cell Lung Cancer**	
A549/ATCC	61.02
EKVX	50.27
HOP-62	82.09
NCI-H226	77.60
NCI-H23	77.23
NCI-H322M	74.96
NCI-H460	58.03
NCI-H522	56.35
**Colon Cancer**	
COLO 205	84.55
HCC-2998	66.65
HCT-116	57.08
HCT-15	65.91
HT29	71.62
KM12	61.18
SW-620	89.91
**CNS Cancer**	
SF-268	69.35
SF-295	74.25
SF-539	86.81
SNB-19	67.16
SNB-75	74.02
U251	70.25
**Melanoma**	
LOX IMVI	67.84
MALME-3M	34.80
M14	73.87
MDA-MB-435	68.10
SK-MEL-2	79.32
SK-MEL-28	40.33
SK-MEL-5	94.54
UACC-257	85.11
UACC-62	77.46
**Ovarian Cancer**	
IGROV1	83.84
OVCAR-3	101.69
OVCAR-4	71.81
OVCAR-5	92.03
OVCAR-8	72.14
NCI/ADR-RES	72.35
SK-OV-3	103.59
**Renal Cancer**	
786-0	72.34
A498	92.01
ACHN	78.56
CAKI-1	51.53
RXF 393	28.13
SN12C	71.31
TK-10	122.32
UO-31	65.61
**Prostate Cancer**	
DU-145	68.85
**Breast Cancer**	
MCF7	59.17
MDA-MB-231/ATCC	58.68
HS 578T	37.89
BT-549	72.13
T-47D	61.43
MDA-MB-468	81.34
Mean	71.18
Delta	43.05
Range	94.19

Figure S104. One dose mean graph for compound **10g** at 10 μM

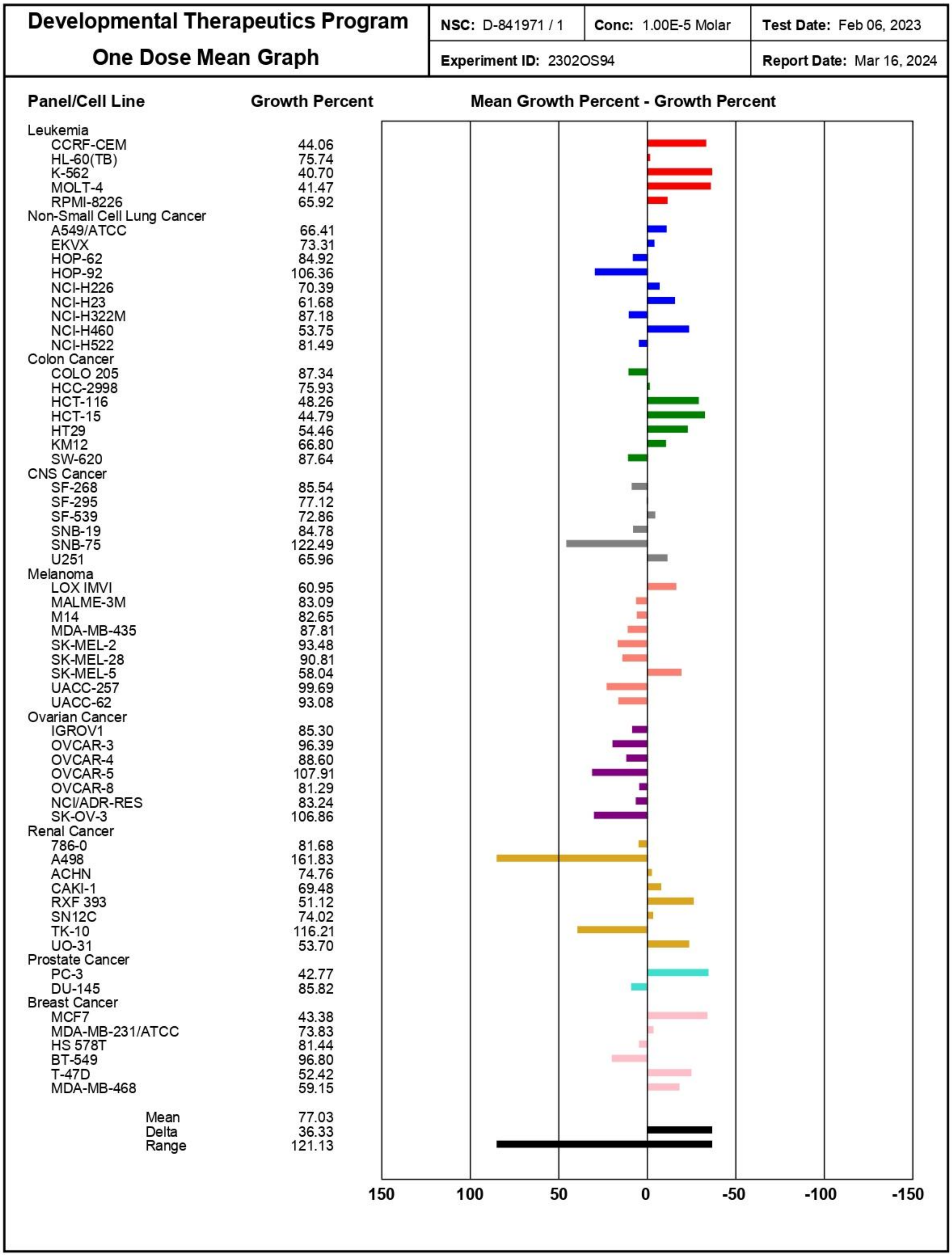

Figure S105. One dose mean graph for compound **12** at 10 μM

Table S1. Kinases assay

Code	Abl Residual (%)			FLT3-ITD Residual (%)			PDGFR Residual (%)		
	10 µM	1 µM	0.1 µM	10 µM	1 µM	0.1 µM	10 µM	1 µM	0.1 µM
**8b**	108	93	94	67	89	111	110	112	94
**8c**	88	81	78	93	90	95	-	-	-
**8e**	104	75	74	82	111	113	-	-	-
**8f**	84	80	72	97	108	111	129	110	107
**10b**	84	77	67	89	98	104	89	110	91
**10c**	97	76	65	72	109	105	98	90	95
**10e**	93	79	72	90	124	123	-	-	-
**10f**	98	101	74	111	123	128	108	101	94

Biological evaluation

In vitro antitumor screening against 60 cancer cell lines

The *in vitro* anticancer evaluation was carried out against a panel of 60 human tumor cell lines derived from nine different tissue types, including leukemia, lung, colon, central nervous system, melanoma, ovarian, renal, prostate, and breast cancers, in compliance with the standard protocol set by the Drug Evaluation Branch of the National Cancer Institute (NCI), Bethesda, MD. Key dose-response parameters, including GI_{50} (the dosage needed to inhibit 50% of cell growth), TGI (total growth inhibition), and LC_{50} (the concentration fatal to 50% of the cells), were computed after each compound's antiproliferative activity was evaluated. The potency and cytotoxic profile of each produced molecule were evaluated using these values, which are described as follows [1-9].

Cell lines

The MV4-11 cancer cell line was purchased from the German Collection of Microorganisms and K562 from the European Collection of Cell Cultures. All cell lines were cultivated according to the provider's instructions, kept in RPMI-1640 or DMEM medium accompanied with 10% fetal bovine serum, 100 U/mL penicillin, and 100 mg/mL streptomycin and cultivated in a humidified CO_2 incubator at 37 °C.

Supporting Information

Cell viability assay

Cells were planted into 96-well plates and incubated with increasing concentrations of tested compounds for 72 hours. Following treatment, resazurin (Merck) was added to each well at a final concentration of 10.5 μg/mL and incubated for 4 hours. The fluorescence of the reduced product, resorufin, was measured at 544 nm (excitation) and 590 nm (emission) using a Fluoroskan Ascent microplate reader (Labsystems). GI_{50} values, defined as the compound concentration lethal to 50% of the cancer cells, were calculated from the obtained dose-response curves.

Flow cytometry

Asynchronously growing cells were incubated with tested compound at different concentrations. After 24 hours, cells were harvested, fixed with 70% ethanol in ice-cold, and incubated on ice for 30 min. Then, they were washed with PBS, stained with propidium iodide for 30 minutes, and analyzed by flow cytometry using a 488 nm laser (BD FACS Verse). Cell cycle distribution was quantified using ModFit LT (Verity Software House, version 5.0.9).

Immunoblotting

Cell lysates were prepared using extraction RIPA buffer, and proteins were separated by SDS-polyacrylamide gel electrophoresis followed by an electrotransfer onto nitrocellulose membranes. The membranes were then blocked for 1 hour and incubated overnight at 4 °C with specific primary antibodies. After washing, membranes were incubated for 1 hour with peroxidase-conjugated secondary antibodies. Peroxidase activity was detected using SuperSignal West Pico reagents (Thermo Scientific) using a LAS-4000 CCD camera (Fujifilm). Specific antibodies were purchased from Cell Signaling Technology (peroxidase-conjugated secondary antibodies; anti-PARP-1, clone 46D11; anti-Mcl-1, clone D35A5; anti-XIAP; anti-Bax, clone D2E11; anti-Caspase 7; anti-Caspase 9), Santa Cruz Biotechnology (anti-β-actin, clone C4; anti-Caspase 3, clone 31A1067) and Merck (anti-phospho-Histone H2AX-Ser139).

Topoisomerase relaxation assay

The topoisomerase I reaction was performed with topoisomerase I (Inspiralis) in the presence of 0.5 μg supercoiled pBR322 in assay buffer (20 mM Tris-HCl pH 7.5, 200 mM NaCl, 0.25 mM EDTA, 5% glycerol, 50 μg/μL albumin) in a total reaction volume of 30 μL at 37 ºC and 350 rpm for 30 minutes. The topoisomerase II reaction was performed with topoisomerase IIα (Inspiralis) in the presence of 0.5 μg supercoiled pBR322 and 1 mM ATP in assay buffer (50 mM

Tris-HCl pH 7.5, 125 mM NaCl, 10 mM $MgCl_2$, 5 mM DTT, 100 µg/µL albumin) in a total reaction volume of 30 µL at 37 ºC and 350 rpm for 30 minutes. In both experiments, the reaction was stopped by adding 30 µL of GSTEB (8% (w/v) glycerol, 25 mM Tris-HCl pH 8.0, 2 mM EDTA, 0.1 mg/mL bromphenol blue) and 30 µL of chloroform/isoamyl alcohol (v:v, 24:1). The reaction products were separated by 5% agarose gel electrophoresis and visualized using GelRed nucleic acid stain (Biotium). The relaxation level of pBR322 was visualized by an FLA-7000 digital image analyzer (FujiFilm).

References

1. Elsebaie, H. A.; El-Bastawissy, E. A.; Elberembally, K. M.; Khaleel, E. F.; Badi, R. M.; Shaldam, M. A.; Eldehna, W. M.; Tawfik, H. O.; El-Moselhy, T. F., Novel 4-(2-arylidenehydrazineyl)thienopyrimidine derivatives as anticancer EGFR inhibitors: Design, synthesis, biological evaluation, kinome selectivity and in silico insights. *Bioorganic Chemistry* **2023,** *140*, 106799.
2. Tawfik, H. O.; Mousa, M. H. A.; Zaky, M. Y.; El-Dessouki, A. M.; Sharaky, M.; Abdullah, O.; El-Hamamsy, M. H.; Al-Karmalawy, A. A., Rationale design of novel substituted 1,3,5-triazine candidates as dual IDH1(R132H)/ IDH2(R140Q) inhibitors with high selectivity against acute myeloid leukemia: In vitro and in vivo preclinical investigations. *Bioorganic Chemistry* **2024,** *149*, 107483.
3. Abo Al-Hamd, M. G.; O., T. H.; Omeima, A.; Koki, Y.; Masaharu, S.; M., M. A. B.; H., E.-H. M.; and El-Moselhy, T. F., Recruitment of hexahydroquinoline as anticancer scaffold targeting inhibition of wild and mutants EGFR (EGFRWT, EGFRT790M, and EGFRL858R). *Journal of Enzyme Inhibition and Medicinal Chemistry* **2023,** *38* (1), 2241674.
4. Aboukhatwa, S. M.; Sidhom, P. A.; Angeli, A.; Supuran, C. T.; Tawfik, H. O., Terminators or Guardians? Design, Synthesis, and Cytotoxicity Profiling of Chalcone-Sulfonamide Hybrids. *ACS Omega* **2023,** *8* (8), 7666-7683.
5. Tawfik, H. O.; Shaldam, M. A.; Nocentini, A.; Salem, R.; Almahli, H.; Al-Rashood, S. T.; Supuran, C. T.; Eldehna, W. M., Novel 3-(6-methylpyridin-2-yl)coumarin-based chalcones as selective inhibitors of cancer-related carbonic anhydrases IX and XII endowed with anti-proliferative activity. *Journal of Enzyme Inhibition and Medicinal Chemistry* **2022,** *37* (1), 1043-1052.

6. Eldehna, W. M.; Salem, R.; Elsayed, Z. M.; Al-Warhi, T.; Knany, H. R.; Ayyad, R. R.; Traiki, T. B.; Abdulla, M.-H.; Ahmad, R.; Abdel-Aziz, H. A.; El-Haggar, R., Development of novel benzofuran-isatin conjugates as potential antiproliferative agents with apoptosis inducing mechanism in Colon cancer. *Journal of Enzyme Inhibition and Medicinal Chemistry* **2021,** *36* (1), 1423-1434.
7. Eldehna, W. M.; Fares, M.; Bonardi, A.; Avgenikos, M.; Baselious, F.; Schmidt, M.; Al-Warhi, T.; Abdel-Aziz, H. A.; Rennert, R.; Peat, T. S.; Supuran, C. T.; Wessjohann, L. A.; Ibrahim, H. S., 4-(Pyrazolyl)benzenesulfonamide Ureas as Carbonic Anhydrases Inhibitors and Hypoxia-Mediated Chemo-Sensitizing Agents in Colorectal Cancer Cells. *Journal of Medicinal Chemistry* **2024,** *67* (22), 20438-20454.
8. Elsawi, A. E.; Elbadawi, M. M.; Nocentini, A.; Almahli, H.; Giovannuzzi, S.; Shaldam, M.; Salem, R.; Ibrahim, T. M.; Abdel-Aziz, H. A.; Supuran, C. T.; Eldehna, W. M., 1,5-Diaryl-1,2,4-triazole Ureas as New SLC-0111 Analogues Endowed with Dual Carbonic Anhydrase and VEGFR-2 Inhibitory Activities. *Journal of Medicinal Chemistry* **2023,** *66* (15), 10558-10578.
9. Elbadawi, M. M.; Eldehna, W. M.; Wang, W.; Agama, K. K.; Pommier, Y.; Abe, M., Discovery of 4-alkoxy-2-aryl-6,7-dimethoxyquinolines as a new class of topoisomerase I inhibitors endowed with potent in vitro anticancer activity. *European Journal of Medicinal Chemistry* **2021,** *215*, 113261.
